# RNA-binding protein Maca is crucial for gigantic male fertility factor gene expression, spermatogenesis, and male fertility, in *Drosophila*

**DOI:** 10.1371/journal.pgen.1009655

**Published:** 2021-06-28

**Authors:** Li Zhu, Ryuya Fukunaga

**Affiliations:** Department of Biological Chemistry, Johns Hopkins University School of Medicine, Baltimore, Maryland, United States of America; College de France CNRS, FRANCE

## Abstract

During spermatogenesis, the process in which sperm for fertilization are produced from germline cells, gene expression is spatiotemporally highly regulated. In *Drosophila*, successful expression of extremely large male fertility factor genes on Y-chromosome spanning some megabases due to their gigantic intron sizes is crucial for spermatogenesis. Expression of such extremely large genes must be challenging, but the molecular mechanism that allows it remains unknown. Here we report that a novel RNA-binding protein Maca, which contains two RNA-recognition motifs, is crucial for this process. *maca* null mutant male flies exhibited a failure in the spermatid individualization process during spermatogenesis, lacked mature sperm, and were completely sterile, while *maca* mutant female flies were fully fertile. Proteomics and transcriptome analyses revealed that both protein and mRNA abundance of the gigantic male fertility factor genes *kl-2*, *kl-3*, and *kl-5* (*kl* genes) are significantly decreased, where the decreases of *kl-2* are particularly dramatic, in *maca* mutant testes. Splicing of the *kl-3* transcripts was also dysregulated in *maca* mutant testes. All these physiological and molecular phenotypes were rescued by a *maca* transgene in the *maca* mutant background. Furthermore, we found that in the control genetic background, Maca is exclusively expressed in spermatocytes in testes and enriched at Y-loop A/C in the nucleus, where the *kl-5* primary transcripts are localized. Our data suggest that Maca increases transcription processivity, promotes successful splicing of gigantic introns, and/or protects transcripts from premature degradation, of the *kl* genes. Our study identified a novel RNA-binding protein Maca that is crucial for successful expression of the gigantic male fertility factor genes, spermatogenesis, and male fertility.

## Author summary

Sperm is produced from germline cells via the process called spermatogenesis, during which a special gene expression program dedicated to this process operates. In fruit fly *Drosophila*, extremely large male fertility factor genes on Y-chromosome containing mega-base-sized introns are expressed only during spermatogenesis, and their successful expression is crucial for spermatogenesis. However, expressing such large genes must be very challenging for cells and the molecular mechanisms that ensure their successful expression remain unknown. In this study, we identified a novel RNA-binding protein encoded in the gene *CG5213* that is required for this process. We created mutant flies lacking this protein using a genome-editing technique, and investigated them using genetic, microscopic, molecular, transcriptomic, and proteomic approaches. We found that this RNA-binding protein is crucial for successful expression of the extremely large male fertility factor genes, spermatogenesis, and male fertility. We named the *CG5213* gene *maca*, after the plant maca grown in the high Andes mountains whose root has been traditionally believed and used to increase sperm quality and promote male fertility. By identifying the novel RNA-binding protein Maca, our studies give significant insight into how the special gene expression program operates during spermatogenesis.

## Introduction

Spermatogenesis is the highly conserved and tightly regulated process where diploid germline stem cells develop into mature, haploid sperm capable of fertilizing oocytes. Spatiotemporally highly regulated gene expression is crucial during spermatogenesis, and transcriptional and post-transcriptional regulation by RNA-binding proteins play key roles. Human male sterility results from abnormal spermatogenesis and is mostly due to chromosomal alterations, Y chromosome microdeletions, and related gene mutations. Mutations in the germline-specific DAZ (Deleted in AZoospermia) family of RNA-binding proteins, which contain a highly conserved RNA recognition motif (RRM) and are crucial regulators of gene expression during spermatogenesis, impair spermatogenesis and cause human male sterility [1–12].

**Fig 1:**
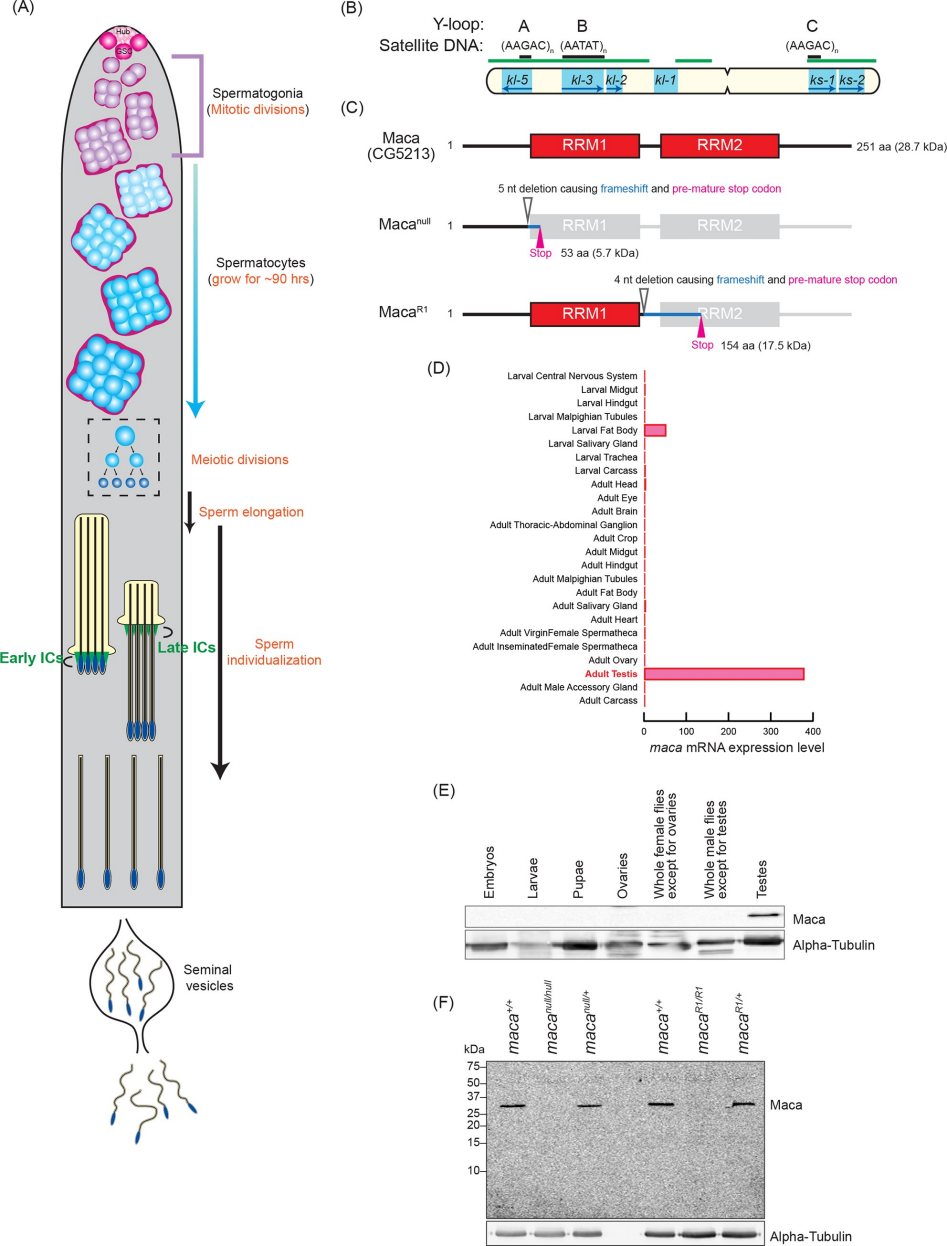
Maca domain structure, mutant alleles, and expression pattern. (A) Diagram of spermatogenesis in *Drosophila* testis. At the apical tip of testis, germline stem cells (GSCs) are attached to the hub cells (Hub). GSCs produce daughter cell gonialblasts, which become mitotically-amplifying spermatogonia that then become spermatocytes. Spermatocytes grow in size for ~90 hours and then undergo the meiotic divisions to become 64 interconnected spermatids, which are then separated into individual spermatids by individualization complexes (ICs). Individualized mature motile sperm are stored in seminal vesicles. (B) Diagram of the *Drosophila* Y chromosome. Locations of the 6 male fertility factor genes (cyan. The transcription directions of the encoded genes are indicated by blue arrows), regions enriched for satellite DNA (green bars), and the Y-loop forming regions (black bars) with associated satellite DNA sequences are shown. (C) Domain structures of *Drosophila* Maca (Maca/CG5213), and its mutants, Maca^null^ and Maca^R1^. (D) *maca* mRNA expression pattern. Data are obtained from http://flybase.org/reports/FBgn0038345. (E) Western blots of dissected fly tissues. (F) Western blots of testis lysates.

Genetically well-tractable *Drosophila* has been an excellent model system for spermatogenesis studies [13–16]. The *Drosophila* testis, where spermatogenesis occurs, is a coiled tube with a closed apical end and a basal end that connects to the seminal vesicle. The germline stem cells (GSCs) reside at the apical tip attached to the hub cells and differentiating germ cells are gradually displaced distally (Fig 1A). GSCs divide asymmetrically to produce a self-renewed GSC and a daughter cell that undergoes differentiation and becomes 16 spermatogonia by four rounds of synchronous mitotic divisions. The 16-cell spermatogonia then enter the meiotic S phase and become spermatocytes. Spermatocytes grow in size during their G2 growth phase, which spans as long as ~90 hours. During the spermatocyte growth, the homologous chromosomes pair and partition into individual chromosome territories, and almost all genes encoding proteins for later meiotic and post-meiotic processes (spermiogenesis) are transcribed. These transcripts are stored without being efficiently translated until their protein activities are required later for meiosis and spermiogenesis. Thus, transcriptional and post-transcriptional events during the spermatocyte growth phase are crucial and must be spatiotemporally highly regulated.

After their G2 growth phase, spermatocytes divide twice by meiosis and then differentiate into 64 interconnected haploid spermatids. After 64 interconnected spermatids elongate, they are separated into individual spermatids by individualization complexes (ICs) in the sperm individualization process (Fig 1A). Individualization complexes form around the sperm nuclei and move processively in a highly coordinated manner from the heads to the tips of the sperm tails along the spermatid bundle, removing excess cytoplasm and unneeded organelles and encasing each sperm cell in its own plasma membrane. The individualized, mature motile sperm are stored in the seminal vesicles for fertilization.

The ~40 Mb Y chromosome of *Drosophila* is required only for male fertility, but not fly viability, mostly (~80%) comprised of repetitive sequences (primarily short tandem repeat satellite DNAs), and entirely heterochromatic [17–19]. Because of the extremely repetitive nature, the genome DNA sequence of most of the Y chromosome is not fully determined yet and available genome sequences of the Y chromosome contain many gaps. Despite the considerable size of the Y chromosome, it has only 16 known protein-coding genes. The Y chromosome contains six loci called male fertility factors that are essential for spermatogenesis and male fertility (Fig 1B) [18,20–22]. Four (*kl-5*, *kl-3*, *kl-2*, and *kl-1*) are on the long arm of the Y and two (*ks-1* and *ks-2*) are on the short arm. *kl-2*, *kl-3*, and *kl-5* encode dynein proteins that are crucial components of the axoneme, the microtubule-based cytoskeletal structure that forms the core of a sperm flagellum [23–28]. The *kl-2*, *kl-3*, and *kl-5* genes are transcribed during the spermatocyte growth and their mRNAs form cytoplasmic ribonucleoprotein (RNP) granules called kl-granules in late-stage spermatocytes [29]. *kl-2*, *kl-3*, and *kl-5* mRNAs are suggested to start being efficiently translated only when axoneme elongates in the sperm elongation process in spermiogenesis. The male fertility factor genes including *kl-2*, *kl-3*, and *kl-5* contain gigantic, megabase-sized introns rich in repetitive, satellite DNA. For example, *kl-3* gene spans at least 4.3 Mb while its coding sequence is only ~14 kb [18,30,31]. Their gene sizes and structures are reminiscent of those of human Dystrophin, a causative gene for Duchenne Muscular Dystrophy, which spans ~2.2 Mb with gigantic introns rich in repetitive DNA and only ~11 kb coding sequence [32]. Transcription of these extremely large genes and processing their transcripts including splicing must be challenging for cells. There must be unknown special molecular mechanisms and factors that enable their efficient, precise, and regulated transcription and processing.

In spermatocytes, *kl-3*, *kl-5*, and *ks-1* form lampbrush-like nucleoplasmic structures named Y-loops [denoted as loops A (kl-5), B (kl-3), and C (ks-1) (Fig 1B), analogous to the lampbrush loops of amphibian oocytes [33]. Y-loop structures reflect the robust transcription of underlying genes in spermatocytes. Each loop consists of a DNA axis associated with huge repetitive RNA transcripts, which are in turn associated with large amounts of proteins [33–35].

In this study, we hypothesized that there may be a previously uncharacterized RNA-binding protein that is important for the testis-specific gene expression program. We predicted that such an RNA-binding protein, if any, is expressed exclusively in testes. We found that the gene *CG5213*, located on the right of the third chromosome, meets these criteria: it encodes a novel RNA-binding protein with two RNA recognition motifs (RRMs) (Fig 1C) and its mRNA is expressed almost exclusively in adult testes (Fig 1D). We genetically investigated the functions of *CG5213* and revealed that it is required for spermatogenesis and male fertility in *Drosophila*. We discovered that the CG5213 protein is expressed in spermatocytes, resides in the nucleus enriched at Y-loop A/C, and is required for successful expression and splicing of the *kl-2*, *kl-3*, and *kl-5* transcripts. We named the *CG5213* gene *maca*, after the plant maca grown in the high Andes mountains whose root has been traditionally believed and used to increase sperm quality and promote male fertility.

## Results

### Maca is exclusively expressed in testes

We generated a polyclonal anti-Maca antibody against a recombinant full-length Maca protein and examined Maca protein expression in *Drosophila* tissues using hand-dissected control (*w*^*1118*^) fly samples. We found that Maca protein is expressed exclusively in adult testes (Fig 1E).

### *maca* mutant flies

To study biological and molecular functions of Maca in vivo, we created two *maca* mutant alleles, *maca*^*null*^ and *maca*^*R1*^ by introducing deletions within the Maca coding region using a CRISPR/Cas9 genome editing system (Fig 1C) [36]. The *maca*^*null*^ allele has a 5-nt long deletion before the first RRM (deletion of 135–139 nucleotide residues in the Maca coding sequence (CDS)), which caused a translation frameshift and produced a premature stop codon resulting in encoding an N-terminal 45 amino acid (aa) fragment of Maca followed by additional 8 aa (total 53 aa, 5.7 kDa. Fig 1C). This short fragment is unlikely to have any functions and we consider this allele as null. The *maca*^*R1*^ allele has a 4-nt long deletion after the first RRM (deletion of 353–356 nucleotide residues in the Maca CDS), causing a translation frameshift and producing a premature stop codon. The *maca*^*R1*^ allele encodes an N-terminal 117 aa fragment of Maca followed by additional 37 aa (total 154 aa, 17.5 kDa. Fig 1C). Both *maca*^*null*^ and maca^*R1*^ homozygous mutant flies were viable, showing that Maca is dispensable for fly viability.

To validate the *maca* mutant fly strains and the Maca antibody that we created, we performed Western blots of testis lysates from the *maca* mutant flies using the anti-Maca antibody. In testis lysates of wild-type (*maca*^*+/+*^) and heterozygous controls of *maca*^*null*^ and *maca*^*R1*^ (*maca*^*null/+*^ and *maca*^*R1/+*^), the full-length Maca protein was detected as expected (Fig 1F). The full-length Maca protein was not detected in the testis lysates from the homozygous mutants of *maca*^*null*^ and *maca*^*R1*^ (*maca*^*null/null*^ and *maca*^*R1/R1*^). These results validated our *maca* mutant fly strains and anti-Maca antibody. No smaller proteins corresponding to the Maca fragments were detected in the *maca*^*null*^ and *maca*^*R1*^ strains (*maca*^*null/+*^, *maca*^*null/null*^, *maca*^*R1/+*^, and *maca*^*R1/R1*^). Therefore, the Maca fragments encoded in these two mutant alleles are likely unstable or not expressed.

### *maca* is essential for male fertility

**Fig 2:**
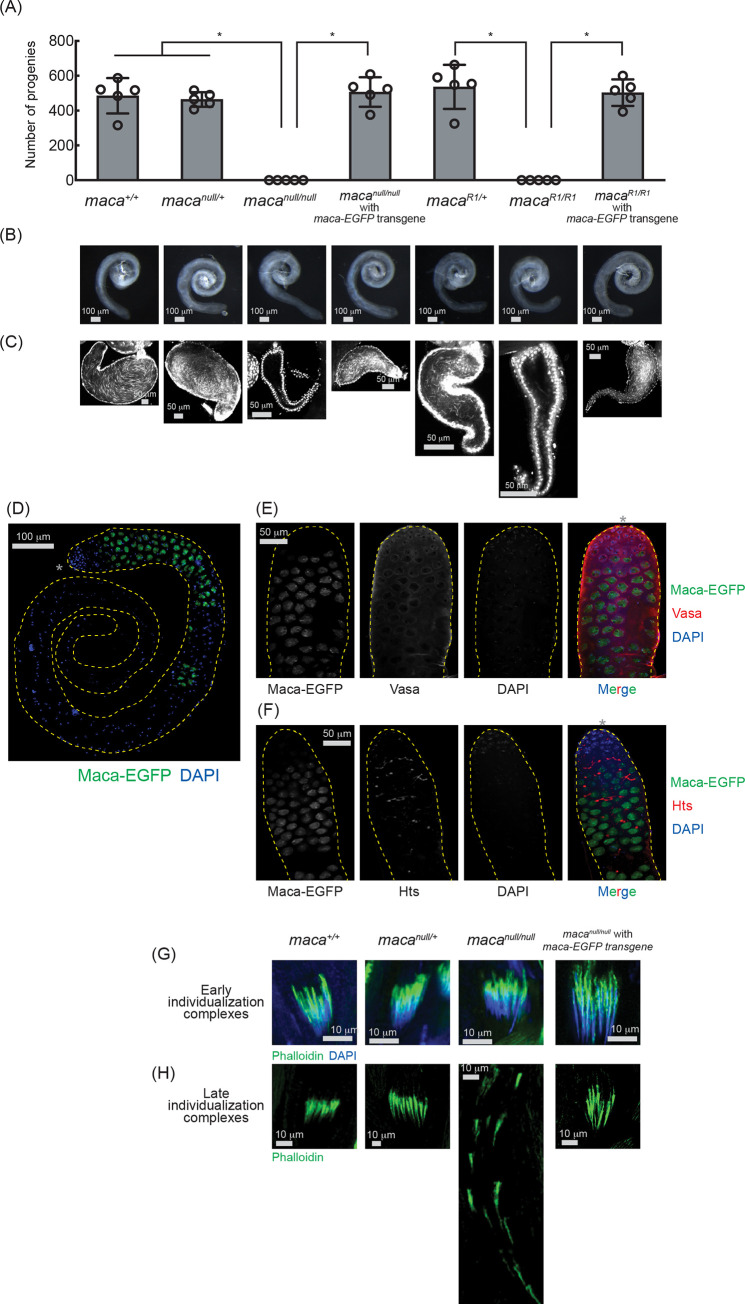
*maca* mutant flies are male-sterile, lack mature motile sperm, and have disorganized individualization complexes. (A) Male fertility assay. The numbers of the progeny flies obtained from the crosses between test males and OregonR wild-type virgin females are shown. Mean +/- SD (n = 5 biological replicates). P-value <0.05 (Student’s t-test, unpaired, two-tailed) are indicated by *. (B) Stereomicroscope images of dissected whole testes. Scale bars are 100 μm. (C) Confocal images of dissected seminal vesicles stained with DAPI. Mature motile sperm is absent in *maca*^*null/null*^ and *maca*^*R1/R1*^. Scale bars are 50 μm. (D) Confocal images of dissected whole testis from the *maca-EGFP* transgenic fly. Maca-EGFP (green) and DAPI (blue). The apical tip of the testis, where the hub cells reside, is marked with an asterisk (*). Scale bar is 100 μm. (E) Confocal images of the apical region of the testis from the *maca-EGFP* transgenic fly. Maca-EGFP (green), Vasa (red), and DAPI (blue). The apical tip of the testis, where the hub cells reside, is marked with an asterisk (*) in the merged panel. Scale bar is 100 μm. (F) Confocal images of the apical region of the testis from the *maca-EGFP* transgenic fly. Maca-EGFP (green), Hts (red), and DAPI (blue). The apical tip of the testis, where the hub cells reside, is marked with an asterisk (*) in the merged panel. Scale bar is 100 μm. (G) Confocal images of the early-stage individualization complexes. Phalloidin (Actin, green), DAPI (blue). Scale bars are 10 μm. (H) Confocal images of the late-stage individualization complexes. Phalloidin (Actin, green). Scale bars are 10 μm.

Maca’s testis-specific expression (Fig 1D and 1E) suggested that Maca plays an important role in testes. To test this, we performed fertility assays to determine whether *maca* is required for male fertility. Hundreds of progenies were obtained when the control flies (*maca*^*+l+*^, *maca*^*null/+*^, and *maca*^*R1/+*^) were crossed with wild-type virgin females (Fig 2A). In contrast, no progenies were obtained at all when *maca*^*null/null*^ or *maca*^*R1/R1*^ male flies were crossed with wild-type virgin females. Trans-heterozygous mutant flies *maca*^*null/Df*^ and *maca*^*R1/Df*^, which have the *maca*^*null*^ or *maca*^*R1*^ allele and the *Df(3R)Exel6174* chromosomal deficiency allele uncovering the *maca* gene, also showed complete male sterility (S1A Fig). These results indicated that *maca* is strictly required for male fertility. To confirm that male sterility is due to loss of *maca*, not due to any unintended secondary mutations, we tested if a *maca* transgene can rescue male fertility in the *maca*^*null/null*^ and *maca*^*R1/R1*^ backgrounds. We created *maca* transgenic rescue fly strains using a *maca* transgene that expresses C-terminally EGFP-fused Maca protein under control of a *maca* promoter. This *maca-EGFP* transgenic allele fully rescued male fertility in the *maca*^*null/null*^ and *maca*^*R1/R1*^ backgrounds (Fig 2A). *maca*^*null/null*^, *maca*^*null/Df*^, *maca*^*R1/R1*^, and *maca*^*R1/Df*^ did not exhibit any significant changes in female fertility compared with the controls (S1B and S1C Fig). We concluded that *maca* is strictly required for male fertility, but not for female fertility. This is consistent with the observation that Maca is expressed almost exclusively in testes but not in female flies (Fig 1D and 1E).

### *maca* is essential for spermatogenesis

To explore the mechanism underlying the complete male sterility in the *maca* mutant flies (Figs 2A and S1), we first examined whole testis morphology. We did not observe any major defects in whole testis morphology in the *maca* mutant flies compared with the controls (Fig 2B). Next, using DAPI staining of needle-shaped sperm nucleus and confocal imaging, we examined mature motile sperm, which are produced in testes and stored in seminal vesicles. As expected, we observed plenty of mature motile sperm in the seminal vesicles of the control flies (*maca*^*+l+*^, *maca*^*null/+*^, and *maca*^*R1/+*^) (Fig 2C). In contrast, the seminal vesicles of *maca*^*null/null*^ and *maca*^*R1/R1*^ flies were empty lacking mature motile sperm. Plenty of mature motile sperm were observed in the seminal vesicles of *maca*^*null/null*^ and *maca*^*R1/R1*^ flies with the *maca-EGFP* rescue transgene. Using the *DJ-GFP* allele, which labels mature motile sperm with bright GFP signal in the control seminal vesicles, we further confirmed the loss of mature motile sperm in the seminal vesicles of *maca*^*null/null*^ and *maca*^*R1/R1*^ (S2 Fig). We concluded that *maca* is essential for spermatogenesis.

### Maca is expressed in spermatocytes and resides in the nucleus

We investigated an expression pattern of Maca protein in testes using the *maca-*EGFP transgenic flies. We found that Maca-EGFP is expressed in spermatocytes residing in the nucleus and becomes undetectable after spermatocytes complete their growth (Fig 2D). Next, we performed immunostaining of *maca-*EGFP transgenic fly testes with Vasa and Hu-li tai shao (Hts) antibodies. Maca-EGFP was expressed in spermatocytes and was localized in the nucleus while Vasa was expressed in germline stem cells and developing germline cells including spermatogonia and spermatocytes and resided in the cytoplasm and at the nuclear periphery (Fig 2E). Hts labels filamentous fusome structures connecting the dividing spermatogonia and spermatocytes (Fig 2F). Maca-EGFP started to be expressed soon after the Hts filamentous structures were formed. These results together showed that Maca-EGFP is expressed in spermatocytes and resides in the nucleus. We also transiently expressed EGFP-Maca in cultured S2 cells and found that EGFP-Maca protein is localized in the S2 cell nucleus (S3 Fig), confirming that Maca is a nuclear protein.

### *maca* is essential for sperm individualization

To examine which step in spermatogenesis is impaired in *maca*^*null/null*^ testes, we examined the sperm individualization process [37]. Individualization complexes were properly organized in the early stage of the sperm individualization process in *maca*^*null/null*^ testes as in the control testes (Fig 2G). However, unlike in the control testes, individualization complexes were disorganized and scattered in the late stage of the individualization process in *maca*^*null/null*^ testes, showing axoneme formation defects (Fig 2H). Proper individualization complex organization was rescued by the transgenic *maca-EGFP* in the *maca*^*null/null*^ background. We concluded that *maca* is required for proper individualization complex organization in the late stage of the sperm individualization process. Loss of proper sperm individualization explains the loss of mature sperm and the complete male sterility in *maca*^*null/null*^.

### Kl-2, Kl-3, and Kl-5 protein levels are reduced in *maca*^*null/null*^ testes

**Fig 3:**
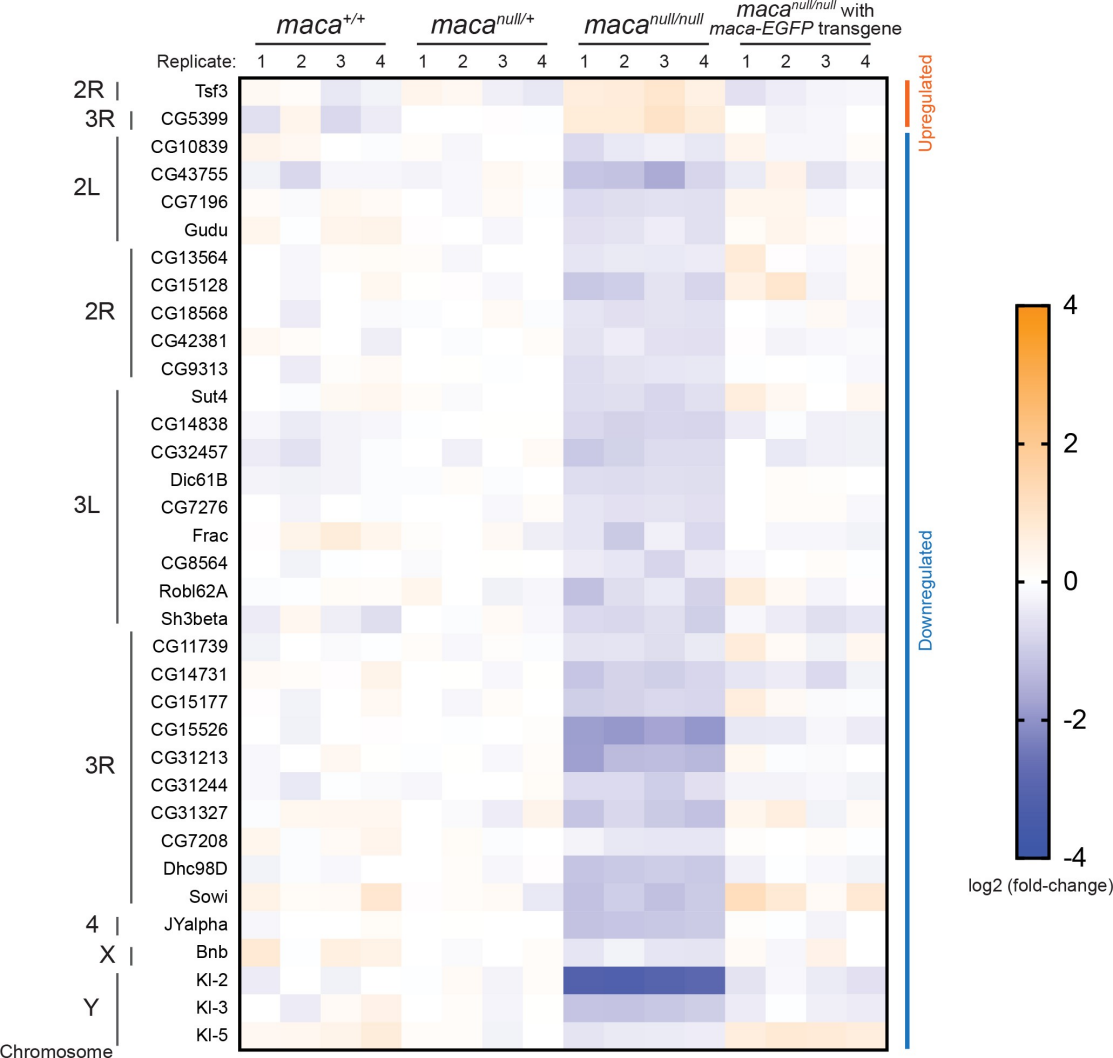
Proteins dysregulated in *maca*^*null/null*^ testes including downregulated Kl-2, Kl-3, and Kl-5 revealed by mass spec. Heatmap of protein levels that were significantly dysregulated in the *maca*^*null/null*^ testes compared with the testes from *maca*^*+/+*^, *maca*^*null/+*^, and *maca*^*null/null*^ with *maca-EGFP* rescue transgene determined by mass spec with TMT labeling. Log_2_(fold-change compared with the means of *maca*^*null/+*^) are shown. Four biological replicates for each genotype. Two proteins (at the top) were significantly upregulated (relative abundance >1.3, adjusted P-value <0.05) and 33 proteins were significantly downregulated (relative abundance <0.77, adjusted P-value <0.05) in *maca*^*null/null*^ compared with all the other three genotypes. Male fertility factor Kl-2 was most strongly and significantly downregulated (relative abundance 0.12 and adjusted P-value 1.7 x 10^−5^ in *maca*^*null/null*^ compared with *maca*^*null/+*^). Proteins are sorted based on the chromosomal positions of the corresponding genes.

To start to understand the molecular mechanism by which the sperm individualization complexes are impaired in *maca*^*null/null*^ testes, we examined protein levels in a proteome-wide manner in four biological replicates each of the testes of *maca*^*+/+*^, *maca*^*null/+*^, *maca*^*null/null*^, and *maca*^*null/null*^ with *maca-EGFP* rescue transgene, using mass spec with TMT-labeling (see Materials and Methods). Among the 6,559 proteins we detected, two proteins were significantly upregulated (relative abundance >1.3, adjusted P-value <0.05) and 33 proteins were significantly downregulated (relative abundance <0.77, adjusted P-value <0.05) in *maca*^*null/null*^ compared with all the other three genotypes consistently (Figs 3 and S4A). Among these consistently dysregulated proteins, male fertility factor Kl-2 showed the strongest and most significant downregulation in *maca*^*null/null*^ (relative abundance 0.12 and adjusted P-value 1.7 x 10^−5^ in *maca*^*null/null*^ compared with *maca*^*null/+*^) (Figs 3 and S4A). Interestingly, two other male fertility factors Kl-3 and Kl-5 were also among the 33 consistently downregulated proteins. Kl-2 is an inner dynein arm (IDA) dynein heavy chain protein and Kl-3 and Kl-5 are outer dynein arm (ODA) dynein heavy chain proteins [23–28]. The male fertility factor genes *kl-2*, *kl-3*, and *kl-5* encoding these proteins are all located in the Y chromosome long arm and span several megabases containing gigantic introns rich in repetitive sequences (Fig 1B).

### No significant change in small RNA levels in *maca*^*null/null*^ testes

Next, we examined testis small RNAs (miRNAs, siRNA, and piRNAs) from three biological replicates each of *maca*^*+/+*^, *maca*^*null/+*^, *maca*^*null/null*^, *maca*^*null/+*^ with *maca-EGFP* rescue transgene, and *maca*^*null/null*^ with *maca-EGFP* rescue transgene in a transcriptome-wide manner using high throughput sequencing. No miRNAs, siRNA, or piRNAs (including Y-linked *Suppressor of Stellate* (*Su(Ste*) piRNAs, which are crucial for male fertility[38]) showed significantly changed levels in *maca*^*null/null*^ testes compared with the other four genotypes (S5 Fig).

### *kl-2*, *kl-3*, and *kl-5* mRNA levels are reduced in *maca*^*null/null*^ testes

**Fig 4:**
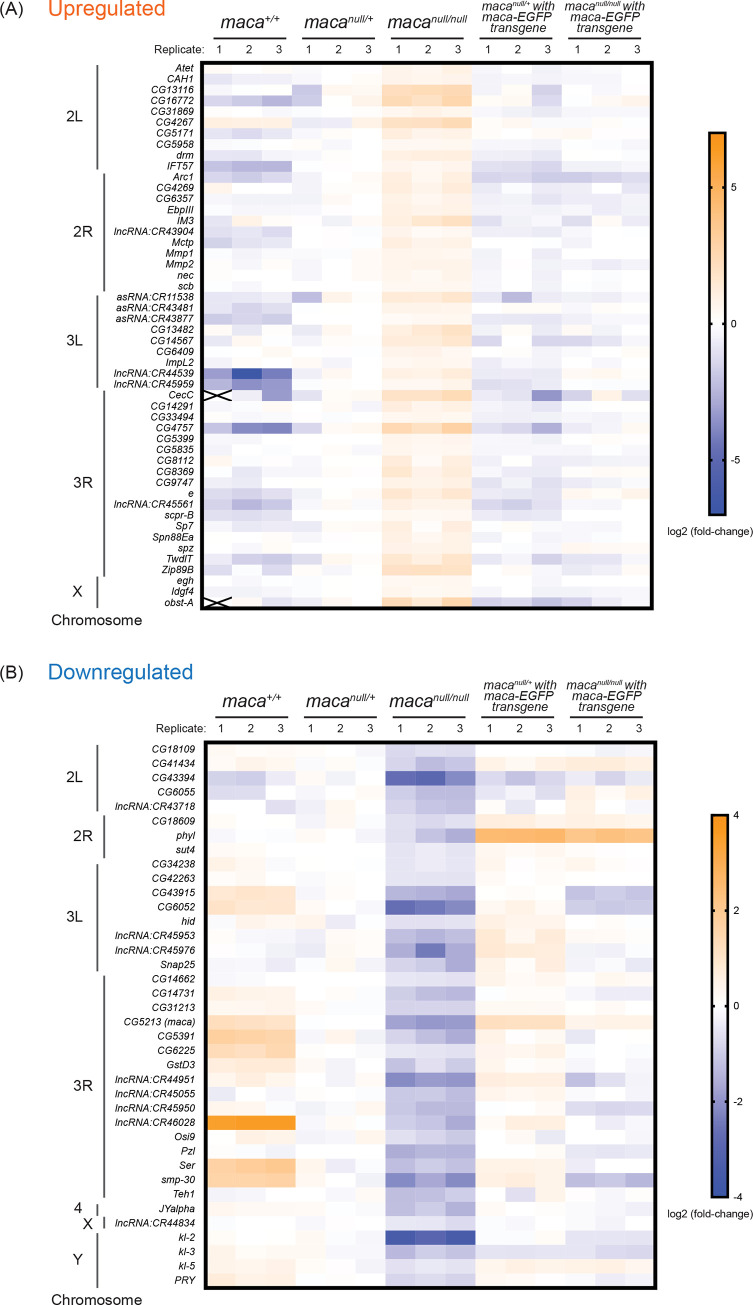
Poly-A+ RNA-seq analysis revealed RNAs dysregulated in *maca*^*null/null*^ testes including downregulated *kl-2*, *kl-3*, and *kl-5* mRNAs. (A, B) Heatmaps of RNA levels that are significantly upregulated or downregulated in the *maca*^*null/null*^ testes compared with the testes from *maca*^*+/+*^, *maca*^*null/+*^, *maca*^*null/+*^ with *maca-EGFP* rescue transgene, and *maca*^*null/null*^ with *maca-EGFP* rescue transgene, determined by poly-A+ RNA-seq. Log_2_(fold-change compared with the means of *maca*^*null/+*^) are shown. Three biological replicates for each genotype. (A) Fifty genes were significantly upregulated (relative abundance >1.32, adjusted P-value <0.01) and (B) 38 genes were significantly downregulated (relative abundance <0.76, adjusted P-value <0.01) in *maca*^*null/null*^ compared with all the other four genotypes. Male fertility factor *kl-2* mRNA was most strongly and significantly downregulated (relative abundance 0.10 and adjusted P-value 1.65 x 10^−196^ in *maca*^*null/null*^ compared with *maca*^*null/+*^). Genes are sorted based on their chromosomal positions.

**Fig 5:**
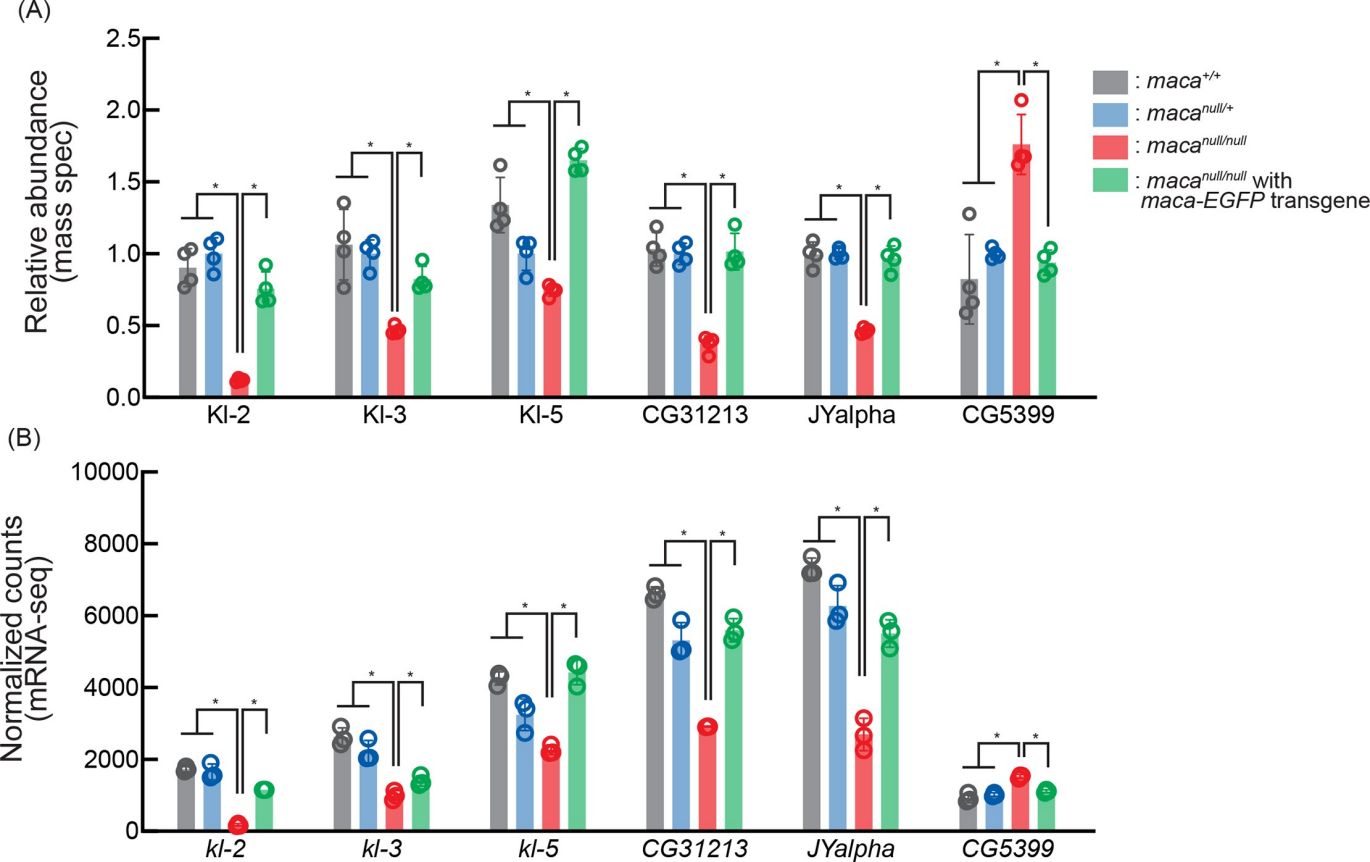
Abundance of both protein and mRNA of *kl-2*, *kl-3*, and *kl-5* are decreased in *maca*^*null/null*^ testes. (A) Relative abundance of dysregulated proteins determined by mass spec whose mRNAs were also dysregulated in *maca*^*null/null*^ compared with all the other tested genotypes. Mean +/- SD (n = 4 biological replicates). Adjusted P-values <0.05 are indicated by *. (B) Normalized counts (reflecting relative abundance) of dysregulated mRNAs determined by poly-A+ RNA-seq whose protein products were also dysregulated in *maca*^*null/null*^ compared with all the other tested genotypes. Mean +/- SD (n = 3 biological replicates). Adjusted P-values <0.01 are indicated by *.

**Fig 6:**
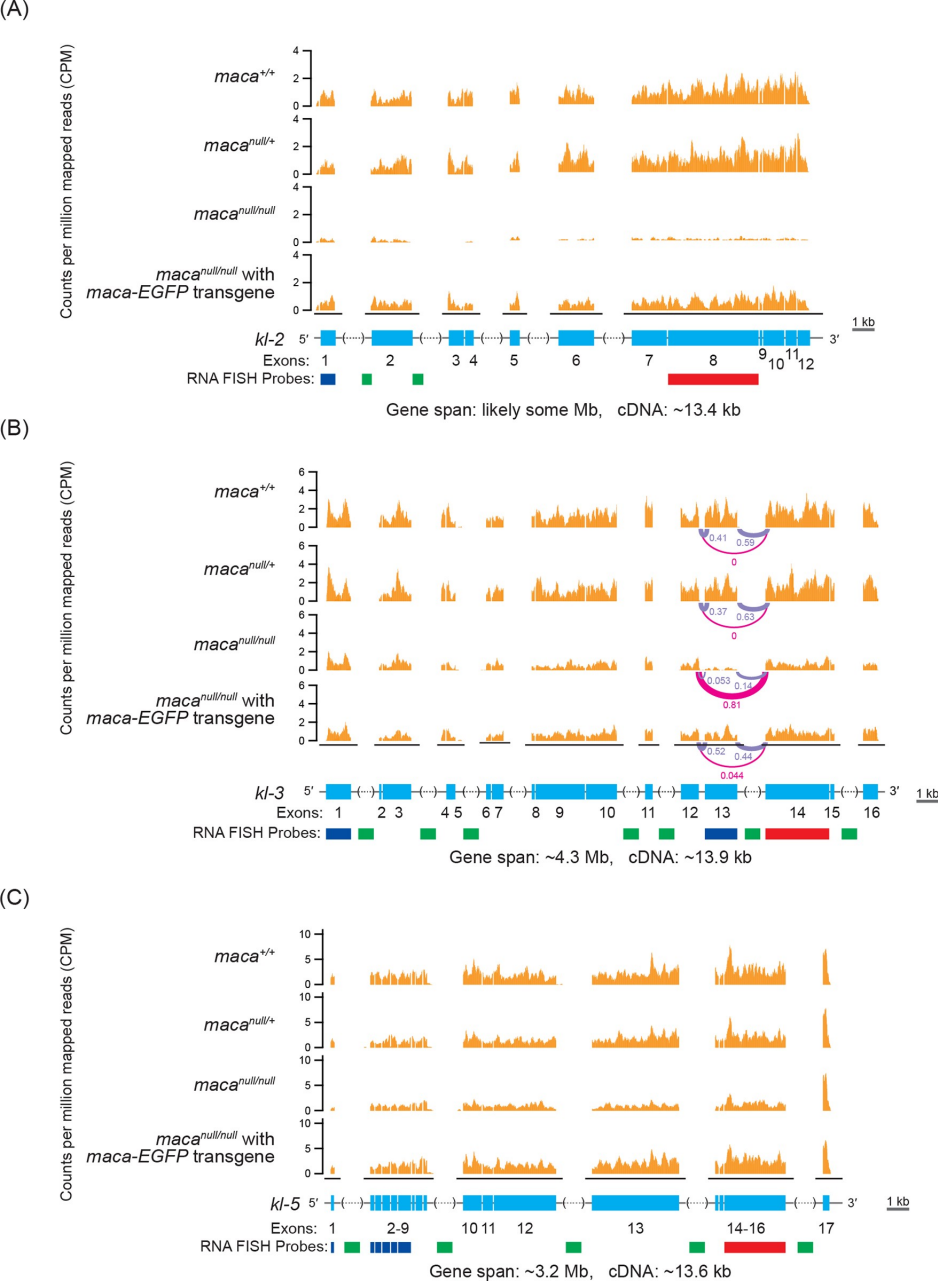
Poly-A+ RNA-seq read mapping counts for *kl-2*, *kl-3*, and *kl-5* mRNAs show their reduction in *maca*^*null/null*^ testes. Poly-A+ RNA-seq normalized read counts (counts per million mapped reads, CPM) in the (A) *kl-2*, (B) *kl-3*, and (C) *kl-5* gene regions. For *kl-3*, splicing ratios among (1) exon 12—exon 13, (2) exon 13—exon 14, and (3) exon 12—exon 14 (= exon 13-skipped) determined by LeafCutter [39] are shown. Only one replicate out of 3 is shown for each genotype. All replicates are shown in S5–S7 Figs. Gene structures are shown with exons (cyan), introns (black line), intronic satellite DNA repeats (dashed line in parentheses. Contain gaps in the publically available *Drosophila* genome sequence). Regions targeted by RNA FISH probes used in Figs 8, 10, S10, S13, S14, S15 and S16 (blue, green, and red).

Considering that Maca resides in the nucleus (Figs 2D, 2E and S3) and has RNA-recognition motifs, if any observed protein level dysregulation in *maca*^*null/null*^ testes is directly caused by *maca* loss of function, then it is likely through dysregulation of their corresponding transcripts rather than translational or post-translational dysregulation. Therefore, we examined testis poly-A+ RNAs in three biological replicates each of *maca*^*+/+*^, *maca*^*null/+*^, *maca*^*null/null*^, *maca*^*null/+*^ with *maca-EGFP* transgene, and *maca*^*null/null*^ with *maca-EGFP* transgene in a transcriptome-wide manner using high throughput sequencing (poly-A+ RNA-seq). Among the 17,137 genes we examined, we detected reads for 15,937 genes in at least one sample. Among them, 50 genes showed significant upregulation (relative abundance >1.32, adjusted P-value <0.01) and 38 genes showed significant downregulation (relative abundance <0.76, adjusted P-value <0.01) in mRNA levels in *maca*^*null/null*^ testes compared with all the other four genotypes consistently (Fig 4A and 4B). Interestingly, male fertility factor *kl-2* mRNA showed the most significant downregulation in *maca*^*null/null*^ testes among all the detected mRNAs (relative abundance 0.10 and adjusted P-value 1.65 x 10^−196^ in *maca*^*null/null*^ compared with *maca*^*null/+*^) (Figs 4B, 6A, S4B and S5). Moreover, *kl-3* and *kl-5* mRNAs were also among the 38 consistently downregulated (Figs 4B, 6B, 6C, S4B, S7 and S8). Thus, *kl-2*, *kl-3*, and *kl-5* exhibited significant downregulation in both protein and mRNA levels in *maca*^*null/null*^ testes compared with all the other genotypes (Fig 5). *CG31213* and *JYalpha* also showed both protein and mRNA level downregulation and *CG5399* showed upregulation in *maca*^*null/null*^ testes compared with the all other genotypes (Figs 5 and S4). *kl-2*, *kl-3*, and *kl-5* are known to be essential for male fertility [27,29,39] and thus their downregulation in *maca*^*null/null*^ testes can nicely explain the male sterility in *maca*^*null/null*^. Therefore, we decided to focus on *kl-2*, *kl-3*, and *kl-5*.

### *kl-3* exon 13 skipping occurs in *maca*^*null/null*^ testes

We wondered if splicing dysregulation occurs in any transcripts in *maca*^*null/null*^ testes. To test this, we analyzed our poly-A+ RNA-seq data using LeafCutter [39]. Curiously, we found that *kl-3* mRNA exon 13 skipping occurs in *maca*^*null/null*^ testes, but not in the other four genotypes (Figs 6B and S7). No other mRNA showed consistent splicing dysregulation in *maca*^*null/null*^ compared with the other four genotypes. We also found that read mapping count was reduced rather evenly along the *kl-2* and *kl-5* gene regions in *maca*^*null/null*^ compared with the other genotypes (Figs 6A, 6C, S6 and S8). In contrast, read mapping was reduced more severely in the *kl-3* exon 13 region than in the other *kl-3* coding regions in *maca*^*null/null*^, which is consistent with the exon 13 skipping (Figs 6B and S7). Thus, our poly-A+ RNA-seq data revealed *kl-3* exon 13 skipping in *maca*^*null/null*^ testes. No major mRNA level reduction, exon skipping, or protein level reduction for *ks-1* (*ory*) was observed in *maca*^*null/null*^ compared with the controls (S9 Fig).

### *maca* mutation phenocopies *kl-2*, *kl-3*, and *kl-5* RNAi knockdown

Gene mutation or RNAi-mediated knockdown of *kl-2*, *kl-3*, or *kl-5* in testis germline cells causes spermatogenesis defects including a scattering of the individualization complexes and male sterility [27,29,39]. We first confirmed that *kl-2*, *kl-3*, or *kl-5* RNAi knockdown depleted corresponding cytoplasmic mature mRNAs, but not the nuclear precursor mRNA (pre-mRNA) transcripts in spermatocytes (S10 Fig). *kl-2*, *kl-3*, or *kl-5* RNAi knockdown caused loss of mature motile sperm in seminal vesicles (S11A Fig) and scattering of the individualization complexes in the late stage of the sperm individualization process (S11B and S11C Fig), similar to *maca* mutation (Fig 2C, 2G and 2H). Thus, *maca* mutation and *kl-2*, *kl-3*, or *kl-5* RNAi knockdown depleting their cytoplasmic mRNAs phenocopy each other.

### *kl-2* transcript level reduction in *maca*^*null/null*^ testes detected with RT-qPCR

**Fig 7:**
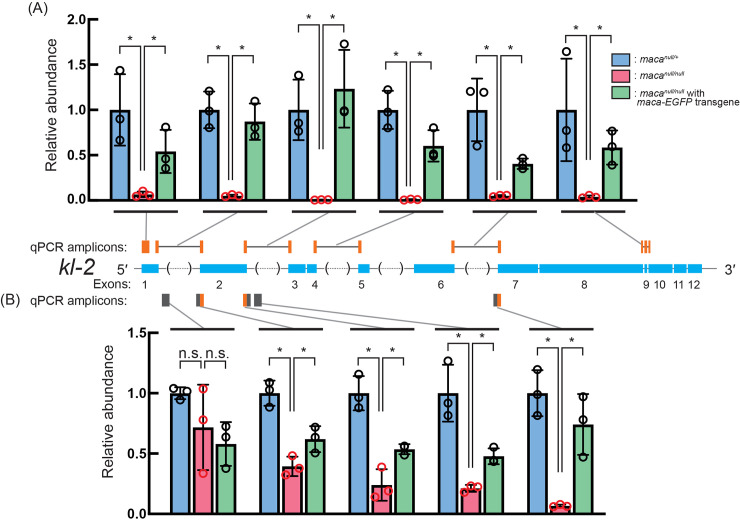
Quantification of *kl-2* transcripts by RT-qPCR show their reduction in *maca*^*null/null*^ testes. Quantification of *kl-2* transcripts in testis RNA by RT-qPCR. The RT step was performed with random hexamer primers. (A) qPCR amplicons targeted by designed primer sets are indicated by orange bars. The primers span exon-exon junctions to amplify only spliced transcripts, except for the first amplicon, which targets exon 1. Black lines indicate intron regions not included in the amplicons. (B) qPCR amplicons targeted by designed primer sets are indicated by gray and orange bars, which indicate intron and exons regions, respectively. At least one primer in each primer set targets an intron and therefore the primer sets target *kl-2* transcripts before splicing (pre-mRNAs). Data were normalized to *actin5C* mRNA and the means of *maca*^*null/+*^. Mean +/- SD (n = 3 biological replicates). P-values <0.05 (Student’s t-test, unpaired, two-tailed) are indicated by *.

To validate the strong *kl-2* mRNA level reduction observed in poly-A+ RNA-seq (Figs 4B, 5B, S4B and S6), we quantitated *kl-2* transcripts in testis using RT-qPCR. We performed the reverse transcription (RT) step using random primers so that precursor transcripts including transcription intermediates before poly-A addition as well as mature poly-A+ mRNAs can be detected in the RT-qPCR assays. When qPCR primer set that amplifies a region in *kl-2* exon 1 was used, *maca*^*null/null*^ showed a dramatic reduction compared with the control *maca*^*null/+*^ (became 0.07 fold) (Fig 7A). Similarly, when qPCR primer sets spanning large *kl-2* introns and that spanning normal size *kl-2* introns were used, *maca*^*null/null*^ showed a severe reduction (became 0.01–0.05 fold). Mature *kl-2* mRNAs can be detected by all of these primer sets.

Next, we used qPCR primer sets to amplify regions in *kl-2* introns and regions located at *kl-2* exon-intron or intron-exon boundaries. *kl-2* pre-mRNA transcripts before splicing, but not mature *kl-2* mRNAs after splicing, can be detected by these qPCR primer sets. With these primer sets, the reduction degree in *maca*^*null/null*^ compared with the control became gradually stronger as a primer set target location is shifted from the 5′ end to the 3′ end of the *kl-2* gene (Fig 7B). Yet, the reduction degrees in *maca*^*null/null*^ detected using these primer sets targeting *kl-2* pre-mRNAs (Fig 7B) were not as strong as those obtained using the primer sets targeting the *kl-2* exon regions (Fig 7A). The *kl-2* transcript level was rescued in *maca*^*null/null*^ with *maca-EGFP* rescue transgene in most cases (Fig 7A and 7B). Thus, in *maca*^*null/null*^ testes, the *kl-2* pre-mRNA transcript levels are more strongly reduced toward the 3′ end and the mature *kl-2* mRNA levels are dramatically reduced, suggesting that later steps in the *kl-2* mRNA transcription/processing/stability is more strongly affected than early steps.

### Severe reduction of cytoplasmic mature *kl-2* mRNA in *maca*^*null/null*^ spermatocytes revealed by RNA FISH

**Fig 8:**
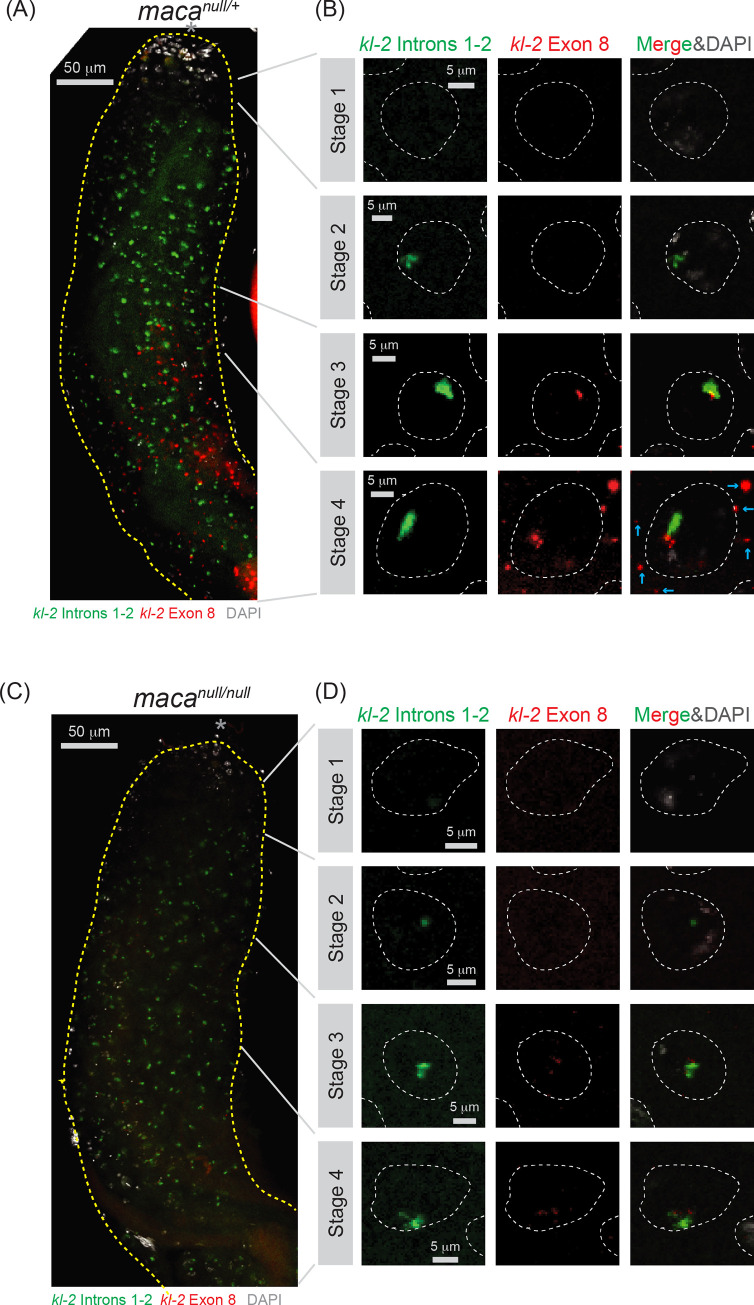
RNA FISH for *kl-2* transcripts shows their reduction in *maca*^*null/null*^ testes. RNA FISH to visualize *kl-2* transcripts expression in (A, B) *maca*^*null/+*^ and (C, D) *maca*^*null/null*^ testes. Introns 1–2 (green), exon 8 (red), and DAPI (white). (A, C) Apical regions of testes including the spermatocyte growth region are shown. The apical tip of testis is marked by *. Scale bars are 50 μm. (B, D) Single spermatocyte nuclei (white dashed line) at each stage of *kl-2*. Cytoplasmic mRNA granules are indicated by cyan arrows. Scale bars are 5 μm.

Next, we performed RNA in-situ hybridization (FISH) to visualize a spatiotemporal expression pattern of *kl-2* transcripts in testes. In the control *maca*^*null/+*^ testes, we found that transcription of the *kl-2* gene progresses in a highly spatiotemporal manner as previously shown for the *kl-3* and *kl-5* genes [40]. *kl-3* and *kl-5* expression during spermatocyte growth was subdivided into four stages based on their transcription progress from the 5′ end to the 3′ end of the genes [40]. Here we subdivided *kl-2* expression in four stages as well. The transcription initiation occurs in early spermatocytes where neither *kl-2* introns 1–2 nor exon 8 was detectable yet in the nucleus (stage 1) (Fig 8A and 8B). In the next stage, *kl-2* introns 1–2 were expressed (stage 2). Then, *kl-2* exon 8 was additionally expressed (stage 3). Finally, while the intron and exon signals were continued to be detected in the nucleus, mature *kl-2* mRNAs that contain only exons, but not introns, were exported to the cytoplasm and reside as cytoplasmic kl-granules in late spermatocytes (stage 4). Thus, the gigantic *kl-2* gene is transcribed gradually during the ~90-hour spermatocyte growth, and only in the late stage, *kl-2* gene transcription is completed and spliced mature *kl-2* mRNAs are exported to the cytoplasm.

In *maca*^*null/null*^ testes, FISH signal for *kl-2* pre-mRNA transcripts in the spermatocyte nucleus was weaker than in *maca*^*null/+*^ testes (Fig 8C and 8D). FISH signal for *kl-2* exon 8 seemed more severely reduced than those for *kl-2* introns 1–2. Furthermore, cytoplasmic mature *kl-2* mRNA granule in stage 4 spermatocyte was almost completely absent in *maca*^*null/null*^ testes. We concluded that *kl-2* pre-mRNA transcript levels are detectably and cytoplasmic *kl-2* mature mRNA levels are severely reduced in *maca*^*null/null*^ testes, consistent with our RT-qPCR results (Fig 7).

### *kl-3* mRNA reduction and *kl-3* exon 13 skipping in *maca*^*null/null*^ testes confirmed by RT-PCR

**Fig 9:**
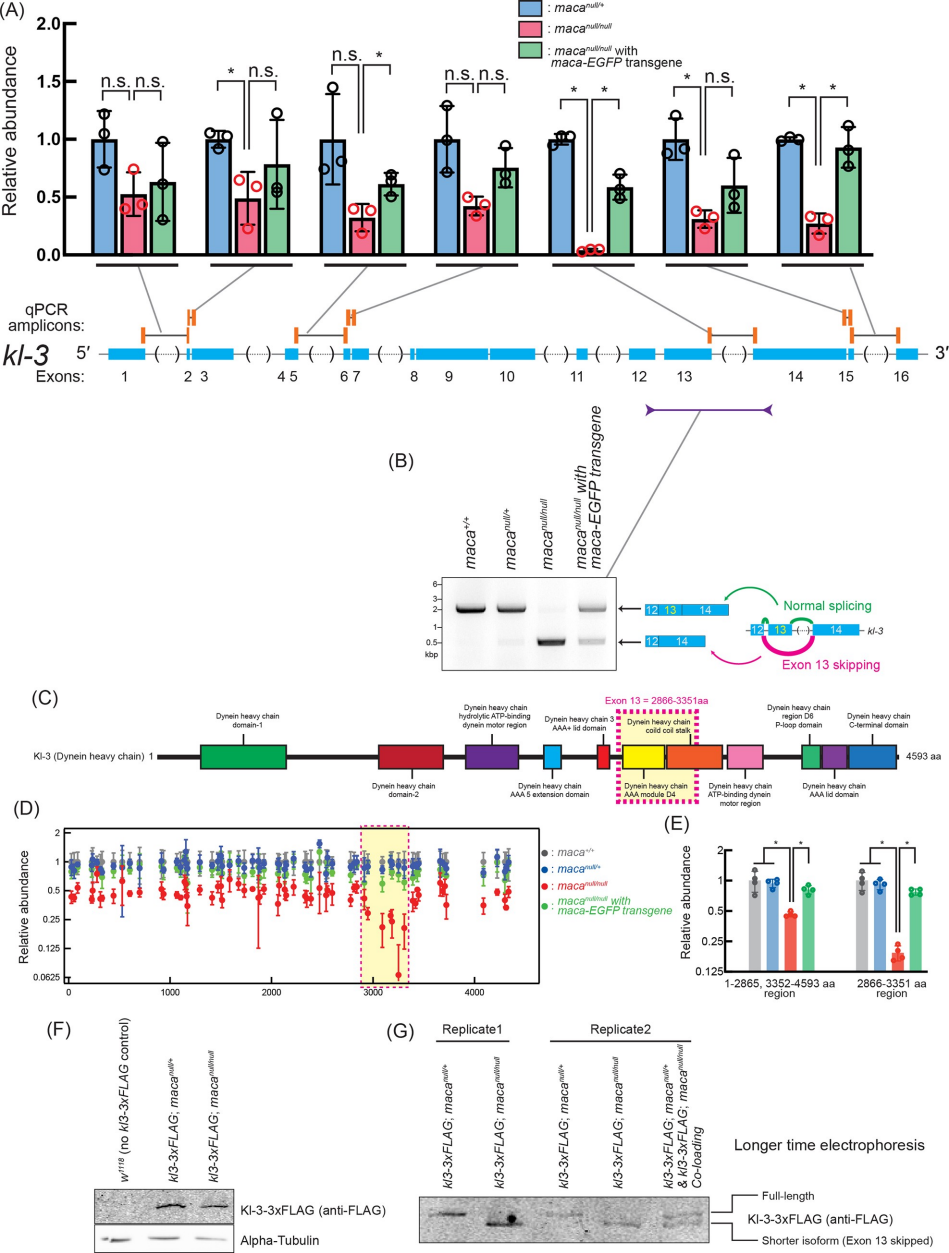
*kl-3* mRNA reduction and exon 13 skipping in *maca*^*null/null*^ testes revealed by RT-(q)PCR. (A) Quantification of *kl-3* transcripts in testis RNA by RT-qPCR. The RT step was performed with random hexamer primers. qPCR amplicons targeted by designed primer sets are indicated by orange bars. The primers span exon-exon junctions to amplify only spliced transcripts. Black lines indicate intron regions not included in the amplicons. Data were normalized to *actin5C* mRNA and the mean of *maca*^*null/+*^. Mean +/- SD (n = 3 biological replicates). P-values <0.05 (Student’s t-test, unpaired, two-tailed) are indicated by *. (B) Agarose gel electrophoresis of RT-PCR products using the indicated primers to examine *kl-3* exon 13 skipping in testis RNA. (C) Diagram of Kl-3 protein domains. Amino acid residues 2866–3351 corresponding to the exon 13 is indicated with a magenta dash-lined yellow box. (D) Abundance of peptide fragments derived from Kl-3 protein determined by mass spec with TMT labeling plotted along the Kl-3 amino acid residue numbers. Data were normalized to the means of *maca*^*+/+*^. Mean +/- SD (n = 4 biological replicates). The amino acid residues 2866–3351 corresponding to the *kl-3* mRNA exon 13 is indicated with a magenta dash-lined yellow box. (E) Abundance of Kl-3 protein determined using peptide fragments data (left) from the 1–2865 or 3352–4593 aa region or (right) from the 2866–3351 aa region, of Kl-3 in mass spec with TMT labeling. Data were normalized to the means of *maca*^*+/+*^. Mean +/- SD (n = 4 biological replicates). P-values <0.05 (Student’s t-test, unpaired, two-tailed) are indicated by *. (F, G) Western blots of testis lysates for Kl-3-3xFLAG using (F) a standard protocol and (G) an extended SDS-PAGE gel electrophoresis time to achieve better separation. The FLAG tag was inserted at the C-terminal end of Kl-3 at the endogenous *kl-3* locus on the Y-chromosome [40].

To validate the *kl-3* mRNA level reduction and the *kl-3* exon 13 skipping observed in the poly-A+ RNA-seq analysis (Figs 4B, 5B, 6B, S4B and S7), we performed RT-qPCR to measure *kl-3* mRNA levels in testes. Primer sets spanning *kl-3* introns to amplify mature *kl-3* mRNAs revealed a reduction to 0.27–0.53 fold in *maca*^*null/null*^ testes compared with *maca*^*null/+*^, except for one primer set (Fig 9A). The exception was the qPCR primer set with a forward primer targeting a region in exon 13 and a reverse primer targeting the junction between exon 13 and exon 14 (= spanning intron 13); it showed a much stronger reduction in *maca*^*null/null*^ testes compared with *maca*^*null/+*^ (became 0.04 fold). The *kl-3* mRNA levels were rescued in *maca*^*null/null*^ with *maca-EGFP* rescue transgene. These results confirm the *kl-3* mRNA level reduction and the *kl-3* exon 13 skipping, consistent with the poly-A+ RNA-seq results.

To further confirm the *kl-3* exon 13 skipping, we performed RT-PCR using testis RNA with a forward PCR primer targeting a region in exon 12 and a reverse PCR primer targeting a region in exon 14 and ran the PCR products on an agarose gel. A ~2.1 kb band showing exon 13 retention was predominantly obtained from the testis RNA of the controls (*maca*^*+/+*^ and *maca*^*null/+*^) and *maca*^*null/null*^ with *maca-EGFP* rescue transgene, while a ~0.6 kb band showing exon 13 skipping was predominantly obtained from the testis RNA of *maca*^*null/null*^ (Fig 9B). These results confirmed the *kl-3* exon 13 skipping in *maca*^*null/null*^ testes.

Similarly, we detected the *kl-3* exon 13 skipping when whole adult male RNA was used for RT-PCR analysis (S12A Fig). The ~2.1 kb PCR product showing *kl-3* exon 13 retention was obtained from *maca*^*+/+*^ and *maca*^*null/+*^ males while the ~0.6 kb PCR product showing exon 13 skipping was obtained from *maca*^*null/null*^ males. Consistent with the exclusive expression of *maca* in testes (Fig 1D and 1E), no PCR bands were obtained when whole adult female RNA was used (S12A Fig). In contrast, sex-specific alternative splicing of *sxl*, *tra*, and *msl2*, was not dysregulated in *maca*^*null/null*^ males and females compared with *maca*^*+/+*^ and *maca*^*null/+*^ (S12B–S12D Fig). These results demonstrated that splicing dysregulation in *maca*^*null/null*^ is specific to *kl-3* exon 13 skipping, consistent with the poly-A+ RNA-seq analysis.

### *kl-3* exon 13 skipping causes an internal deletion of Kl-3 protein in *maca*^*null/null*^ testes

The *kl-3* mRNA exon 13 skipping would result in an internal deletion of amino acid residues 2,866–3,351 of Kl-3 protein without causing a translation frameshift (Fig 9C). We wondered if the internally deleted Kl-3 protein is expressed in *maca*^*null/null*^ testes. To test this, we first searched our TMT-labeled testis-proteome mass spec data for peptide fragments containing the 2,865–2,866 residues or the 3,351–3,352 residues of the full-length Kl-3 protein, which would derive from the full-length Kl-3 protein produced from *kl-3* mRNA with the exon 13-retained, or for peptide fragments containing the residue 2,865 followed by the residue 3,352, which would derive from the internally deleted Kl-3 protein variant produced from the exon 13-skipped mRNA. However, none of these fragments was detected in our data, which has ~20% coverage for Kl-3 protein.

Next, we took an alternative approach. The aa residues 1–2,865 and 3,352–4,593 are common between the full-length Kl-3 and the internally-deleted Kl-3 proteins while the aa residues 2,866–3,351 are specific to the full-length Kl-3. We analyzed the relative abundance of detected Kl-3 peptide fragments along the full-length Kl-3 amino acid sequence. Relative abundances of peptide fragment levels derived from the 2,866–3,351 amino acid residues were more strongly reduced in *maca*^*null/null*^ compared with the peptide fragment levels derived from the other Kl-3 region (Fig 9D). The Kl-3 protein level was calculated to be 0.46 fold in *maca*^*null/null*^ testes compared in *maca*^*+/+*^ when the peptide fragment data derived from the aa residues 1–2,865 and 3,352–4,593 were analyzed, while it was 0.19 fold when the peptide fragment data derived from the aa residues 2,866–3,351 were analyzed (Fig 9E). These mass spec data supported that *kl-3* exon 13 skipping causes an expression of the internally deleted Kl-3 protein variant in *maca*^*null/null*^ testes.

Furthermore, we attempted to detect the internally deleted Kl-3 protein variant using Western blot. We utilized a C-terminally 3xFLAG tagged *kl-3* gene at its endogenous locus in the Y-chromosome [40]. While the size difference of the C-terminally 3xFLAG tagged Kl-3 protein between in *maca*^*null/+*^ and in *maca*^*null/null*^ was not apparent in a standard protocol Western blot (Fig 9F), the size difference became apparent when gel electrophoresis time was much extended to achieve better separation (Fig 9G). The detected Kl-3 protein size was smaller in *maca*^*null/null*^ than in *maca*^*null/+*^, supporting the idea that the full-length protein is predominantly expressed in *maca*^*null/+*^ testes while the internally deleted Kl-3 protein is predominant in *maca*^*null/null*^ testes. When testis protein lysate from *maca*^*null/+*^ and that from *maca*^*null/null*^ were mixed and loaded together on a Western blot gel, it produced double bands, confirming the existence of two different size Kl-3 proteins (Fig 9G). Taken together, we concluded that the *kl-3* mRNA exon 13 skipping in *maca*^*null/null*^ testes causes expression of the Kl-3 protein variant with an internal deletion of the corresponding amino acid residues. The deleted region contains the entire Dynein heavy chain AAA module D4 domain and the N-terminal half of the Dynein heavy chain coiled coil stalk domain of the full-length Kl-3 (Fig 9C). The Kl-3 protein activity is likely to be significantly impaired by this deletion.

### *kl-3* exon 13 skipping in *maca*^*null/null*^ spermatocytes revealed by RNA FISH

We performed RNA FISH for *kl-3*. First, as shown previously [40], we observed gradual transcription of *kl-3* pre-mRNA transcripts from the 5′ end to the 3′ end during the spermatocyte growth using FISH probes against exon 1, introns, and exon 14, in the control *maca*^*null/+*^ testis (S13A and S13B Fig). As previously performed [40], to visualize *kl-3* introns, we probed the (AAUAU)_n_ repeat sequences, which are produced from the (AATAT)_n_ satellite DNA found in multiple *kl-3* introns including intron 1 (the intron between exon 1 and exon 2) (Fig 1B). (AATAT)_n_ is the only satellite DNA found in Y-loop B [41,42]. Only in late-stage spermatocytes, transcription of the entire *kl-3* gene is completed and cytoplasmic mature *kl-3* mRNA containing signal from both exon 1 and exon 14, but lacking signal from introns, were observed in kl-granules (S13B Fig, stage 4). Similarly, in *maca*^*null/null*^ also, we observed the gradual expression of the *kl-3* transcripts during stages 1–3 and cytoplasmic mature *kl-3* mRNA granules with overlapping signals from exon 1 and exon 14 lacking introns in stage 4 (S13C and S13D Fig).

**Fig 10:**
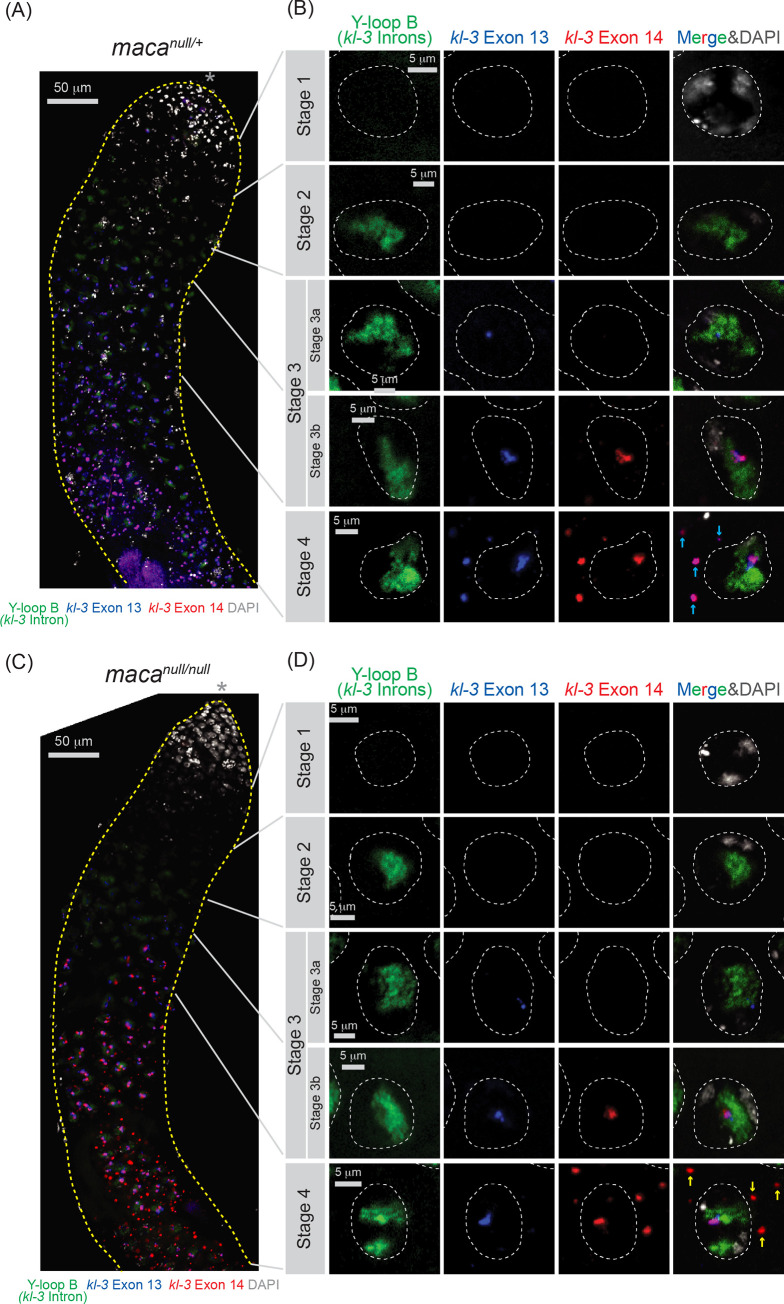
RNA FISH for *kl-3* transcripts shows exon 13 skipping in *maca*^*null/null*^ testes. RNA FISH to visualize *kl-3* transcripts in (A, B) *maca*^*null/+*^ and (C, D) *maca*^*null/null*^ testes. Introns (Y-loop B, Alexa488-(AATAT)_6_, green), exon 13 (blue), exon 14 (red), and DAPI (white). (A, C) Apical regions of testes including the spermatocyte growth region are shown. The apical tip of testis is marked by *. Scale bars are 50 μm. (B, D) Single spermatocyte nuclei (white dashed line) at each stage of *kl-3* expression. Cytoplasmic mRNA granules are indicated by cyan arrows for *maca*^*null/+*^, which contains signal from both exon 13 and exon 14, and by yellow arrows for *maca*^*null/null*^, which contains signal from exon 14 but not exon 13. Scale bars are 5 μm.

Next, to examine the exon 13 skipping, we used FISH probes for Y-loop B (*kl-3* introns), exon 13, and exon 14. Again, we observed gradual expression of the *kl-3* pre-mRNA transcripts. In stage 1 spermatocytes in the control *maca*^*null/+*^ testes, we did not observe any probe signal (Fig 10A and 10B). In stage 2, we observed only Y-loop B (*kl-3* introns) signal. Signal from Y-loop B (*kl-3* introns) and that from exon 13 were detected in the nucleus in stage 3a, while exon 14 signal was additionally observed in stage 3b. Finally, in stage 4, in addition to all these signals that were still observed in the nucleus, mature *kl-3* mRNA signal containing both exons 13 and 14, but lacking the Y-loop B (*kl-3* introns) signal, was observed in cytoplasm as kl-granules. In *maca*^*null/null*^, signal in stages 1–3 spermatocytes was similar to what was observed in *maca*^*null/+*^ (Fig 10C and 10D). In contrast, in the *maca*^*null/null*^ stage 4 spermatocytes, while both exon 13 and exon 14 signals were observed in the nucleus, cytoplasmic mRNA granules contained only exon 14 signal but not exon 13 signal (Fig 10C and 10D). These results together showed that cytoplasmic mature *kl-3* mRNAs in *maca*^*null/null*^ spermatocytes contain exons 1 and 14 but not exon 13, consistent with the exon 13 skipping. We note that because FISH is not a very quantitative assay, we did not reliably detect *kl-3* mRNA level reduction other than exon 13 region in *maca*^*null/null*^ spermatocytes, while our poly-A+ RNA-seq and qRT-PCR showed significant *kl-3* mRNA level reduction in *maca*^*null/null*^ testes.

### *kl-5* RT-qPCR and RNA FISH

**Fig 11:**
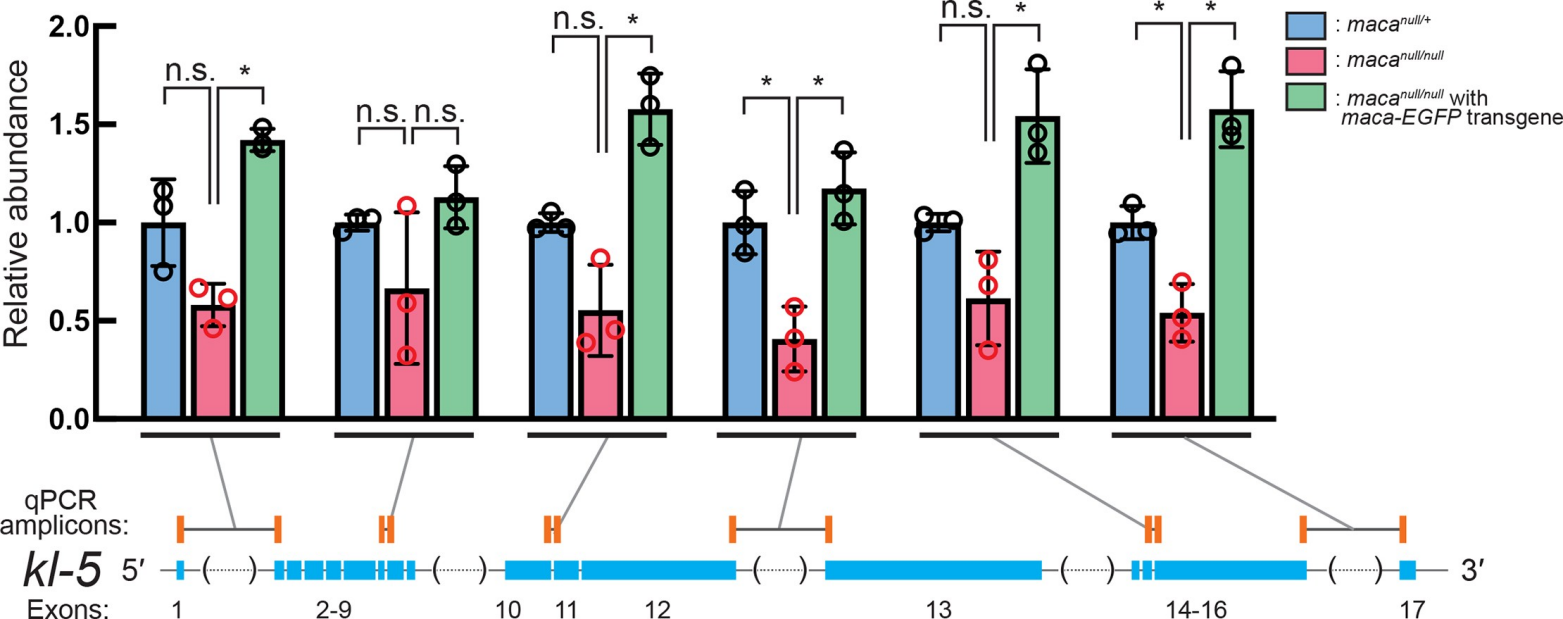
RT-qPCR of *kl-5* transcripts shows their reduction in *maca*^*null/null*^ testes. Quantification of *kl-5* transcripts in testis RNA by RT-qPCR. The RT step was performed with random hexamer primers. qPCR amplicons targeted by designed primer sets are indicated by orange bars. The primers span exon-exon junctions to amplify only spliced transcripts. Black lines indicate intron regions not included in the amplicons. Data were normalized to *actin5C* mRNA and the mean of *maca*^*null/+*^. Mean +/- SD (n = 3 biological replicates). P-values <0.05 (Student’s t-test, unpaired, two-tailed) are indicated by *.

We also performed RT-qPCR to measure *kl-5* mRNA levels and detected a moderate reduction in *maca*^*null/null*^ testes compared with the control testes using some of the primer sets tested (Fig 11). This is consistent with the statistically significant and consistent but rather mild reduction of *kl-5* mRNA level in *maca*^*null/null*^ testes revealed by poly-A+ RNA-seq (Figs 4B, 5B, 6C and S8). We also performed RNA FISH for *kl-5* with probes against exons 1–6, introns, and exons 16–17. As previously performed [40], for *kl-5* introns, we probed the (GUCUU)_n_ repeat sequences, which are produced from the (AAGAC)_n_ satellite DNA and found in Y-loops A and C (Fig 1B) [34]. Gradual transcription of *kl-5* gene during the spermatocytes growth and cytoplasmic mature *kl-5* mRNA with signal from both exons 1–6 and exons 16–17 as kl-granules were observed in both *maca*^*null/+*^ and *maca*^*null/null*^ (S14 Fig). Again, because of the limited quantitativeness of the FISH assay, we did not reliably detect *kl-5* transcript level reduction in *maca*^*null/null*^ spermatocytes.

### Maca is enriched at Y-loop A/C in spermatocyte nucleus

**Fig 12:**
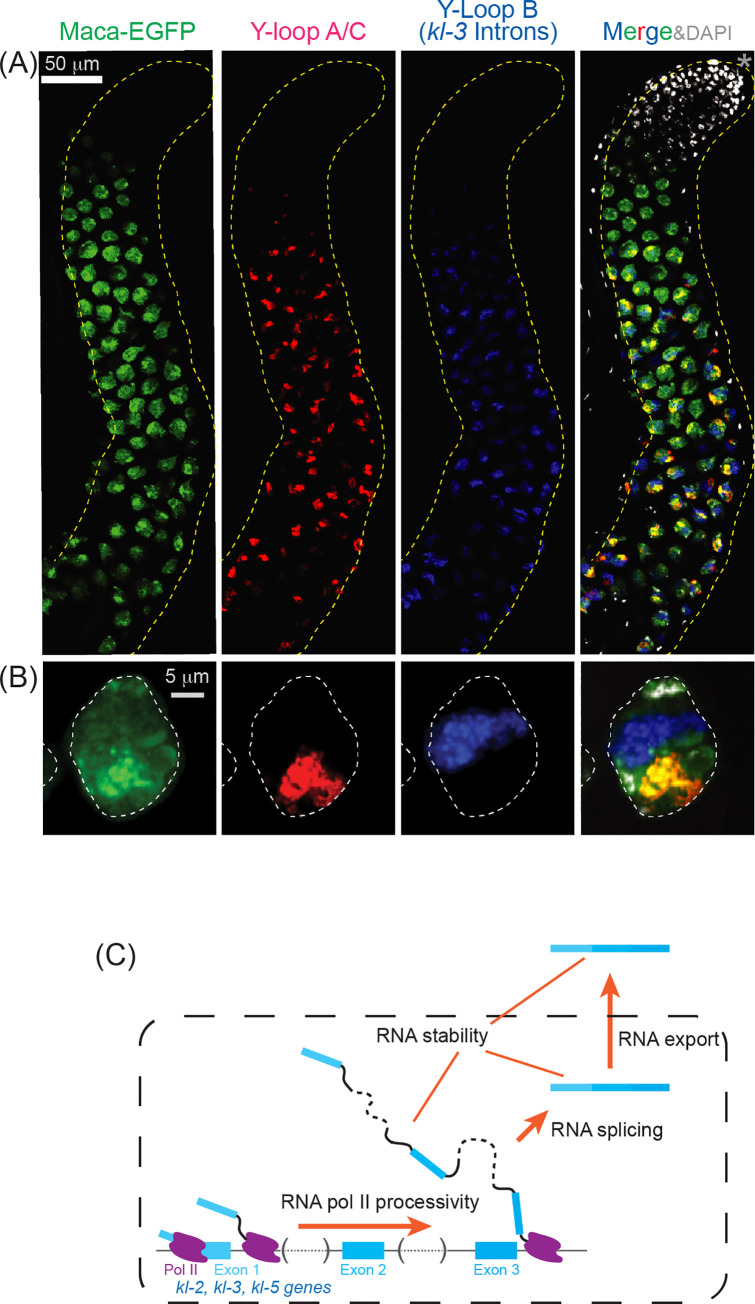
Maca is enriched at Y-loop A/C in the spermatocyte nucleus. (A, B) RNA FISH to visualize Y-loops in *maca-EGFP* testis. Maca-EGFP (green), Y-loop A/C (Cy3-(AAGAC)_6_, red), Y-loop B (*kl-3* introns, Cy5-(AATAT)_6_, blue), and DAPI (white). (A) Apical regions of testes including spermatocyte growth region are shown. The apical tip of testis is marked by *. Scale bar is 50 μm. (B) Single spermatocyte nuclei (white dashed line) at late stage. Scale bar is 5 μm. (C) Model for Maca functions on *kl-2*, *kl-3*, and *kl-5* transcripts.

Considering the effects of *maca* mutation on the *kl-2*, *kl-3*, and *kl-5* transcript levels and splicing, we wondered if Maca colocalizes with *kl-2*, *kl-3*, and/or *kl-5* transcripts in the spermatocyte nucleus. To test this, we performed RNA FISH using the *maca-EGFP* transgenic fly testes in the otherwise wild-type background probing different regions within the *kl-2*, *kl-3*, and *kl-5* transcripts including the Y-loops (Figs 12A, 12B, S15A, S15B and S15E–S15L). Interestingly, while Maca-EGFP resides in the entire region within the spermatocyte nucleus, it was enriched at Y-loop A/C (Fig 12A and 12B). When the dyes on the probes for Y-loop A/C and Y-loop B (*kl-3* introns) were swapped, Maca-EGFP still showed an enriched localization at Y-loop A/C (S15A and S15B Fig). Maca-EGFP and Y-loop A/C exhibited similar spatiotemporal expression patterns during spermatocyte growth; they started to be expressed in early spermatocytes, exhibited colocalization during the spermatocyte growth phase, and became undetectable prior to the first meiotic metaphase (Fig 12A and 12B). The localization of Y-loop A/C in the spermatocyte nucleus was distinct from those of Y-loop B and the *kl-2* and *kl-3* transcripts in the control testes (Figs 12A, 12B and S15A–S15D). The distinct localizations of Y-loop A/C and Y-loop B were observed in *maca*^*null/null*^ as well (S16A–S16D Fig), revealing that *maca* is not essential for their distinct localizations. *ks-1* mRNA resides at the cytoplasmic granule that is different from the kl-granules, where *kl-5* mRNA resides, in the late-stage spermatocytes in the control testes (S16E Fig). The distinct *ks-1* mRNA cytoplasmic granule was observed in *maca*^*null/null*^ as well (S16F Fig), revealing that *maca* is not essential for the distinct cytoplasmic localization of *ks-1* mRNA.

## Discussion

In this study, we identified a novel RNA-binding protein Maca in *Drosophila*. We showed that Maca is specifically expressed in spermatocytes in testes, resides in the nucleus enriched at Y-loop A/C, and is required for a properly orchestrated organization of late-stage individualization complexes, mature motile sperm, and male fertility. The *maca* mutant phenotypes were reminiscent of those of male fertility factor genes *kl-2*, *kl-3*, and *kl-5* (*kl* genes) which have gigantic introns. Unbiased genome-wide proteome and transcriptome analyses revealed that *maca* is required for proper expression of the mRNAs and proteins of *kl* genes. *kl-2* was particularly strongly downregulated in *maca* mutant testes at both mRNA and protein levels. RT-qPCR confirmed the severe *kl-2* mRNA level reduction and also showed a reduction of *kl-2* pre-mRNA. *maca* was also required for proper splicing of the *kl-3* transcript.

The *kl* genes span megabases with gigantic introns containing satellite DNAs. Their transcription gradually progresses in spermatocytes as the cells grow for ~90 hours. The extended time period required for the complete transcription of the *kl-3* and *kl-5* genes due to the presence of the gigantic introns was proposed to function as a ‘developmental timer’ for the spermatocyte growth [40]. In this study, we also showed that the complete transcription of the *kl-2* gene also takes an extended time during the spermatocyte growth. Thus, the *kl-2* transcription may function as a developmental timer as well where the presence of the gigantic introns is important.

Considering that Maca is a nuclear RNA-binding protein, it likely regulates nuclear RNA processes rather than cytoplasmic ones such as translation. Poly-A+ RNA-seq, RT-qPCR, and RNA FISH together showed that in *maca*^*null/null*^ testes, (1) spliced *kl-2* transcript level was severely reduced, more severely than unspliced *kl-2* transcripts, (2) the degree of reduction of unspliced *kl-2* transcripts gradually increased from the 5′ end region to the 3′ end region of the *kl-2* pre-mRNA, (3) spliced *kl-3* and *kl-5* transcript levels were reduced, (4) *kl-3* splicing was dysregulated with exon 13 skipped, and (5) cytoplasmic mRNA granules of *kl-2*, *kl-3*, and *kl-5* were absent in late-stage spermatocytes. Based on these results, we propose models for Maca functions below (Fig 12C).

Complete transcription of extremely long genes like the *kl* genes requires a high processivity of RNA polymerase II (pol II). High processivity of pol II is more important for the *kl* genes since they contain highly repetitive sequences such as satellite DNAs. Repetitive sequences can form high-order structures in DNA, RNA, and DNA-RNA hybrids, which may inhibit transcription elongation of pol II. In fact, repeat expansion (as in trinucleotide expansion diseases) and tandem arrays slow an elongating pol II and cause premature dissociation [43–46]. We propose that Maca helps high processive transcription of the *kl* genes, allowing pol II to transcribe completely through their extremely long, repetitive DNA sequences (Fig 12C).

It must be also challenging to properly splice extremely long transcripts like the *kl* transcripts, where exons are separated by megabase-sized introns and a 5′ splice site and a 3′ splice site that are extremely distant in a primary sequence need to be brought together. Furthermore, high-order structures in RNA caused by repetitive sequences are thought to pose a big challenge in splicing. Thus, we also propose that Maca plays important roles in the splicing of the *kl* pre-mRNAs (Fig 12C). The observed dysregulation of *kl-3* splicing (exon 13 skipping), in *maca* mutant spermatocytes supports this model. Kl-3 gene has the gigantic intron 13 (between exon 13 and exon 14) while its intron 12 is only 274 nt long. Properly splicing out the gigantic intron 13 and joining the exons 13 and 14 requires Maca functions.

The extremely long *kl* transcripts are thought to be more easily degraded since they have more access points for degradation enzymes/chemicals than normal length transcripts. We propose that Maca may protect the *kl* transcripts from degradation (Fig 12C). Finally, Maca might be required for the export of *kl* mRNAs from the nucleus to the cytoplasm, which can also explain the observed reduction of *kl* mRNAs (in the cytoplasm). These proposed molecular functions of Maca are not mutually exclusive. Maca likely plays roles in multiple of these functions.

We found that Maca is enriched at Y-loop A/C in the spermatocyte nucleus. Considering that *kl-5* mRNA levels were reduced in the *maca* mutant testes, this colocalization may suggest that Maca directly binds the *kl-5* transcripts and thereby promote their transcription and/or processing or prevent their premature degradation. We note that while Maca-EGFP signal is stronger at Y-loop A/C, it was detectable in the entire nucleus (Figs 12A, 12B and S15). Thus, Maca may directly bind the *kl-2* and *kl-3* transcripts and promote their transcription and/or processing or prevent their premature degradation as well, which is supported by our results that the *kl-2* and *kl-3* mRNA levels were reduced and the *kl-3* splicing was dysregulated in the *maca* mutant testes. It will be important to determine direct RNA-binding targets of Maca in future studies.

Some other RNA-binding proteins are enriched at the Y-loops in the spermatocyte nucleus and are required for male fertility. Blanks [40], Hephaestus (Heph)[40], Boule [47], Hrb98DE [48], Pasilla [48,49], and Rb97D [50] are among them. Only two of them, Blanks and Heph, seem to play roles in Y-loop gene expression in spermatocytes [40]. Blanks colocalizes with Y-loop B (*kl-3* introns) and is required for *kl-3* mRNA expression, proper individualization complex organization in late-stage spermatocytes, and mature motile sperm formation [40,51]. In our previous study, we performed poly-A+ RNA-seq of *blanks* mutant testes, which revealed downregulation of *kl-3* mRNA [51]. In our *blanks* mutant poly-A+ RNA-seq results, we did not observe splicing dysregulation of *kl-3* or any other fertility factor gene transcripts, unlike in *maca* mutant. Since both *kl-3* nuclear transcript levels and cytoplasmic mature *kl-3* mRNA levels in spermatocytes were reduced in *blanks* mutant spermatocytes judged by RNA FISH, it was proposed that Blanks may increase the pol II processivity by binding to *kl-3* nascent transcripts in the nucleus [40]. Heph colocalizes with Y-loop A/C and is required for *kl-3*, *kl-5*, and *ks-1* (*ory*) mRNA expression, proper individualization complex organization in late-stage spermatocytes, and mature motile sperm formation. Since cytoplasmic mature *kl-5* mRNA granules were diminished without a large decrease in the nuclear *kl-5* transcript levels in *heph* mutant spermatocytes, it was proposed that Heph may play crucial roles in *kl-5* transcript processing such as splicing and/or preventing premature degradation rather than in the transcription step. The protein and mRNA abundance of Blanks and Heph were not significantly changed in the *maca*^*null/null*^ testes compared with the controls in our mass spec and poly-A+ RNA-seq data, excluding the possibility that the *maca*^*null/null*^ phenotypes are mediated through changes in Blanks and Heph. Thus, multiple RNA-binding proteins seem to regulate and be required for the successful expression of the gigantic male fertility factor genes. As far as we know, Maca is the first protein that is demonstrated to be required for proper splicing of a male fertility factor gene and for the *kl-2* transcript abundance.

Human Dystrophin gene is a causative gene for Duchenne Muscular Dystrophy and spans megabases with gigantic introns rich in repetitive DNA as in the *Drosophila kl* genes. Exon-skipping induced by antisense oligonucleotides to skip the exons containing premature stop codon mutations or translation reading-frame shift mutations is used as a therapeutic approach for Duchenne Muscular Dystrophy[52]. Our work showing that Maca is crucial for proper splicing of the *kl* gene (without Maca exon-skipping occurs) gives us useful insight into the splicing regulation of genes containing gigantic introns.

BLAST Homology search against *Drosophila* proteins using the Maca amino acid sequence identified Sex lethal (Sxl) as the top hit. Like Maca, Sxl has two RRMs, and Maca and Sxl share 41% (71/175) identify and 61% (106/175) similarity in their amino acid sequences of the regions containing the two RRMs. Sxl regulates alternative splicing of specific mRNAs including *sxl* itself, *tra*, and *msl-2*, and is a crucial regulator for sex determination [53]. We showed that Maca is dispensable for proper alternative splicing of these three mRNAs (S12 Fig). Sxl and Maca might have been produced by duplication of an ancestral gene and have evolved to have their own specific functions in mRNA regulation. BLAST Homology search against human proteins using Maca identified ELAV-like protein 4 (ELAV4, Hu antigen D) as the top hit. ELAV4 has three RRMs, and Maca and ELAV4 share 37% (79/211) identity and 55% (118/211) similarity in the regions containing the first two RRMs of ELAV4. ELAV4 is neuron-specific and ELAV4-binding stabilizes bound mRNAs [54]. Evolutional and functional links between these two proteins are unknown.

In summary, by identifying and characterizing the novel RNA-binding protein Maca, which colocalizes with the Y-loop A/C and is required for successful expression of Y-chromosome male fertility factor genes and spermatogenesis, our studies provide insights into the molecular mechanism in the spatiotemporally highly regulated gene expression program during spermatogenesis.

## Materials and methods

### Fly strains

We generated the *maca*^*null*^ and *maca*^*R1*^ strains by introducing deletions within the *maca* coding region using genome editing with a CRISPR/Cas9 system as we previously reported [36,55–57]. The transgenic *maca-EGFP* fly strain was produced as we previously reported [58]. Genomic fragment containing the *maca* cDNA flanked by ~2.5 kbp upstream and ~0.9 kbp downstream genomic sequences was cloned. EGFP gene was fused to the C-terminus of the *maca* coding sequence in-flame within the genomic fragment. This fragment was then inserted in a pattB plasmid vector [59,60]. The resulting P(change before submission)*maca-EGFP*) was integrated site-specifically into the 51C1 landing site within fly genome using the BDSC:24482 strain and the PhiC31 system [59,60]. The mini-white gene (*w*^*+mC*^) was removed by using the Cre-Lox system.

*kl-3-3xFLAG* fly strain [40] was a kind gift from Dr. Yukiko Yamashita. Df(3R)Exel6174 (BDSC: 7653), *dj-GFP* (BDSC: 5417), *bam-Gal4-VP16* (BDSC: 80579), *UAS-kl-3*^*TRiP*.*HMC03546*^ (BDSC: 53317), *UAS-kl-5*^*TRiP*.*HMC03747*^ (BDSC: 55609), and *UAS-white*^*TRiP*.*HMS00004*^ (BDSC: 33613) were obtained from the Bloomington Stock Center (BDSC). *UAS-kl-2*^*GD8807*^ (VDRC: v19181) and *UAS-white*^*GD14981*^ (VDRC: v30033) were obtained from the Vienna Drosophila Resource Center.

### Fertility assay

For the male fertility assay, one test male was mated with five virgin wild-type (OregonR) females in a vial [56,61]. After 3 days, the five OregonR females were transferred to a new vial (vial 1). Every two days, the five OregonR females were transferred to a new vial until a total of 4 vials were obtained. The five OregonR females were removed from the last vial (vial 4) after two days. The total number of progenies in these four vials was counted. At least five individual males were tested per genotype. For the female fertility assay, five test virgin females were mated with three wild-type (OregonR) males in a cage with a 6-cm grape juice agar plate with wet yeast paste (Genesee) [56,61,62]. The grape juice agar plate was exchanged with a fresh one every day. The number of eggs laid on the third grape juice agar plate (set up on Day 3 and recovered on Day 4) was counted. Then, this grape juice agar plate was kept for one more day at 25C and the number of hatched eggs was counted. At least three individual cages were tested per genotype.

### Maca antibodies

We expressed a recombinant full-length Maca protein as an N-terminally 6xHis-MBP-fused form using a modified pET vector in *E*. *coli* [63] and 6xHis-MBP-HRV3Csite-Maca was purified using Ni-sepharose (GE Healthcare) as we previously performed for other proteins [56,64]. Then the 6xHis-MBP tag was cleaved off with HRV3C protease and Maca protein was further purified using HiTrap SP (GE Healthcare). Using the purified Maca protein as an antigen, we produced poly-clonal anti-Maca sera in rabbits (Pocono Rabbit Farm & Laboratory, Inc.). The rabbit poly-clonal anti-Maca antibodies in the sera were affinity purified using the purified His-MBP-HRV3Csite-Maca protein described above and Affigel-10 (Bio-rad) as instructed in the product manual.

### Mass-spec

Testes from 2–3 day old flies were hand-dissected in 1X PBS, flash-frozen in liquid nitrogen, and stored in -80°C until use. The frozen testes were placed in RIPA buffer (50 mM Tris-HCl [pH 7.4], 150 mM NaCl, 1% [v/v] IGEPAL CA-630, 0.1% [w/v] sodium dodecyl sulfate (SDS), 0.5% [w/v] sodium deoxycholate, 1 mM ethylenediaminetetraacetic acid (EDTA), and 0.5 mM phenylmethylsulfonyl fluoride (PMSF)) on ice, homogenized using a pestle, and kept on ice for 30 min. The homogenates were clarified by centrifugation at 21000g at 4°C for 10 min. The protein concentration was determined using the BCA protein assay kit (Pierce) and the homogenates were diluted with RIPA buffer to final 0.5 mg/mL protein concentration. The protein homogenate quality was examined using SDS-PAGE followed by Coomassie staining. The homogenates were aliquoted into 100 uL per tube, flash-frozen in liquid nitrogen, and stored in -80°C until use.

Proteins in the homogenates were reduced with 2.5 μL of 15 mg/mL Dithiothreitol (DTT) in 100 mM triethyl ammonium bicarbonate (TEAB) for 1 hr at 57°C with mixing. After the pH was adjusted to 8.0 with 500 mM TEAB, proteins were alkylated using 3 μL of 36 mg/mL Iodoacetomide for 10–15 min at room temperature in the dark. Proteins were precipitated by adding 8 x volume of TCA/acetone on ice and incubating at -20°C overnight. Protein pellets were washed once with iced acetone, dried, re-constituted in 95 μL 100 mM TEAB in 5% acetonitrile, sonicated for 20 min, and proteolyzed with 3 μg Trypsin (Pierce) at 37°C overnight. Peptides in each sample (50 μg) were labeled with Isobaric mass tags TMT16pro reagents (Thermo Fisher-Pierce, LOT # UH290430) by adding a unique TMT 16 plex pro reagent in 20 μL acetonitrile to each sample. The TMT labeled peptide from all samples were combined into total 800 μg combined TMT-labeled peptides, aliquoted into 3 aliquots (266 μg each), and dried.

Fractionation by basic reverse phase (bRP) chromatography: 1 aliquot 266 μg of combined TMT labeled dry peptides was re-constituted in 50 μl 50 mM TEAB buffer, cleaned on 500 μL Pierce Detergent Removal Spin Columns (Pierce) and eluted in 50 μL TEAB Buffer. 1950 μL 10 mM TEAB in water were added to the eluted peptides. Peptides were fractionated by basic reverse phase (bRP) chromatography using 85 min gradient from 100% solvent A (10mM TEAB in water) to 100% solvent B (10 mM TEAB in 90% acetonitrile) at a 250 μl/min flow rate on a XBridge C18 Column, 5 μm, 2.1 x 100 mm column (Waters) with a XBridge C18 Guard Column, 5 μm, 2.1 x 10 mm (Waters), using an Agilent HPLC system 1200 series binary capillary pumps. Collected 84 fractions were re-combined into 24 fractions for LC-MS/MS analysis.

LC-MS/MS analysis: Peptides in 24 fractions with calculated average amount per fraction 11 μg were re-constituted in 125 μL 2% acetonitrile/0.1% formic acid and 6 μL of calculated average amount / fraction 533 ng was analyzed by nano-LC-MS/MS. Peptides were analyzed on a nano-LC-Orbitrap-Lumos-Fusion-ETD (Thermo Fisher Scientific) in FTFT, interfaced with an EasyLC1200 series using reverse-phase chromatography (2%–90% acetonitrile/0.1% formic acid gradient over 100 min at 300 nl/min) on a 75 μm x 150 mm ProntoSIL-120-5-C18 H column 3 μm, 120 Å (BISCHOFF). Eluted peptiMSs were sprayed into an Orbitrap-Lumos-Fusion mass spectrometer through a 1 μm emitter tip (New Objective) at 2.4 kV. Survey scans (Full ms) were acquired on an Orbi-trap within 375–1600 Da m/z using a Data dependent Top 15 method with dynamic exclusion of 15 s. Precursor ions were individually isolated with 0.7 Da, fragmented (MS/MS) using an HCD activation collision energy 38. Precursor and fragment ions were analyzed at a resolution of 120,000 and 60,000, respectively, and the client setting were used for the other parameters in TMT experiment

MSMS data analysis: Tandem MS2 spectra (signal/noise >2) were processed by Proteome Discoverer (v2.4 ThermoFisher Scientific) using Files RC option (recalibration with appropriate database). MS/MS spectra were searched with Mascot v.2.6.2 (Matrix Science, London, UK) against RefSeq2017_83_Drosophila Melanogaster database, a small database containing enzymes, BSA. And a database containing kl3 proteins with deletions. Trypsin as an enzyme, missed cleavage 2, precursor mass tolerance 5ppm, fragment mass tolerance 0.01Da; Carbamidomethyl on C, TMT 16pro on N-terminus and on K as fixed, Oxidation on M, Deamidation on NQ as variable modifications. Peptide identifications from the Mascot searches were processed within the Proteome Discoverer2.4 and Percolator to identify peptides with a confidence threshold of 1% False Discovery Rate, based on an auto-concatenated decoy database search, and calculate the protein and peptide ratios. Only Peptide Rank 1 were considered. Peptides used for quantifications: unique; Only non-modified peptides were used for normalization. All peptides used for protein roll-up. Precursor sn>1.5, Reporter SN >3, Isolation Interference <30.

Database search against RefSeq2017_83_Drosophila Melanogaster identified 6559 proteins at high, medium, and low confidence with at least 1 peptide identified at 1% FDR, Rank1 (Percolator FDR).

### Western blot

Lysates of hand-dissected testes and tissues were prepared by homogenizing in RIPA buffer (50 mM Tris-HCl [pH 7.4], 150 mM NaCl, 1% [v/v] IGEPAL CA-630, 0.1% [w/v] sodium dodecyl sulfate (SDS), 0.5% [w/v] sodium deoxycholate, 1 mM ethylenediaminetetraacetic acid (EDTA), 5 mM dithiothreitol, and 0.5 mM phenylmethylsulfonyl fluoride (PMSF)) [56,57,65,66]. The homogenates were clarified by centrifugation at 21000g at 4°C for 10 min, and the protein concentration was determined using the BCA protein assay kit (Pierce) as needed. Fifteen μg of total protein was loaded per lane for Western blot. The sources and dilutions of the primary antibodies were as below. Rabbit anti-Maca (1/10000, generated in this study), rabbit anti-Tubulin (1/1000, Sigma, T3526), mouse anti-alpha-Tubulin (1/1500, Sigma, T9026), mouse anti-FLAG (1/1000, Sigma, F1804). IRDye 800CW goat anti-mouse IgG, IRDye 800CW goat anti-rabbit IgG, IRDye 680RD goat anti-mouse, and IgG IRDye 680RD goat anti-rabbit were used as secondary antibodies. The membranes were scanned on an Odyssey imaging system (Licor).

### RT-qPCR and RT-PCR

RNA from testes and whole adult flies was prepared using miRVana (Thermo Fisher Scientific) and TRIzol (Thermo Fisher Scientific), respectively. RNAs were treated with Turbo DNase (Thermo Fisher Scientific) and were reverse-transcribed into cDNA using a random hexamer primer and AMV Reverse Transcriptase (NEB). qPCR was performed using SsoAdvanced Universal SYBR Green Supermix on CFX96 (Biorad). PCR was performed using GoTaq Green Master Mix (Promega) or Phusion DNA polymerase (Thermo Fisher Scientific). The primers used are listed in S1 Table.

### Immunostaining

Testes hand-dissected from 2–5 day old flies were placed in a drop of ~5 ul of PBS (137 mM NaCl, 2.7 mM KCl, 1.5 mM KH_2_PO_4_, 8.1 mM Na_2_HPO_4_, pH 7.4) on a square glass cover slip. A glass microscope slide was placed gently placed over the cover slip to squash the testes. Slides containing squashed testes were snap-frozen in liquid nitrogen and then immediately transferred to a prechilled slide rack filled with ice-cold 95% ethanol (spectrophotometric grade, methanol-free) and incubated at -20°C for 10 min. Slides were transferred to a slide rack filled with PBS containing 0.1% Triton X-100 (PBS-T) and 4% formaldehyde (Methanol-free, Pierce) and incubated at room temperature for 7 min. Slides were washed in a slide rack filled with PBS. Slides were transferred to PBS-T and incubated for 30 min at room temperature to permeabilize cell membranes. Slides were washed in PBS for 5 min at room temperature. Positions of squashed testes on slides were marked with a hydrophobic barrier pen. After 30–40 ul of primary antibody diluted in PBS-T (rat anti-Vasa (DSHB, AB_760351, 1/100; mouse anti-Hts (1B1) (DSHB, AB_528070, 1/100)) was applied to testes, the slides were incubated in a moist, dark chamber for 2 hr at room temperature. Slides were washed with PBS for 5 min at room temperature three times. After 30–40 ul of fluorophore-conjugated secondary antibody diluted in PBS (Alexa Fluor 594 Donkey anti-rat Igg (ThermoFisher, A21209), 1/100; Alexa Fluor 488 Donkey anti-mouse Igg (ThermoFisher, A21202), 1/100) was applied to testes, the slides were incubated in a moist, dark chamber for 2 hr at room temperature. After the slides were washed with PBS for 5 min at room temperature twice, 30–40 ul of VECTASHIELD with DAPI (Vector Labs) was applied to testes. Testes Images were collected with the Zeiss LSM700 confocal microscope at the Johns Hopkins University School of Medicine Microscope Facility.

### RNA fluorescent in situ hybridization

Testes from 2–5 day old flies were hand-dissected in PBS and fixed in 4% formaldehyde (Methanol-free, Pierce) in PBS for 30 minutes at room temperature. Testes were washed briefly in PBS and permeabilized in 1 mL 70% ethanol overnight on a rocker in a 4°C cold room. After rinsed with 300 ul wash buffer (2X saline-sodium citrate (SSC. 300 mM NaCl, 30 mM trisodium citrate, pH 7.0), 10% formamide), testes were hybridized with 100 uL of 50 nM (for satellite repeat. Integrated DNA Technologies) or 100 nM (for all the others. Biosearch Technologies, Inc) probes in hybridization buffer (wash buffer, 10% dextran sulfate (Sigma, D8906), 1 mg/mL *E*. *coli* tRNA (Sigma, R8759), 2 mM Vanadyl Ribonucleoside complex (NEB, S1402), 0.5% BSA (Ambion, AM2618)) overnight at 37°C. After washed with 200 ul wash buffer three times for 20 minutes each at 37°C, testes were mounted in 30–40 ul of VECTASHIELD with DAPI (Vector Labs). During these procedures, sample tubes and slides containing testes with FISH probes were covered with aluminium foil to minimize photo-bleaching. Testes Images were collected with the Zeiss LSM700 confocal microscope at the Johns Hopkins University School of Medicine Microscope Facility. The FISH probes used are listed in S2 Table.

### S2 cell imaging

N-terminally EGFP-fused Maca and mCherry coding sequences were inserted in pAc5.1B/V5-HisB plasmid. S2 cells were co-transfected with these two plasmids using Effectene transfection reagent kit (Qiagen, 301425). Three days after transfection, cells were transferred on a glass slide in a 6 well plate and were grown overnight. After the cell medium was removed, cells were fixed with 1 ml 4% formaldehyde in PBS at room temperature for 30 min. After washed with 1 ml PBS, cells were mounted on glass slides with 40 ul of VECTASHIELD with DAPI (Vector Labs). Images were acquired using the Zeiss LSM700 confocal microscope at the Johns Hopkins University School of Medicine Microscope Facility.

### Small RNA and mRNA sequencing

Poly-A+ mRNA purification was performed as previously reported[67]. small RNA libraries and poly-A+ mRNA libraries were prepared, sequenced on Hiseq2500 (Illumina), and were analyzed, as previously reported [68,69].

## Supporting information

S1 Fig *maca* mutant flies are male-sterile and female-fertile. (A) Male fertility assay. The numbers of the progeny flies obtained from the crosses between test males and *OregonR* wild-type virgin females are shown. Mean +/- SD (n = 5 biological replicates). P-value <0.05 (Student’s t-test) are indicated by *. Trans-heterozygous mutant flies *maca*^*null/Df*^ and *maca*^*R1/Df*^ have the *maca*^*null*^ or *maca*^*R1*^ allele and the *Df(3R)Exel6174* chromosomal deficiency allele uncovering the *maca* gene. (B, C) Female fertility assay. (B) Numbers of eggs laid by test virgin females crossed with *OregonR* wild-type males and (C) hatching rates from the eggs. Mean +/- SD (n = 3 biological replicates). P-value <0.05 (Student’s t-test) are indicated by *.
(TIF)

S2 Fig *maca* mutant flies lack mature motile sperm. Confocal imaging of whole testes and seminal vesicles from indicated genotypes in the *dj-GFP* background. DJ-GFP (green), DAPI (blue). DJ-GFP is expressed in sperm. The apical tip of testis is marked by *. Seminal vesicles are indicated by white dashed line. Scale bars are 100 μm.
(TIF)

S3 Fig Maca resides in the nucleus in S2 cells. Confocal imaging of S2 cells transiently expressing EGFP-Maca. mCherry signal marks the cytoplasm while DAPI signal marks the nucleus. EGFP-Maca resides in the nucleus. Scale bar is 5 μm.
(TIF)

S4 Fig Poly-A+ RNA-seq and mass-spec to compare mRNA and protein abundance between *maca*^*null/null*^ and *maca*^*null/+*^. (A) Volcano plot showing log_2_(fold-change) and -log10(adjusted P-value) of protein abundance in *maca*^*null/null*^ testes compared with *maca*^*null/+*^ testes determined by mass-spec. Light blue: Proteins with adjusted P-value (= FDR) <0.05 and fold-change < 1 in *maca*^*null/null*^ testes compared with *maca*^*null/+*^. Dark blue: Proteins with adjusted P-value <0.05 and fold-change > 1 in *maca*^*null/null*^ testes compared with *maca*^*+/+*^, *maca*^*null/+*^, and *maca*^*null/null*^ with *maca-EGFP* rescue transgene, consistently. Full list shown in Fig 3. Pink: Proteins with adjusted P-value <0.05 and fold-change > 1 in *maca*^*null/null*^ testes compared with *maca*^*null/+*^. Red: Proteins with adjusted P-value <0.05 and fold-change > 1 in *maca*^*null/null*^ testes compared with *maca*^*+/+*^, *maca*^*null/+*^, and *maca*^*null/null*^ with *maca-EGFP* rescue transgene, consistently. Full list shown in Fig 3. Gray: Proteins with adjusted P-value > = 0.05 and fold-change < 1 in *maca*^*null/null*^ testes compared with *maca*^*null/+*^. (B) Volcano plot showing log_2_(fold-change) and -log10(adjusted P-value) of each mRNA in *maca*^*null/null*^ testes compared with *maca*^*null/+*^ testes determined by poly-A+ RNA-seq. Light blue: Proteins with adjusted P-value (= FDR) <0.01 and fold-change < 1 in *maca*^*null/null*^ testes compared with *maca*^*null/+*^. Dark blue: Proteins with adjusted P-value <0.01 and fold-change > 1 in *maca*^*null/null*^ testes compared with *maca*^*+/+*^, *maca*^*null/+*^, *maca*^*null/+*^ with *maca-EGFP* rescue transgene, and *maca*^*null/null*^ with *maca-EGFP* rescue transgene, consistently. Full list shown in Fig 4B. Pink: Proteins with adjusted P-value <0.01 and fold-change > 1 in *maca*^*null/null*^ testes compared with *maca*^*null/+*^. Red: Proteins with adjusted P-value <0.01 and fold-change > 1 in *maca*^*null/null*^ testes compared with *maca*^*+/+*^, *maca*^*null/+*^, *maca*^*null/+*^ with *maca-EGFP* rescue transgene, and *maca*^*null/null*^ with *maca-EGFP* rescue transgene, consistently. Full list shown in Fig 4A. Gray: Proteins with adjusted P-value > = 0.01 and fold-change < 1 in *maca*^*null/null*^ testes compared with *maca*^*null/+*^. *kl-2*, *kl-3*, *kl-5*, *JYalpha*, *CG5399*, *and CG31213*, *which* exhibit significant dysregulation in both protein and mRNA levels in *maca*^*null/null*^ testes compared with all the other genotypes consistently, are labeled.
(TIF)

S5 Fig Testis small RNA-seq showed no major changes in *maca*^*null/null*^. Scattered plots of normalized number of reads (reads per million non-rRNA-mapping genome-mapping reads) of (A) miRNAs and endo-siRNA (esi-1.1, esi-1.2, and esi-2.1) and (B) sense and antisense piRNAs in testis small RNA-seq. Each dot represents a unique miRNA, endo-siRNA, or piRNA cluster (transposon-sense or transposon-antisense mapping). Mean +/- SD (n = 3 biological replicates).
(TIF)

S6 Fig Poly-A+ RNA-seq read mapping counts for *kl-2*. Poly-A+ RNA-seq normalized read counts (counts per million mapped reads, CPM) in the *kl-2* gene region. Gene structures are shown with exons (cyan), introns (black line), intronic satellite DNA repeats (dashed line in parentheses). Regions targeted by RNA FISH probes used in Figs 8, S10 and S15 (green and red).
(TIF)

S7 Fig Poly-A+ RNA-seq read mapping counts for *kl-3*. Poly-A+ RNA-seq normalized read counts (counts per million mapped reads, CPM) in the *kl-3* gene region. Splicing ratios among (1) exon 12—exon 13, (2) exon 13—exon 14, and (3) exon 12—exon 14 (= exon 13-skipped) in determined by LeafCutter are shown. Gene structures are shown with exons (cyan), introns (black line), intronic satellite DNA repeats (dashed line in parentheses). Regions targeted by RNA FISH probes used in Figs 10, S9, S13 and S15 (blue, green, and red).
(TIF)

S8 Fig Poly-A+ RNA-seq read mapping counts for *kl-5*. Poly-A+ RNA-seq normalized read counts (counts per million mapped reads, CPM) in the *kl-5* gene region. Gene structures are shown with exons (cyan), introns (black line), intronic satellite DNA repeats (dashed line in parentheses). Regions targeted by RNA FISH probes used in S9, S14, S15 and S16 Figs (blue, green, and red).
(TIF)

S9 Fig No major reduction of *ks-1* (*ory*) protein and mRNA levels in *maca*^*null/null*^ testes. (A) Relative abundance of Ks-1 (ORY) protein in testes determined by mass spec with TMT labeling. Mean +/- SD (n = 4 biological replicates). Adjusted P-values <0.05 are indicated by *. (B) Normalized counts (reflecting relative abundance) of *ks-1* (*ory*) mRNA determined by poly-A+ RNA-seq. Mean +/- SD (n = 3 biological replicates). Adjusted P-values <0.01 are indicated by *. (C) poly-A+ RNA-seq normalized read counts (counts per million mapped reads, CPM) in the *ks-1* (*ory*) gene region. Gene structures are shown with exons (cyan), introns (black line), intronic satellite DNA repeats (dashed line in parentheses).
(TIF)

S10 Fig *kl-2*, *kl-3*, or *kl-5* RNAi knockdown depletes their cytoplasmic mRNAs in late-stage spermatocytes. RNA FISH to visualize (A) *kl-2*, (B) *kl-3*, and (C) *kl-5* transcripts in late-stage spermatocytes. Single spermatocyte nuclei (white dashed line) at stage 4 of their transcript expression. Scale bars are 5 μm. Cytoplasmic mRNA granules (kl-granules) are indicated by cyan arrows. (A) *kl-2* introns 1–2 (green), exon 8 (red), and DAPI (white). (B) *kl-3* exon 1 (blue), introns (Y-loop B, Alexa488-(AATAT)_6_, green), exon 14 (red), and DAPI (white). (C) *kl-5* exons 1–6 (blue), introns (Y-loop A/C, Alexa488-(AAGAC)_6_, green), exons 16–17 (red), and DAPI (white).
(TIF)

S11 Fig *kl-2*, *kl-3*, or *kl-5* RNAi knockdown causes loss of mature motile sperm and disorganized late-stage individualization complexes. (A) Confocal images of dissected seminal vesicles stained with DAPI. Motile sperm is absent in *RNAi*^*kl-2*^, *RNAi*^*kl-3*^, and *RNAi*^*kl-5*^. Scale bars are 50 μm. (B) Confocal images of the early-stage individualization complexes. Phalloidin (Actin, green), DAPI (blue). Scale bars are 10 μm. (C) Confocal images of the late-stage individualization complexes. Phalloidin (Actin, green). Scale bars are 10 μm.
(TIF)

S12 Fig RT-PCR assay for *kl-3* exon 13 skipping and *sxl*, *tra*, and *msl2* alternative splicing. Agarose gel electrophoresis of RT-PCR products to test (A) *kl-3* mRNA exon 13 skipping and alternative splicing of (B) *sxl*, (C) *tra*, and (D) *msl2* mRNAs. Whole fly total RNAs were used for RT-PCR.
(TIF)

S13 Fig RNA FISH for *kl-3* transcripts in testes. RNA FISH to visualize *kl-3* transcripts expression in (A, B) *maca*^*null/+*^ and (C, D) *maca*^*null/null*^ testes. Exon 1 (blue), Y-loop B (*kl-3* introns, Alexa488-(AATAT)_6_, green), exon 14 (red), and DAPI (white). (A, C) Apical regions of testes including the spermatocyte growth region are shown. The apical tip of testis is marked by *. Scale bars are 50 μm. (B, D) Single spermatocyte nuclei (white dashed line) at each stage of *kl-3* expression. Cytoplasmic mRNA granules are indicated by cyan arrows. Scale bars are 5 μm.
(TIF)

S14 Fig RNA FISH for *kl-5* transcripts in testes. RNA FISH to visualize *kl-5* transcripts expression in (A, B) *maca*^*null/+*^ and (C, D) *maca*^*null/null*^ testes. Exons 1–6 (blue), Y-loop A/C (*kl-5* introns, Alexa488-(AAGAC)_6_, green), exons 16–17 (red), and DAPI (white). (A, C) Apical regions of testes including the spermatocyte growth region are shown. The apical tip of testis is marked by *. Scale bars are 50 μm. (B, D) Single spermatocyte nuclei (white dashed line) at each stage of *kl-5* expression. Cytoplasmic mRNA granules are indicated by cyan arrows. Scale bars are 5 μm.
(TIF)

S15 Fig Maca is enriched at Y-loop A/C in the spermatocyte nucleus. (A, B) RNA FISH to visualize Y-loops in *maca-EGFP* testis. Maca-EGFP (green), Y-loop A/C (Cy5-(AAGAC)_6_, blue), Y-loop B (*kl-3* introns, Cy3-AATAT)_6_, red), and DAPI (white). (A) Apical regions of testes including spermatocyte growth region are shown. The apical tip of testis is marked by *. Scale bar is 20 μm. (B) Single spermatocyte nuclei (white dashed line) at late stage. Scale bar is 5 μm. (C, D) RNA FISH to visualize Y-loop A/C and *kl-2* and *kl-3* transcripts in control testis. Single spermatocyte nuclei (white dashed line) at late stage. Scale bar is 5 μm. (C) Y-loop A/C (Cy5-(AAGAC)_6_, green), *kl-2* introns (blue), *kl-2* exon 8 (red), and DAPI (white). (D) Y-loop A/C (Cy5-(AAGAC)_6_, green), *kl-3* exon 1 (blue), *kl-3* exons 16–17 (red), and DAPI (white). (E-L) RNA FISH to visualize *kl-2*, *kl-3*, *kl-5*, and *ks-1* transcripts in *maca-EGFP* testis. Single spermatocyte nuclei (white dashed line) at late stage. Scale bar is 5 μm. (E) Maca-EGFP (green), *kl-2* introns (blue), *kl-2* exon 8 (red), and DAPI (white). (F) Maca-EGFP (green), *kl-3* exon 1 (blue), *kl-3* exon 14 (red), and DAPI (white). (G) Maca-EGFP (green), *kl-3* exon 13 (blue), *kl-3* exon 14 (red), and DAPI (white). (H) Maca-EGFP (green), *kl-5* exons 1–6 (blue), *kl-5* exons 16–17 (red), and DAPI (white). (I) Maca-EGFP (green), *kl-5* exons 1–6 (blue), *ks-1* exons all (red), and DAPI (white). (J) Maca-EGFP (green), Y-loop B (*kl-3* introns, Cy5-(AATAT)_6_, blue), *kl-3* exon 14 (red), and DAPI (white). (K) Maca-EGFP (green), *kl-5* exons 1–6 (blue), Y-loop A/C (Cy3-(AAGAC)_6_, red), and DAPI (white). (L) Maca-EGFP (green), *ks-1* exons all (blue), Y-loop A/C (Cy3-(AAGAC)_6_, red), and DAPI (white).
(TIF)

S16 Fig Maca mutation does not affect the localizations of Y-Loop A/C and Y-Loop B in the spermatocyte nucleus and the localization of *ks-1* mRNA in the spermatocyte cytoplasm. (A-D) RNA FISH to visualize Y-loops in (A, C) *maca*^*null/+*^ and (B, D) *maca*^*null/null*^ testes. Y-loop A/C (Cy3-(AAGAC)_6_, red), Y-loop B (*kl-3* introns, Alexa488-(AATAT)_6_, green), and DAPI (blue). (A, B) Apical regions of testes including spermatocyte growth region. The apical tip of testis is marked by *. Scale bar is 50 μm. (C, D) Single spermatocyte nuclei (white dashed line) at late stage. Scale bar is 5 μm. (E, F) RNA FISH to visualize Y-loop A/C and *kl-5* and *ks-1* transcripts in (E) *maca*^*null/+*^ and (F) *maca*^*null/null*^ testes. Y-loop A/C (Alexa488-(AAGAC)_6_, green), *kl-5* exons 1–6 (blue), *ks-1* exons all (red), and DAPI (white). Single spermatocyte nuclei (white dashed line) at late stage. Cytoplasmic *kl-5* and *ks-1* mRNA granules are indicated by cyan and magenta arrows, respectively. Scale bar is 5 μm.
(TIF)

S1 Table List of oligonucleotide primers used in this study. (DOCX)

S2 Table List of RNA FISH probe sequences used in this study. (DOCX)
