# Supplementary figures and images for: RNA-binding protein Maca is crucial for gigantic male fertility factor gene expression, spermatogenesis, and male fertility, in Drosophila
Source: PLoS Genet. 2021 Jun 28;17(6):e1009655. doi: 10.1371/journal.pgen.1009655 (PMC8248703; doi:10.1371/journal.pgen.1009655)

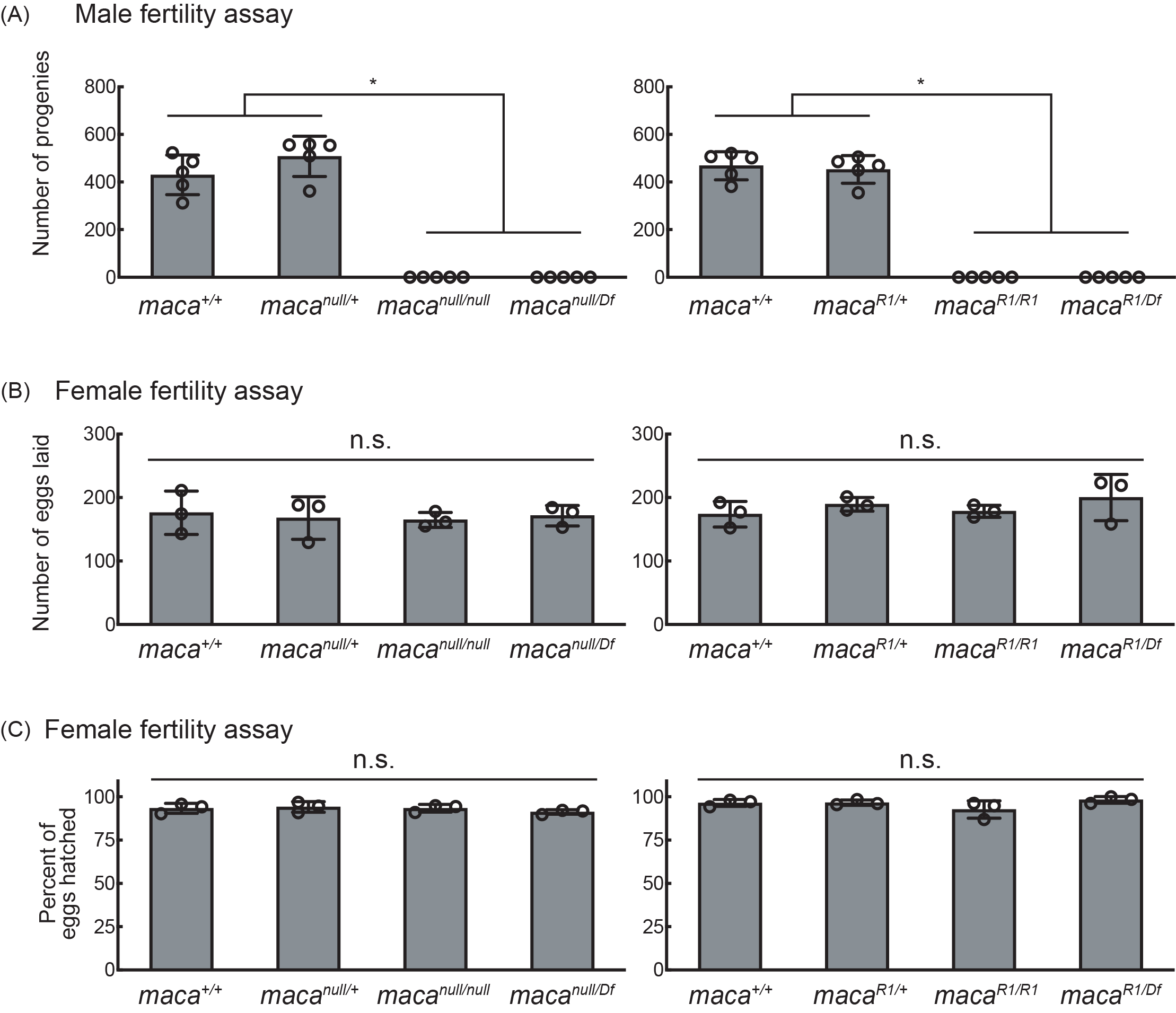

Supplement: S1 Fig — (A) Male fertility assay. The numbers of the progeny flies obtained from the crosses between test males and OregonR wild-type virgin females are shown. Mean +/- SD (n = 5 biological replicates). P-value <0.05 (Student’s t-test) are indicated by *. Trans-heterozygous mutant flies macanull/Df and macaR1/Df have the macanull or macaR1 allele and the Df(3R)Exel6174 chromosomal deficiency allele uncovering the maca gene. (B, C) Female fertility assay. (B) Numbers of eggs laid by test virgin females crossed with OregonR wild-type males and (C) hatching rates from the eggs. Mean +/- SD (n = 3 biological replicates). P-value <0.05 (Student’s t-test) are indicated by *. (TIF) [file pgen.1009655.s001.tif]

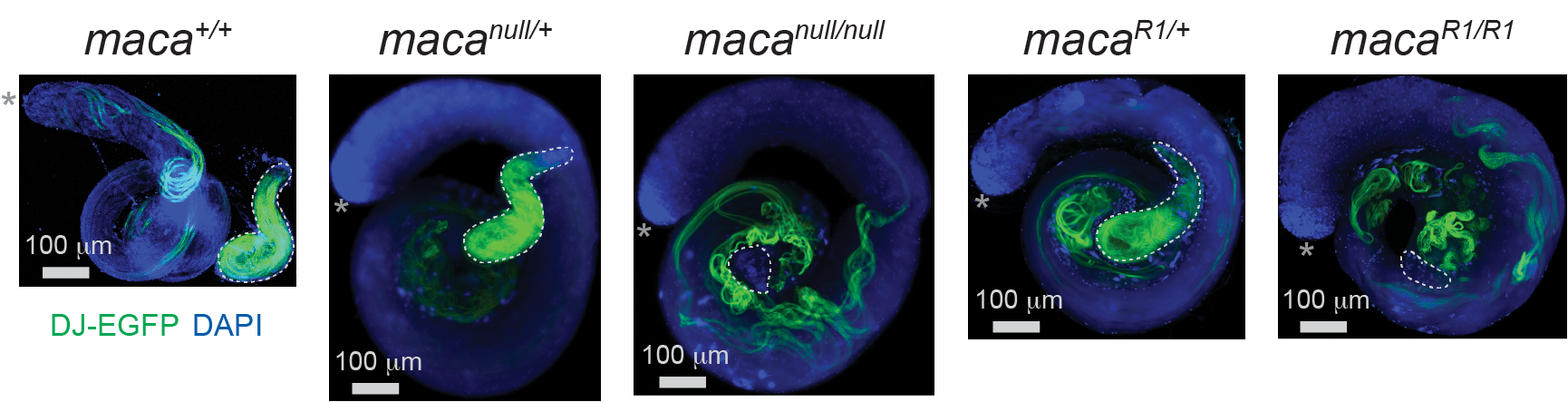

Supplement: S2 Fig — Confocal imaging of whole testes and seminal vesicles from indicated genotypes in the dj-GFP background. DJ-GFP (green), DAPI (blue). DJ-GFP is expressed in sperm. The apical tip of testis is marked by *. Seminal vesicles are indicated by white dashed line. Scale bars are 100 μm. (TIF) [file pgen.1009655.s002.tif]

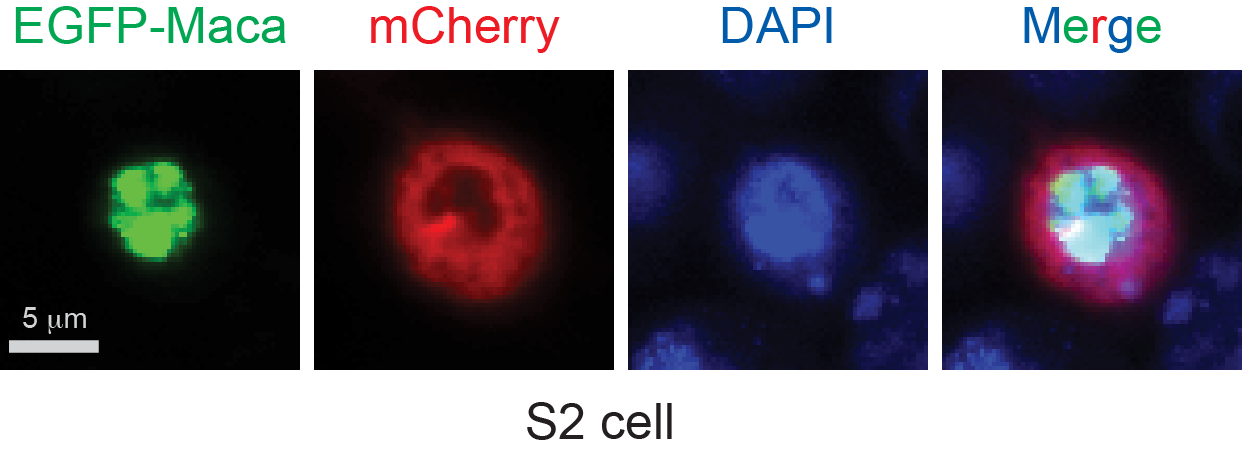

Supplement: S3 Fig — Confocal imaging of S2 cells transiently expressing EGFP-Maca. mCherry signal marks the cytoplasm while DAPI signal marks the nucleus. EGFP-Maca resides in the nucleus. Scale bar is 5 μm. (TIF) [file pgen.1009655.s003.tif]

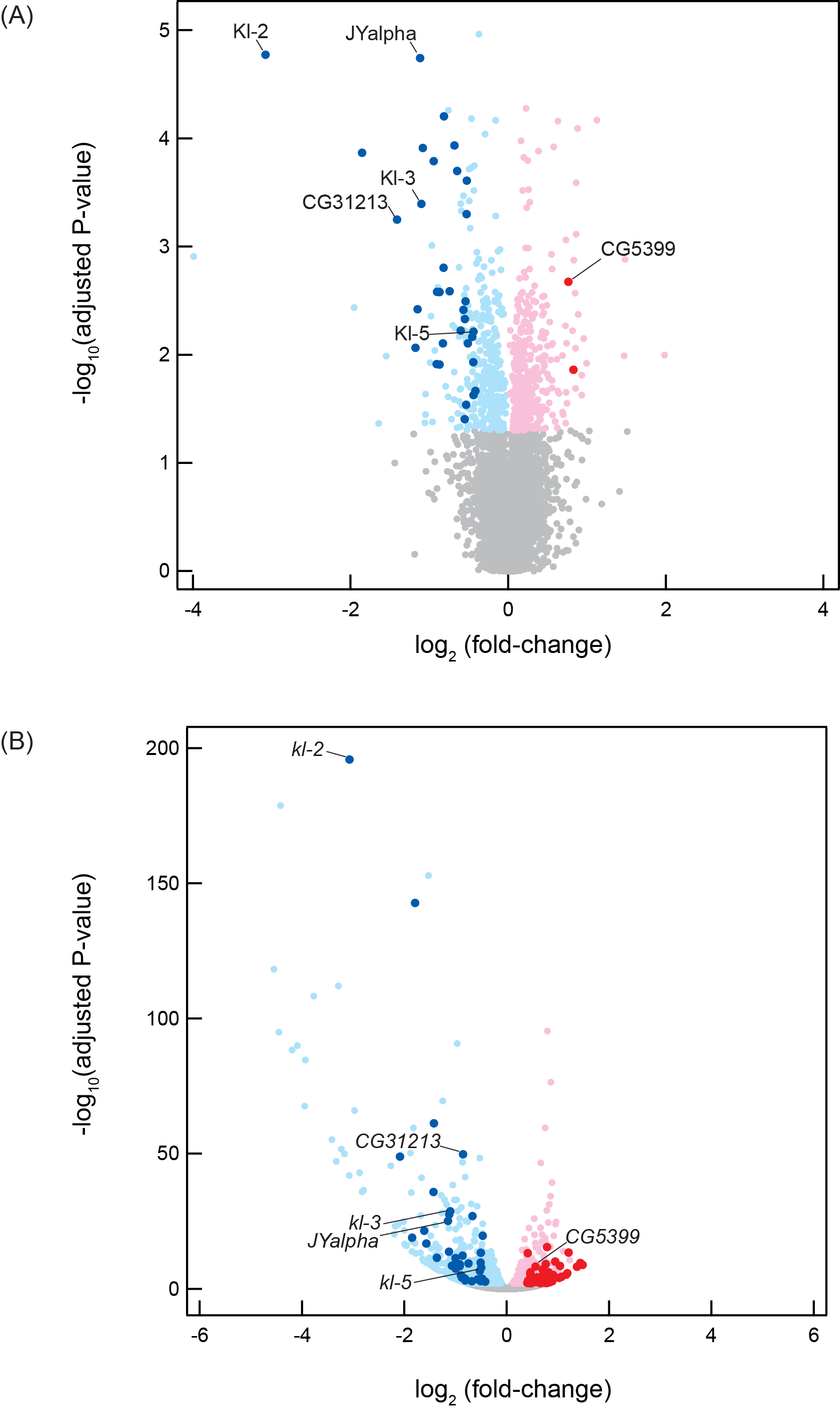

Supplement: S4 Fig — (A) Volcano plot showing log2(fold-change) and -log10(adjusted P-value) of protein abundance in macanull/null testes compared with macanull/+ testes determined by mass-spec. Light blue: Proteins with adjusted P-value (= FDR) <0.05 and fold-change < 1 in macanull/null testes compared with macanull/+. Dark blue: Proteins with adjusted P-value <0.05 and fold-change > 1 in macanull/null testes compared with maca+/+, macanull/+, and macanull/null with maca-EGFP rescue transgene, consistently. Full list shown in Fig 3. Pink: Proteins with adjusted P-value <0.05 and fold-change > 1 in macanull/null testes compared with macanull/+. Red: Proteins with adjusted P-value <0.05 and fold-change > 1 in macanull/null testes compared with maca+/+, macanull/+, and macanull/null with maca-EGFP rescue transgene, consistently. Full list shown in Fig 3. Gray: Proteins with adjusted P-value > = 0.05 and fold-change < 1 in macanull/null testes compared with macanull/+. (B) Volcano plot showing log2(fold-change) and -log10(adjusted P-value) of each mRNA in macanull/null testes compared with macanull/+ testes determined by poly-A+ RNA-seq. Light blue: Proteins with adjusted P-value (= FDR) <0.01 and fold-change < 1 in macanull/null testes compared with macanull/+. Dark blue: Proteins with adjusted P-value <0.01 and fold-change > 1 in macanull/null testes compared with maca+/+, macanull/+, macanull/+ with maca-EGFP rescue transgene, and macanull/null with maca-EGFP rescue transgene, consistently. Full list shown in Fig 4B. Pink: Proteins with adjusted P-value <0.01 and fold-change > 1 in macanull/null testes compared with macanull/+. Red: Proteins with adjusted P-value <0.01 and fold-change > 1 in macanull/null testes compared with maca+/+, macanull/+, macanull/+ with maca-EGFP rescue transgene, and macanull/null with maca-EGFP rescue transgene, consistently. Full list shown in Fig 4A. Gray: Proteins with adjusted P-value > = 0.01 and fold-change < 1 in macanull/null testes compared with maca [file pgen.1009655.s004.tif]

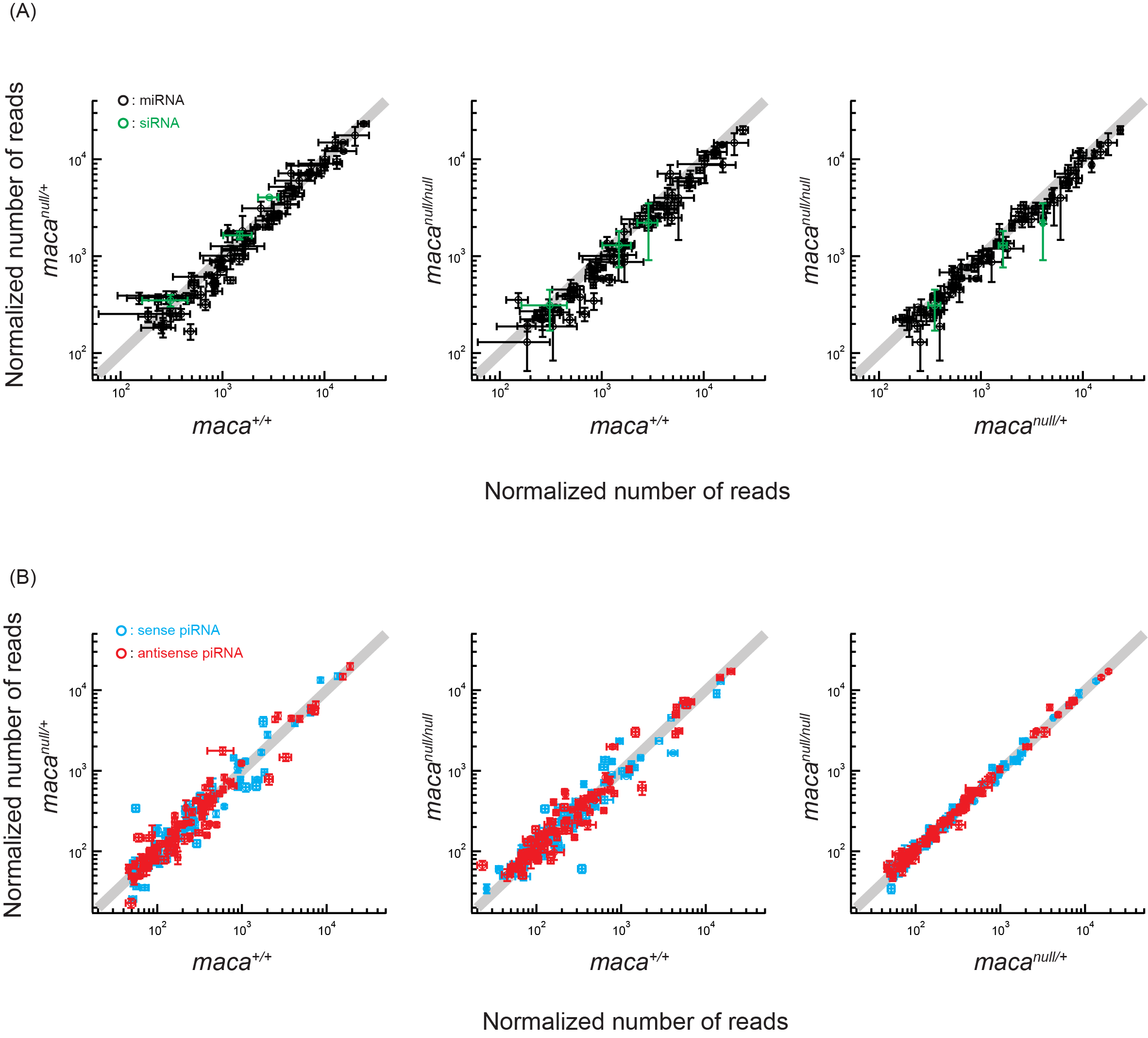

Supplement: S5 Fig — Scattered plots of normalized number of reads (reads per million non-rRNA-mapping genome-mapping reads) of (A) miRNAs and endo-siRNA (esi-1.1, esi-1.2, and esi-2.1) and (B) sense and antisense piRNAs in testis small RNA-seq. Each dot represents a unique miRNA, endo-siRNA, or piRNA cluster (transposon-sense or transposon-antisense mapping). Mean +/- SD (n = 3 biological replicates). (TIF) [file pgen.1009655.s005.tif]

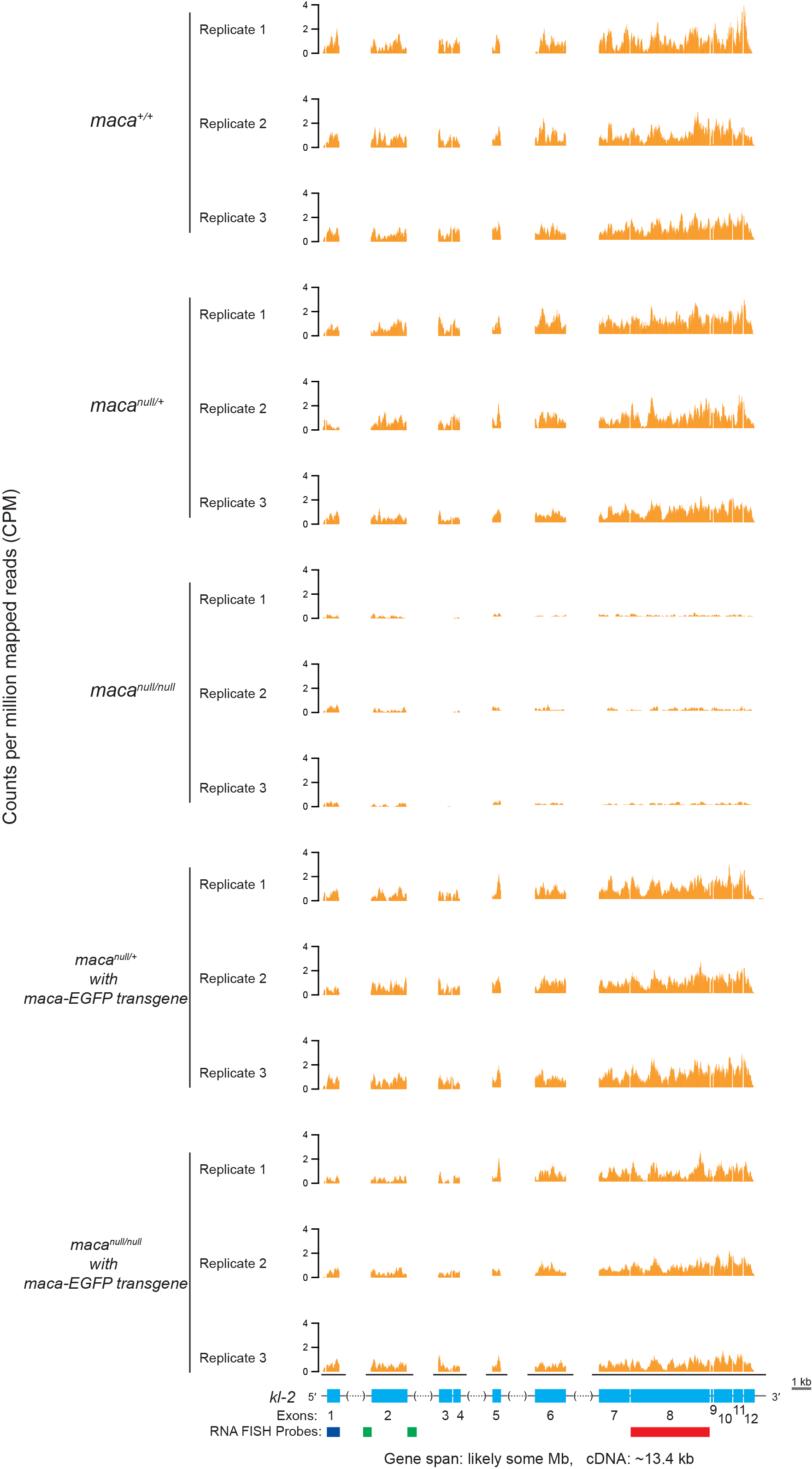

Supplement: S6 Fig — Poly-A+ RNA-seq normalized read counts (counts per million mapped reads, CPM) in the kl-2 gene region. Gene structures are shown with exons (cyan), introns (black line), intronic satellite DNA repeats (dashed line in parentheses). Regions targeted by RNA FISH probes used in Figs 8, S10 and S15 (green and red). (TIF) [file pgen.1009655.s006.tif]

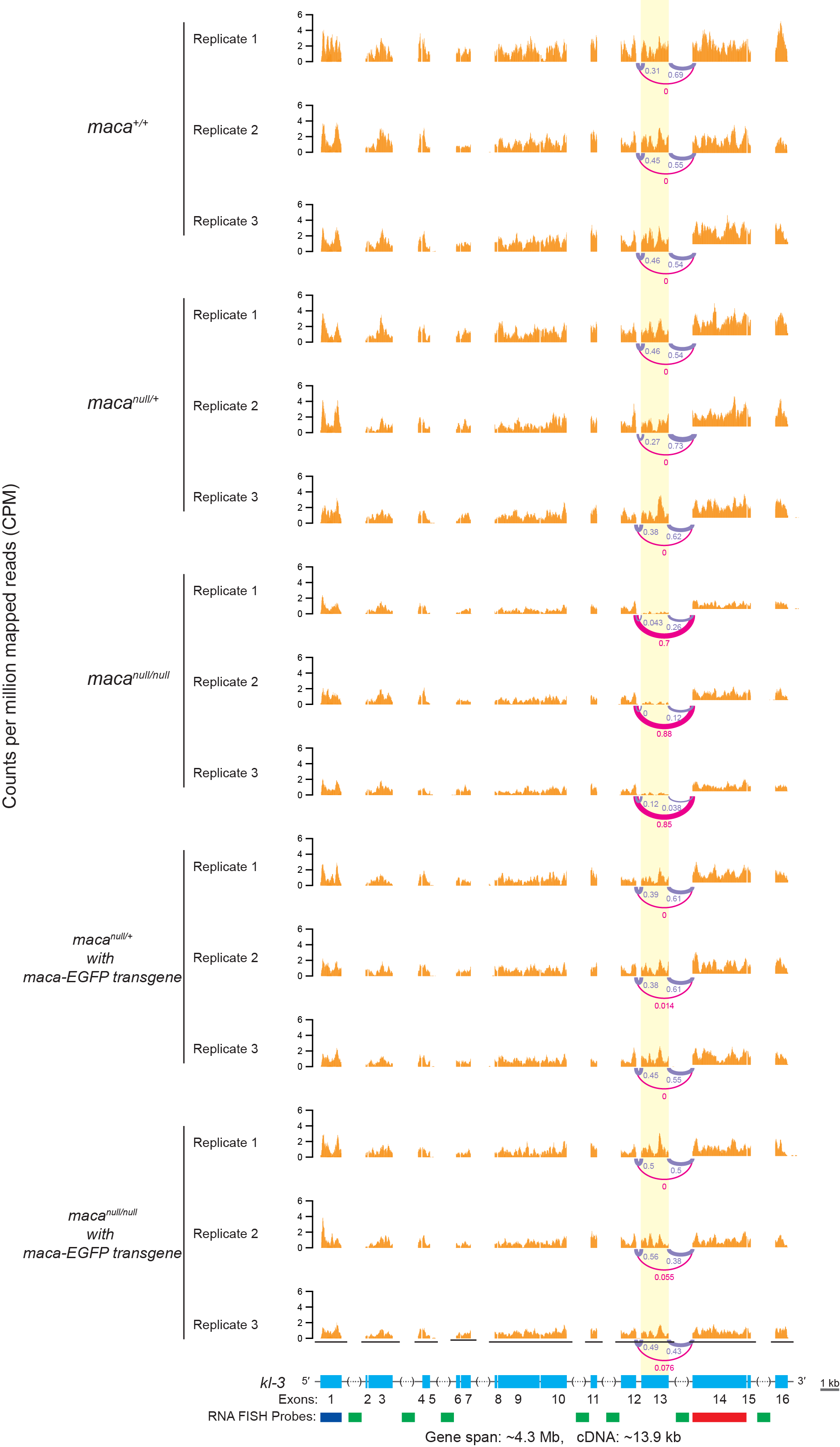

Supplement: S7 Fig — Poly-A+ RNA-seq normalized read counts (counts per million mapped reads, CPM) in the kl-3 gene region. Splicing ratios among (1) exon 12—exon 13, (2) exon 13—exon 14, and (3) exon 12—exon 14 (= exon 13-skipped) in determined by LeafCutter are shown. Gene structures are shown with exons (cyan), introns (black line), intronic satellite DNA repeats (dashed line in parentheses). Regions targeted by RNA FISH probes used in Figs 10, S9, S13 and S15 (blue, green, and red). (TIF) [file pgen.1009655.s007.tif]

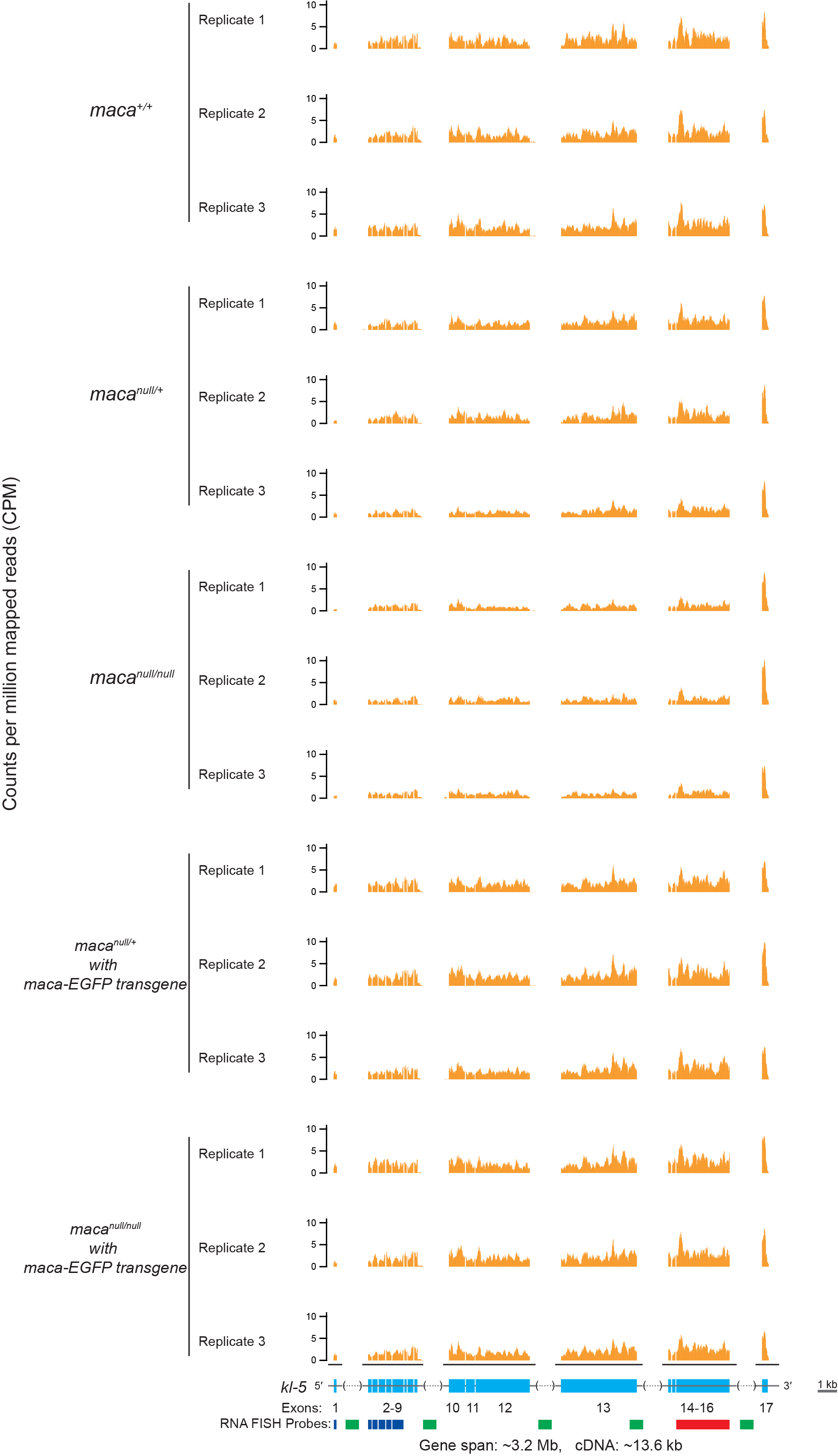

Supplement: S8 Fig — Poly-A+ RNA-seq normalized read counts (counts per million mapped reads, CPM) in the kl-5 gene region. Gene structures are shown with exons (cyan), introns (black line), intronic satellite DNA repeats (dashed line in parentheses). Regions targeted by RNA FISH probes used in S9, S14, S15 and S16 Figs (blue, green, and red). (TIF) [file pgen.1009655.s008.tif]

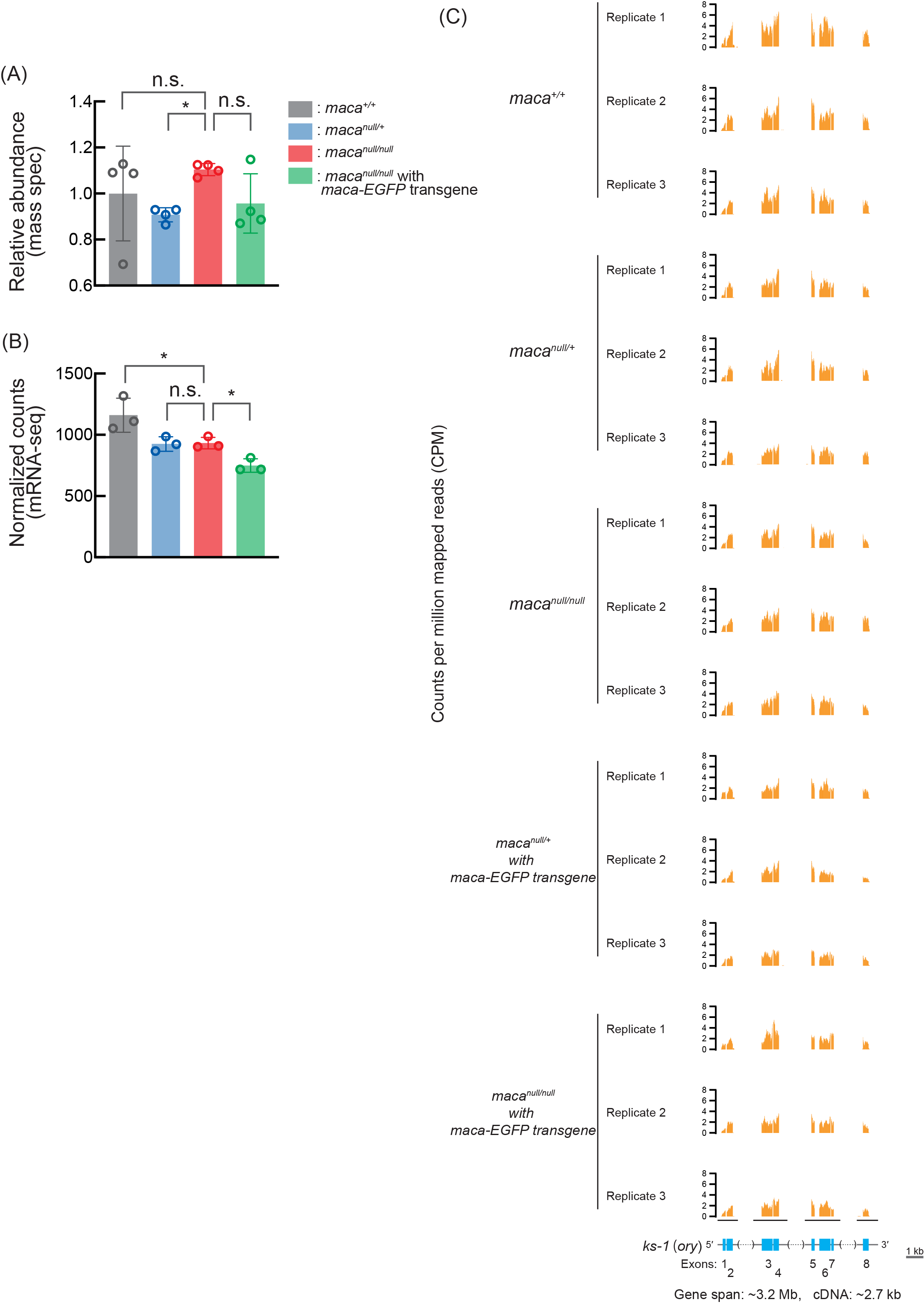

Supplement: S9 Fig — (A) Relative abundance of Ks-1 (ORY) protein in testes determined by mass spec with TMT labeling. Mean +/- SD (n = 4 biological replicates). Adjusted P-values <0.05 are indicated by *. (B) Normalized counts (reflecting relative abundance) of ks-1 (ory) mRNA determined by poly-A+ RNA-seq. Mean +/- SD (n = 3 biological replicates). Adjusted P-values <0.01 are indicated by *. (C) poly-A+ RNA-seq normalized read counts (counts per million mapped reads, CPM) in the ks-1 (ory) gene region. Gene structures are shown with exons (cyan), introns (black line), intronic satellite DNA repeats (dashed line in parentheses). (TIF) [file pgen.1009655.s009.tif]

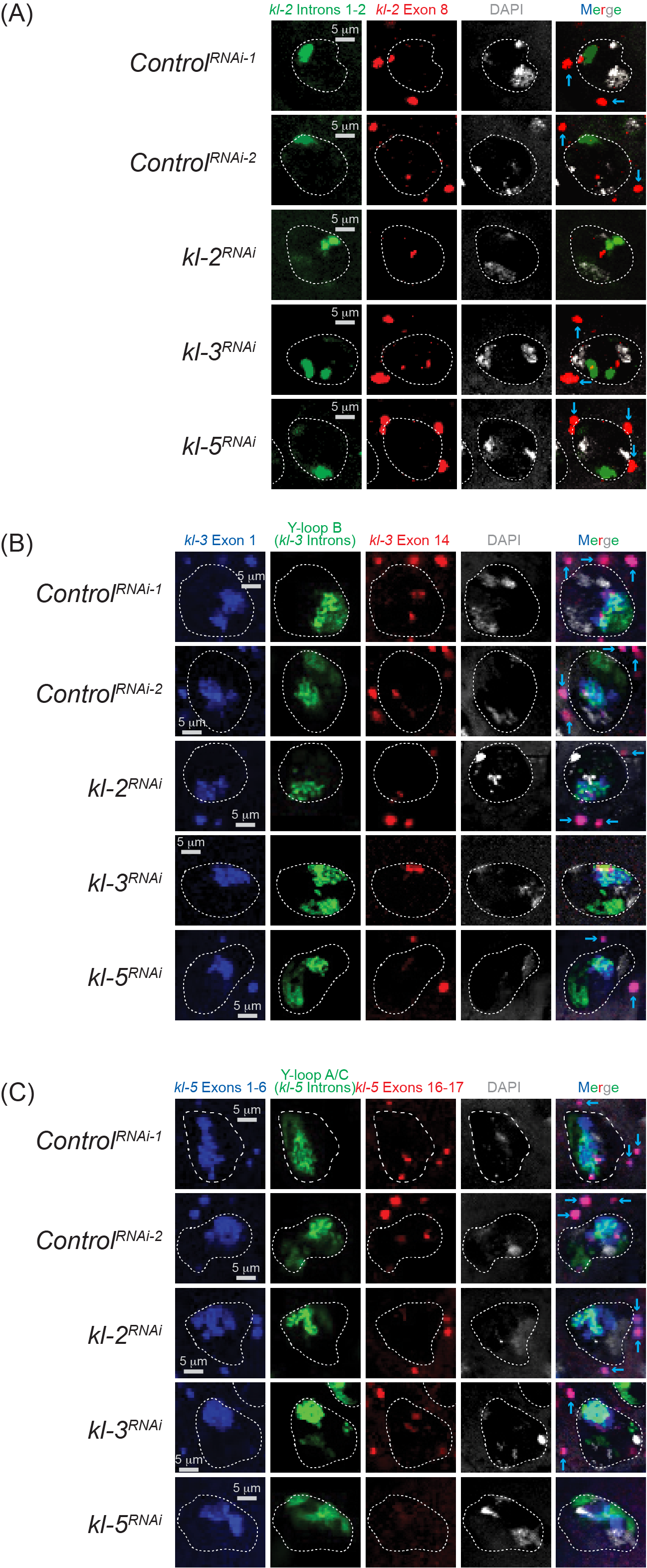

Supplement: S10 Fig — RNA FISH to visualize (A) kl-2, (B) kl-3, and (C) kl-5 transcripts in late-stage spermatocytes. Single spermatocyte nuclei (white dashed line) at stage 4 of their transcript expression. Scale bars are 5 μm. Cytoplasmic mRNA granules (kl-granules) are indicated by cyan arrows. (A) kl-2 introns 1–2 (green), exon 8 (red), and DAPI (white). (B) kl-3 exon 1 (blue), introns (Y-loop B, Alexa488-(AATAT)6, green), exon 14 (red), and DAPI (white). (C) kl-5 exons 1–6 (blue), introns (Y-loop A/C, Alexa488-(AAGAC)6, green), exons 16–17 (red), and DAPI (white). (TIF) [file pgen.1009655.s010.tif]

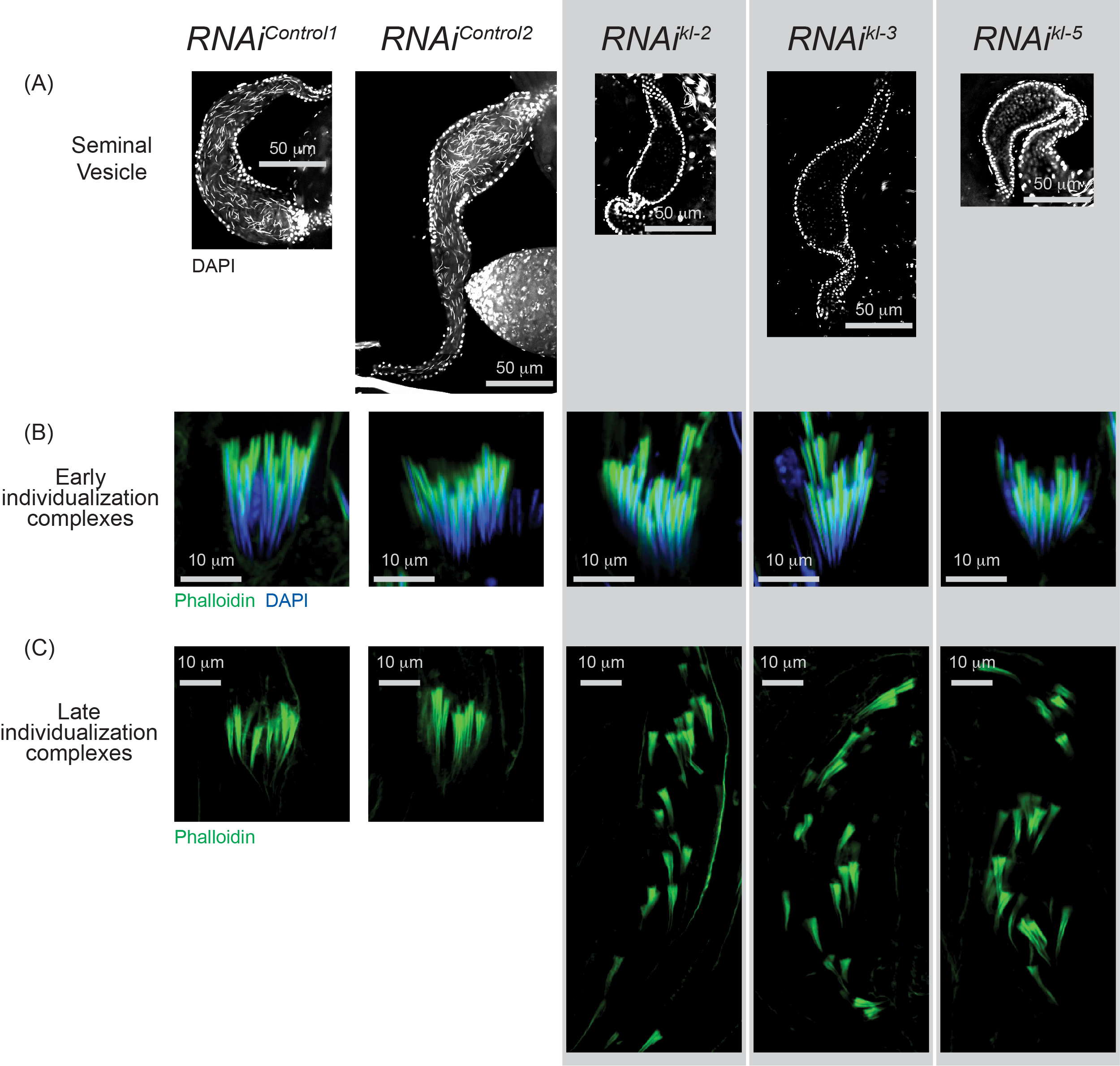

Supplement: S11 Fig — (A) Confocal images of dissected seminal vesicles stained with DAPI. Motile sperm is absent in RNAikl-2, RNAikl-3, and RNAikl-5. Scale bars are 50 μm. (B) Confocal images of the early-stage individualization complexes. Phalloidin (Actin, green), DAPI (blue). Scale bars are 10 μm. (C) Confocal images of the late-stage individualization complexes. Phalloidin (Actin, green). Scale bars are 10 μm. (TIF) [file pgen.1009655.s011.tif]

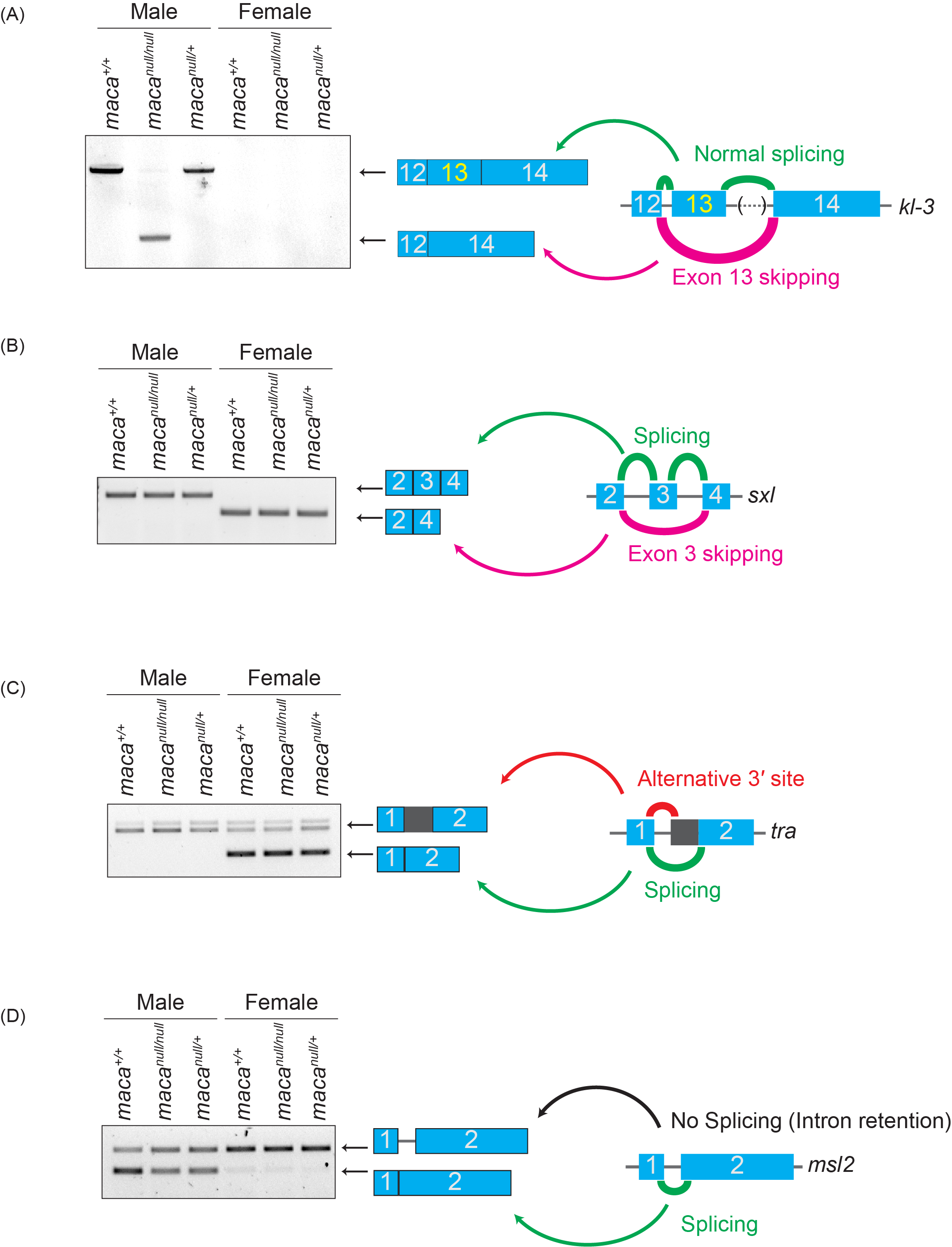

Supplement: S12 Fig — Agarose gel electrophoresis of RT-PCR products to test (A) kl-3 mRNA exon 13 skipping and alternative splicing of (B) sxl, (C) tra, and (D) msl2 mRNAs. Whole fly total RNAs were used for RT-PCR. (TIF) [file pgen.1009655.s012.tif]

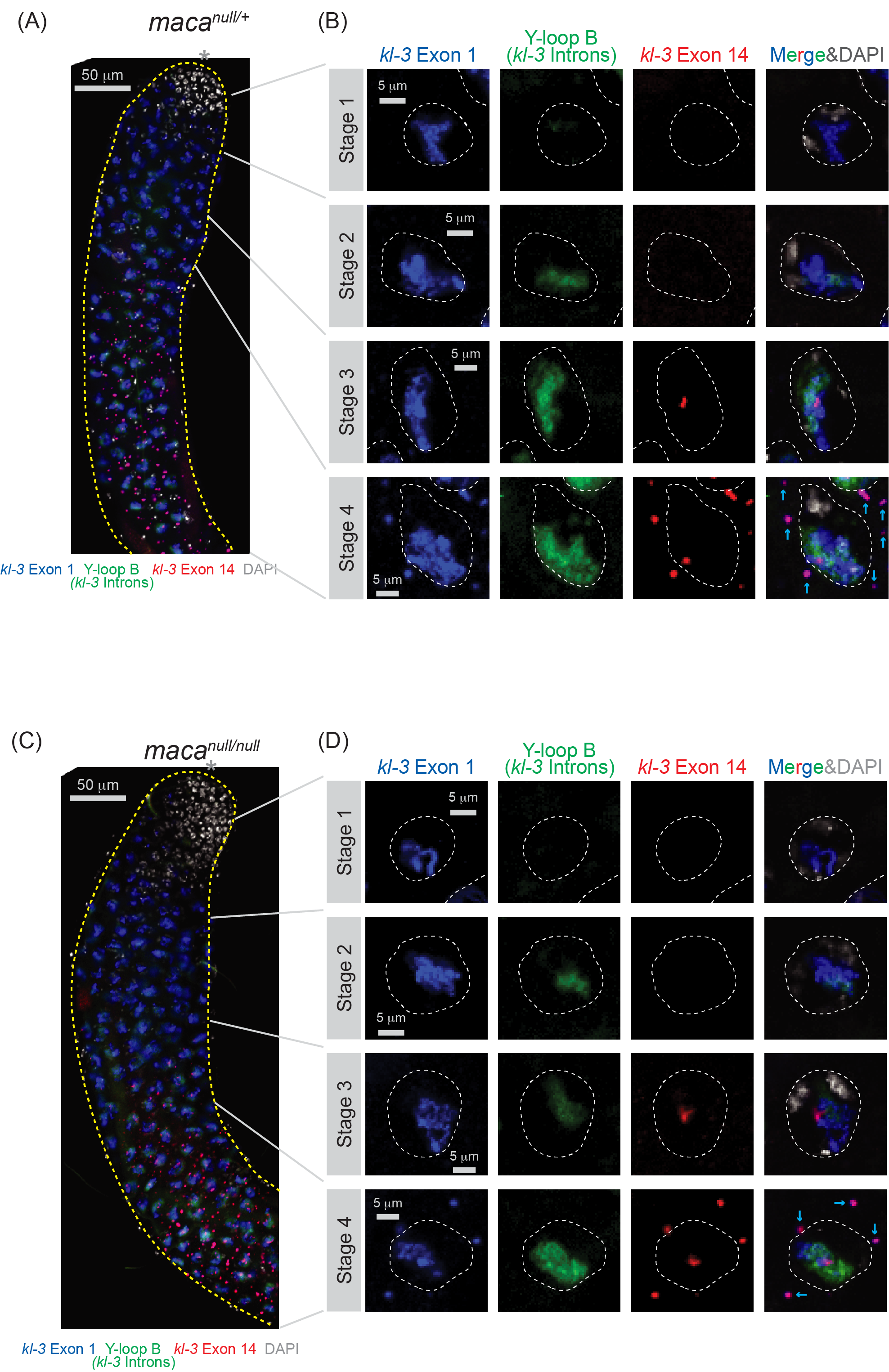

Supplement: S13 Fig — RNA FISH to visualize kl-3 transcripts expression in (A, B) macanull/+ and (C, D) macanull/null testes. Exon 1 (blue), Y-loop B (kl-3 introns, Alexa488-(AATAT)6, green), exon 14 (red), and DAPI (white). (A, C) Apical regions of testes including the spermatocyte growth region are shown. The apical tip of testis is marked by *. Scale bars are 50 μm. (B, D) Single spermatocyte nuclei (white dashed line) at each stage of kl-3 expression. Cytoplasmic mRNA granules are indicated by cyan arrows. Scale bars are 5 μm. (TIF) [file pgen.1009655.s013.tif]

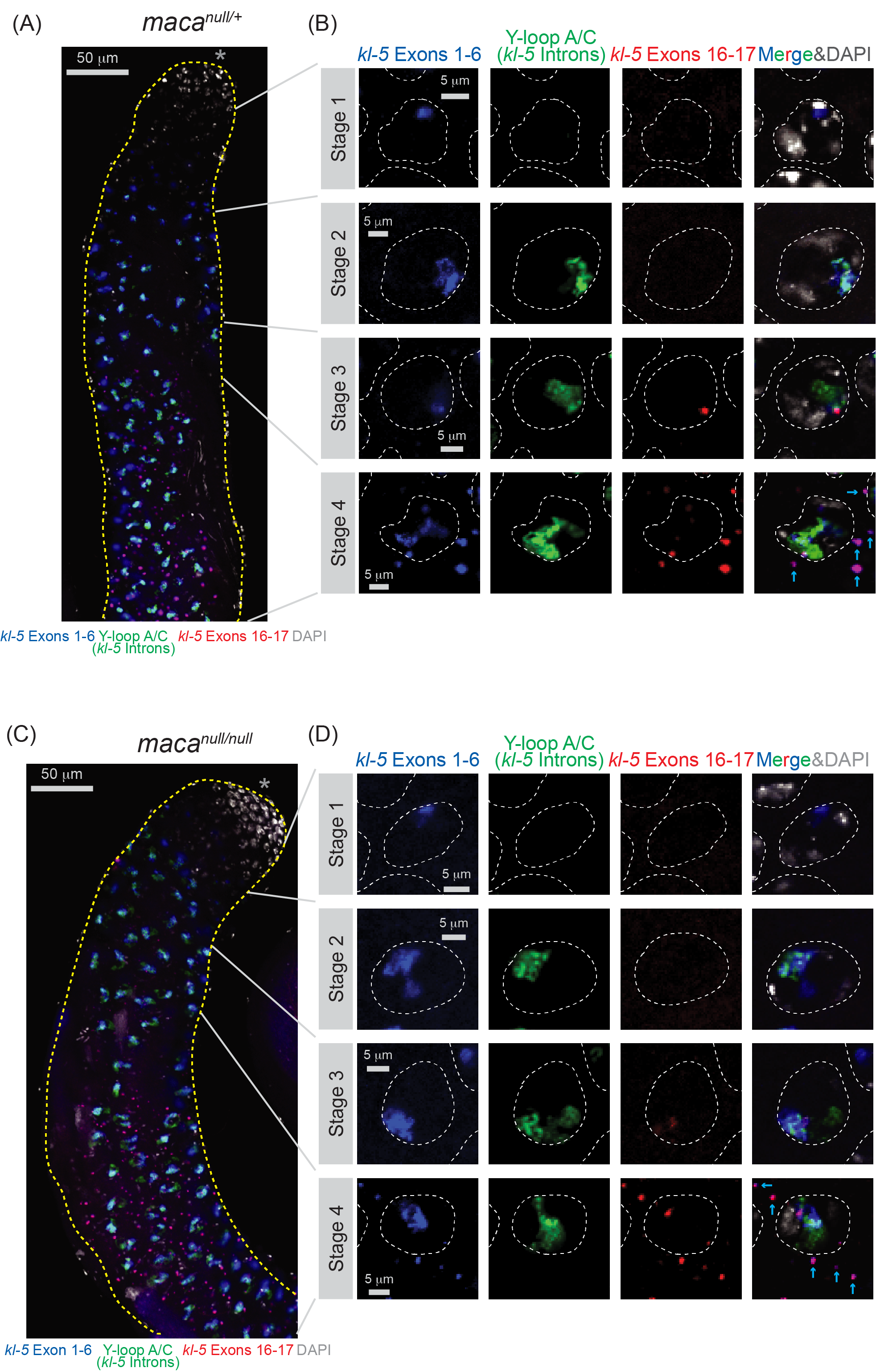

Supplement: S14 Fig — RNA FISH to visualize kl-5 transcripts expression in (A, B) macanull/+ and (C, D) macanull/null testes. Exons 1–6 (blue), Y-loop A/C (kl-5 introns, Alexa488-(AAGAC)6, green), exons 16–17 (red), and DAPI (white). (A, C) Apical regions of testes including the spermatocyte growth region are shown. The apical tip of testis is marked by *. Scale bars are 50 μm. (B, D) Single spermatocyte nuclei (white dashed line) at each stage of kl-5 expression. Cytoplasmic mRNA granules are indicated by cyan arrows. Scale bars are 5 μm. (TIF) [file pgen.1009655.s014.tif]

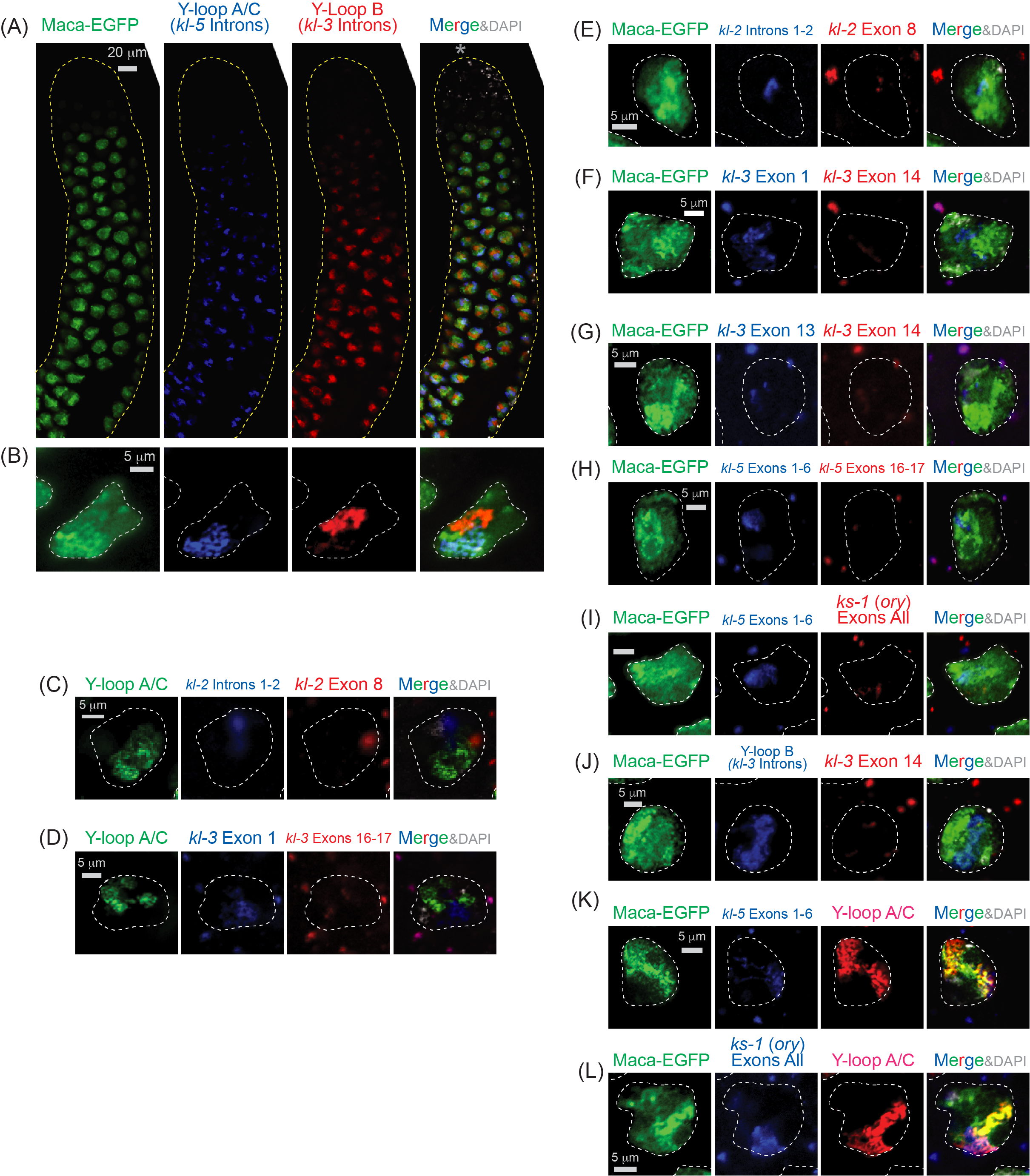

Supplement: S15 Fig — (A, B) RNA FISH to visualize Y-loops in maca-EGFP testis. Maca-EGFP (green), Y-loop A/C (Cy5-(AAGAC)6, blue), Y-loop B (kl-3 introns, Cy3-AATAT)6, red), and DAPI (white). (A) Apical regions of testes including spermatocyte growth region are shown. The apical tip of testis is marked by *. Scale bar is 20 μm. (B) Single spermatocyte nuclei (white dashed line) at late stage. Scale bar is 5 μm. (C, D) RNA FISH to visualize Y-loop A/C and kl-2 and kl-3 transcripts in control testis. Single spermatocyte nuclei (white dashed line) at late stage. Scale bar is 5 μm. (C) Y-loop A/C (Cy5-(AAGAC)6, green), kl-2 introns (blue), kl-2 exon 8 (red), and DAPI (white). (D) Y-loop A/C (Cy5-(AAGAC)6, green), kl-3 exon 1 (blue), kl-3 exons 16–17 (red), and DAPI (white). (E-L) RNA FISH to visualize kl-2, kl-3, kl-5, and ks-1 transcripts in maca-EGFP testis. Single spermatocyte nuclei (white dashed line) at late stage. Scale bar is 5 μm. (E) Maca-EGFP (green), kl-2 introns (blue), kl-2 exon 8 (red), and DAPI (white). (F) Maca-EGFP (green), kl-3 exon 1 (blue), kl-3 exon 14 (red), and DAPI (white). (G) Maca-EGFP (green), kl-3 exon 13 (blue), kl-3 exon 14 (red), and DAPI (white). (H) Maca-EGFP (green), kl-5 exons 1–6 (blue), kl-5 exons 16–17 (red), and DAPI (white). (I) Maca-EGFP (green), kl-5 exons 1–6 (blue), ks-1 exons all (red), and DAPI (white). (J) Maca-EGFP (green), Y-loop B (kl-3 introns, Cy5-(AATAT)6, blue), kl-3 exon 14 (red), and DAPI (white). (K) Maca-EGFP (green), kl-5 exons 1–6 (blue), Y-loop A/C (Cy3-(AAGAC)6, red), and DAPI (white). (L) Maca-EGFP (green), ks-1 exons all (blue), Y-loop A/C (Cy3-(AAGAC)6, red), and DAPI (white). (TIF) [file pgen.1009655.s015.tif]

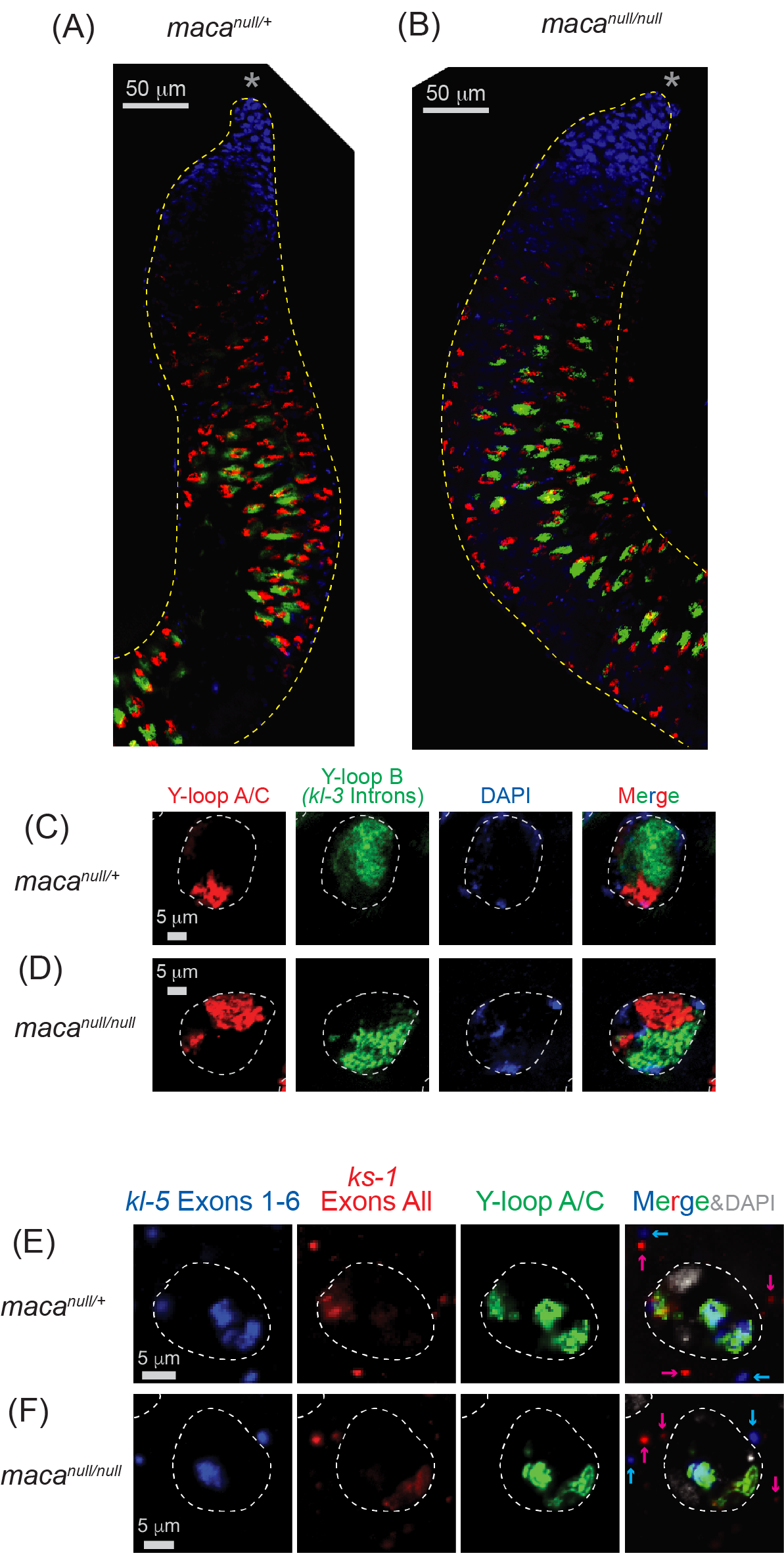

Supplement: S16 Fig — (A-D) RNA FISH to visualize Y-loops in (A, C) macanull/+ and (B, D) macanull/null testes. Y-loop A/C (Cy3-(AAGAC)6, red), Y-loop B (kl-3 introns, Alexa488-(AATAT)6, green), and DAPI (blue). (A, B) Apical regions of testes including spermatocyte growth region. The apical tip of testis is marked by *. Scale bar is 50 μm. (C, D) Single spermatocyte nuclei (white dashed line) at late stage. Scale bar is 5 μm. (E, F) RNA FISH to visualize Y-loop A/C and kl-5 and ks-1 transcripts in (E) macanull/+ and (F) macanull/null testes. Y-loop A/C (Alexa488-(AAGAC)6, green), kl-5 exons 1–6 (blue), ks-1 exons all (red), and DAPI (white). Single spermatocyte nuclei (white dashed line) at late stage. Cytoplasmic kl-5 and ks-1 mRNA granules are indicated by cyan and magenta arrows, respectively. Scale bar is 5 μm. (TIF) [file pgen.1009655.s016.tif]
