# Supplementary material for: RNA-binding protein Maca is crucial for gigantic male fertility factor gene expression, spermatogenesis, and male fertility, in Drosophila
Source: PLoS Genet. 2021 Jun 28;17(6):e1009655. doi: 10.1371/journal.pgen.1009655 (PMC8248703; doi:10.1371/journal.pgen.1009655)
Supplement: S1 Table — (DOCX) [file pgen.1009655.s017.docx]

**Supplementary Table 1.**

List of oligonucleotide primers

| RT-PCR splicing assay | |
| --- | --- |
| Name | Sequence (5′ - 3′) |
| *kl-2*-F | TTCTCAAAGTCAAACACGAAGAGTG |
| *kl-2*-R | TTTTCCCCAGCTAAACCACCAATAA |
| *sxl*-F | CTCACCTTCGATCGAGGGTGTA |
| *sxl*-R | GATGGCAGAGAATGGGAC |
| *msl2*-F | CACTGCGGTCACACTGGCTTCGCTCAG |
| *msl2*-R | CTCCTGGGCTAGTTACCTGCAATTCCTC |
| *tra*-F | GGATGCCGACAGCAGTGGAAC |
| *tra*-R | GATCTGGAGCGAGTGCGTCTG |
| qRT-PCR | |
| *actin5C*_F | ﻿AAGTTGCTGCTCTGGTTGTCG |
| *actin5C*_R | ﻿GCCACACGCAGCTCATTGTAG |
| *kl-2*_amplicon1_F (exon1) | CGCGACTGGAATGCAAAAGT |
| *kl-2*_amplicon1_R | GCAACCAGTGAGAACATCGC |
| *kl-2*_amplicon2_F (exon1-exon2) | TCGTGGGCTACGCTTATTCG |
| *kl-2*_amplicon2_R | TCGTGGGCTACGCTTATTCG |
| *kl-2*_amplicon3_F (exon2-exon3) | TCCAGTTGCGCATTTAGTCCT |
| *kl-2*_amplicon3_R | GGCGGTAATAGAAGATatgccca |
| *kl-2*_amplicon4_F (exon4-exon5) | CTGCCATATCTGGGTCACGG |
| *kl-2*_amplicon4_R | CCAACCTCTGCTAACATaaatcca |
| *kl-2*_amplicon5_F (exon6-exon7) | CTGCGAGGTCTCTCATCAGG |
| *kl-2*_amplicon5_R | CTGGGGATGTTTCGATGAGttta |
| *kl-2*_amplicon6_F (exon8-exon9-exon10) | CGAACATAAGCTCCAACGCC |
| *kl-2*_amplicon6_R | ACCATggtggataagtgagca |
| *kl-2*_amplicon7_F (intron1) | ACTGTGTTTGTGAGAGTCGAAAG |
| *kl-2*_amplicon7_R | TATAACATGCATGGGAGAGGAGG |
| *kl-2*_amplicon8_F (intron1-exon2) | AAAATCGGTCAAGGCGTTTCAG |
| *kl-2*_amplicon8_R | TCCAGTTGCGCATTTAGTCCT |
| *kl-2*_amplicon9_F (exon2-intron2) | GCAGTTTTTAAAGAATTTCAGGCGG |
| *kl-2*_amplicon9_R | CCTCGACTGAGATACTATCCTGG |
| *kl-2*_amplicon10_F (intron2) | CCTCTGAAGGATTGGTTCGATCA |
| *kl-2*_amplicon10_R | GTTTTTAACCAGGGCAACAGACA |
| *kl-2*_amplicon11_F (intron6-exon7) | CTGGGGATGTTTCGATGAGTT |
| *kl-2*_amplicon11_R | CGAACATAAGCTCCAACGCC |
| *kl-3*_amplicon1_F (exon1-exon2) | CCCGAGCATTTAATAACCACAAG |
| *kl-3*_amplicon1_R | AACGGACATTATCCTTAGCTTCA |
| *kl-3*_amplicon2_F (exon2-exon3) | TTGGGATCCCTTATACCGttcttc |
| *kl-3*_amplicon2_R | CCATAAGACCTGTAACGTTGACAG |
| *kl-3*_amplicon3_F (exon5-exon6) | GCTGGATCTAAGAGGTCATTGG |
| *kl-3*_amplicon3_R | GGCTGAATGTAACACCCGTTAT |
| *kl-3*_amplicon4_F (exon6-exon7) | GGCGTGTTACTGTCGatgaa |
| *kl-3*_amplicon4_R | CACGCTGAAATTCTTCCATGTC |
| *kl-3*_amplicon5_F (exon13-exon14) | AGTTCTTGGGACGAATCCCTT |
| *kl-3*_amplicon5_R | GCCTCAAATGTGTATTGCGGAT |
| *kl-3*_amplicon6_F (exon14-exon15) | TATGTCCATTCAACCTAAAGaatcgtc |
| *kl-3*_amplicon6_R | CCCATTGCAATTAGATGCTGTT |
| *kl-3*_amplicon7_F (exon15-exon16) | GCCACGAGCTCGATGAATA |
| *kl-3*_amplicon7_R | AGTACCTTCAACGGCAAGAA |
| *kl-5*_amplicon1_F (exon1-exon2) | ATGCGTCTTAAGCTGGATAAGT |
| *kl-5*_amplicon1_R | TGTCCACCGGAATTGATTGT |
| *kl-5*_amplicon2_F (exon7-exon8) | CACGAACTTTACGAATATCCacttt |
| *kl-5*_amplicon2_R | CCTGCCAGCACTCAACA |
| *kl-5*_amplicon3_F (exon10-exon11) | TGAATCCTTACAGCTTCTATGatgag |
| *kl-5*_amplicon3_R | TTTGCCATGGACACGCA |
| *kl-5*_amplicon4_F (exon12-exon13) | CGAGCAATCAAATCGGTTCTTG |
| *kl-5*_amplicon4_R | ACACTGGTACATCATCGGTAAC |
| *kl-5*_amplicon5_F (exon15-exon16) | GACCCTCAACTtcagggaataaa |
| *kl-5*_amplicon5_R | AGTAATACACTTCCATTACTGACTG |
| *kl-5*_amplicon6_F (exon16-exon17) | GCCTCTCGATAGAATGTGTCTT |
| *kl-5*_amplicon6_R | TTTCATGTCCCATCGTGCT |
