# Supplementary material for: RNA-binding protein Maca is crucial for gigantic male fertility factor gene expression, spermatogenesis, and male fertility, in Drosophila
Source: PLoS Genet. 2021 Jun 28;17(6):e1009655. doi: 10.1371/journal.pgen.1009655 (PMC8248703; doi:10.1371/journal.pgen.1009655)
Supplement: S2 Table — (DOCX) [file pgen.1009655.s018.docx]

**Supplementary Table 2.**

List of RNA FISH probe sequences.

| Probes to detect satellite repeat RNAs | | |
| --- | --- | --- |
| Probe Target | Dye | Sequence (5′ - 3′) |
| (AAUAU)n  [Y-loop B] | Alexa488, Cy3,  Cy5 | ATATTATATTATATTATATTATATTATATT |
| (UUGUC)n  [Y-loop A/C] | Alexa488, Cy3,  Cy5 | AAGACAAGACAAGACAAGACAAGACAAGAC |
| **Stellaris RNA FISH probe sets** | | |
| *kl-2*, Introns1-2 | Quasar® 670 | TTTACCTTGGGTTTTATTGA, GTCCGACAAAGACACACGTA, GTAAGTGTATCCCCCAGAAG, TGCCTTGCGTATGTACAGAT, TTTACATCCTTAGGGGATCA, CCTTTCTCAAGCCAGTAAAG, CTCTCTTTCGACTCTCACAA, CAGGGGGACCTAATTTGATG, CAGAAGAAGAAACGGAGCCA, AATAGTTCTTCTCAGGACCT, GTGTCTAATTTTCCGCTGAA, ATTTTGGCGGAGGAGTAGTG, GGGGTTTTACCACTTTTTTC, AGTAATGCTCGAAACTAGCA, CGGTCTAATTTGAGTCGCAG, CCCCTACATTTTTTTTATTC, CCTAAATGTTAGGGGTAGTC, CGTCCAACATCGATTTTTCT, TTTTGTGCTTATCCGGTATA, AAACGCCTTGACCGATTTTG, GTTTTCAAAACGCGACACCT, AGCTGCAGTAATTTTACCTA, CTGGCCAGAGAACTTTTTTT, TTTTTTGTATAGGTTCCCTG, ACCCCCTTACGTATATATAC, TCCTTCGCAAATTTCTAACT, GAGCGTCATTCATCTCATAC, ATTATTTCCTTCTATCGCAG, GATCGAACCAATCCTTCAGA, AAAACAACCGCGATCGGCAG, CAGGGAAATTCATTACCCTT, AGTTTTTAACCAGGGCAACA, ATCGCTAGTGCTAGTGCTAG, GTAGCAATCCACATGTTTCG, TTTCGACTAGCAAGGCGTTG, GGACAGAAGAAGGGATGGGA, GGAACTAAACAAGCGTCTGA, GCGATTGCACGAATTGTCTG, GAACGTTCGTCTATCACTGG, CAGCTTGAGGAAGGGGTTGG, GCTAACTTGAGATGGTGGTT, TTAGCAACCAAAAAGACCCT, GATTGGGAAATCTGCCAACT, CAACGAGTGACGGGCATAAT, ATCCGTATGCAGGTATTTTT, AGATTTAGATTGTGCACGCG, ACGGTTGATAATCTATCCCA, TCTCTCATTGACCATCAAGT |
| *kl-2*, Exon 8 | Quasar® 570 | AGTGTCTCCAGTAGGAACAA, TTCCAACAAGCATCACAGGA, TATAGCCGTTGACGTTTTTC, TTATCACAAGCCTCCATTAC, TTGCGTTTTTGTACGTTTCT, AAAGGTGGCTGAGATCCATA, GTAATCTATCCATTGCCGAA, CCATCGCAGCCATTAATAAT, AACCGACTTTGAGTTCGACT, AGCATCGTTCCAAATATGCG, CTCACGAACTTCATTTGGGT, CAAAGGGTTATAGGTAGCCA, TTTGGCGTCGGTAACATTTT, TCTACCTGTAGATCTTCGTA, CGCGGTTGGGAAATAACTCT, TTAATACTTGTCGGCCTGAT, TCGAAAGTCTCCTGTTTTGT, GAGACTTCTGCTATTTGGTC, ATTTCTCCAGTACTTAGCAT, CTTCAGTTGTTAGCAGAACC, TTTTCTCCGATTGGGCTAAA, TTCCAAAAGGGCTTCTTGTG, GCGTTGAAGAATGGCTTCTG, TGAAAGCCCATTGCGTAATC, CTTGTTCAGAGCTAGCTTTA, CATCCCTTCTAATAAGCACG, TACAGCTTCCATAACCTTTT, ATGTTGGTTCTTTTCCAAGT, AAGCGATACAACAGCCACTT, CTAAAGCAGCTTGCTTTTCT, ACAACCTGAAGCTCTTCGAG, TTCTAAGTCGTTCTTCCTTG, CTCTCCAGAAAGCGATTCTA, AAAAGCCCCTAAGTACGACA, ATACGTAACCTTAAGCTCCA, GTAAGTAATCTGCCATACCG, GCAAACCTTCTTTTAGAGCT, CCCACGTTTTGCAACAATAC, ACCTTTCTCCACTTTGAATG, CGGTGGGTAATGTGGATTTG, CCATCTTGCTTTAGTGCAAA, GTCTTTTTGTTCTTCTAGGG, CGTTTGTTTCGAGCTATTGT, ACTGATGTCTGACGGGATTT, TTTCTACTTCAGTTACCTCG, CGAGGACTTCGCTCAATAGA, TATTTCGGTAGACCGCATAG, GTAGCTTATGTCGCTCGAAA |
| *kl-3*, Exon 1 | Quasar® 670 | AACTGACAAGCGCAACGTGT,  GTTATCTGCACTACTTTTAT,  CTGATTTACCACGTAACACA,  CGTAAACGTTTAACATTCCT,  GCGCGAAACGCCAAAGAGTT,  GGGCAGCACGCTTTAACATG,  TTGGTCACTTACACTAGGTC,  TATCGTCTTCTTTGTTGGTC,  CCTCATTTCTCGAAGTAACT,  CCAGGCTTGAAATCCGTTGT,  AAAACATACCGCTGGTTTGG,  ACCCAACAAATCTGCACAAA,  TTACTATTTCCTCAGGATCT,  TTGCTTTCATCCACAATACC,  ACCATTTACATTCTCAACAT,  TGGGACCTTTTTCCTCAAAT,  GCGTTACTTATCATTATGGC,  CGGAATCAGTTGGATATCCT,  TTTAAGCTTTTCCTGGTAGC,  ACAGAGCGTTGAATTTCAGT,  TCAACTAGATCCGACCTCAA,  GTCGATATACAACAGTCCAC,  ACCGAACGGTTATCAATCGA,  AAATATTGCCACCTCATCAC,  AAGCAAGAGTTTCGCTCTTC,  CGGCTTTAAAACGTGATCCA,  CAAATTCGGTCACAGCTTCT,  TGAGTTTGCTCTTTTTCTGC,  ACAGTTGCTTCAGATGATTT,  CCTTTAAATAGTTCATGCGA,  TTGCCATCTCACAAGTAATA,  GTATTTTTGAACTGGCTTCA,  TTCAACCAGTCGAACTCGTG,  TGTCGAAGCCATTGCTCAAA,  CCTCTACTAGTATACCTTGA,  GGGTGTATCTCTTTGTATTT,  ACATTTGCAAAGGTCCTACA,  AACATACGCCGCCAATTGAC,  TCCGTTATGGTTGTATATCT,  TAAATGCTCGGGATGTAACA,  ATGTCAAGCAATCCTTGTGG |
| *kl-3*, Exon 13 | Quasar® 670 | CCAATACTTTTGACCTTACA,  CGAGATCCATATTATATCCT,  AGGTGTTTTAATGCATCCAT,  GTTTCCTCTTGGATTACTTA,  CCTCCAACACCAACAAGTAA,  TTGTCAAGCTTTGTTTTCCG,  ATAGGACCGAGTTAAAGTCA,  AAGTCTTCGGTTAGATTTCC,  CCGGCCGTTCGATACAAAAA,  AAAGTCATGCCATTTCCATC,  TCTTTAATTTCATTGGCAGT,  AGGTTAGCAATTTCACCAGA,  CATCAAGCTCATCTTTAGCA,  CTGGAATTAGCTCGCTATAC,  CGAGGTTGGTGTTTTTTCAT,  TACAGATTGTCTTGCGTAGC,  GAAGATTATAGCGGGCACGA,  CTGGAGAAAAGCAGAGAGCT,  CGCATTCGAAATTTCTCTCC,  GGATATAAGGCCGGGAAACT,  GCCATTTTTGAAACCAGTCT,  ACTGCGATACGAGCATCTTC,  ATCAGTCAGATAATGGCGCG,  CCTGATCCTTAACTTTTTCA,  GTATCCAACTCATGATGTCA,  TCTCCGAAACCGATCGTAAT,  GACTTTGGCGTCACAAATGT,  ATGATCCTGTTTGTCTTTAT,  ACTCATTCGCTCTGACATTA,  CATCAAGTTTATCCAATCCA,  GAATAGCAACAGATGCACCT,  GCAAGAGCAATAACCTTGTT,  AAACGTCTTCTGCTTCTTCA,  TGCAGCTTTGGATTGTTCAA,  CCACTTCCACTTTTACTATT,  TTACAAGAACTTCCGCTTGG,  CCACATGTTTTACTGCTGAA,  AATGCCGGAAGAGCTTTTTC,  TTTAAGAGCAGCTTCTGCTT,  GCAATATCAGCCGCTTTAAT,  GTTTTCCCAATTTTCTGACA,  ACACACACAGTCCATTATTA,  ATAGGTTTTACTTTTCTTCT,  AAAGCTTTTTCCGTATCAGG,  TTCGTCCCAAGAACTTTGTA,  TCAGTAGGGTACTCTACTAT,  GACCATTTCGGCGTTAATTA,  CTGAAAATAGGGCACCATCA |
| *kl-3*, Exon 14 | Quasar® 570 | GTACTTTGACATAGCCATGG,  AAGATTTGCCTTTAAGGGCA,  TGATATTTAGCCTCTTGCAC,  CTGCTTCTTGTAGATCACTT,  CGTTTTCTTTTTGTTGCAGT,  TTTTTGGCCTCGTCTAATAC,  AACCACCAATAAGAGCGGTT,  TTCAGTCCATCGGATTTTTT,  CGGTCGGTCTCACTTTTAAA,  GGAGAATAACATCTCCGACC,  TCTTGATTAAATGGTCCCGT,  ATGTTCCACTCACCAATTTG,  TTGACAGTTCATCTGTTGGT,  TTTTAATCCACACTTTTCCC,  TAATCGGAATTCCCATGCTA,  TAACTCCTCTGCAACATCTT,  TTTCGGTCCAAAAGGTTGTC,  TCCAATCCACTTCTTTATCA,  GATTGGGTAGCTTTGTTGTA,  CGCGCAAATATTTCAGGTGT,  CCACGCATTGTCACAGTAAA,  GAACCCGTTCATCTTCTAAT,  TTTCATGTTTCCAGTCACAG,  TTCCTTTCGTAGTGGACAAC,  CCTCAATCACTGTAACGTCG,  TCCTTAACTTCAATGGCAGT,  CAGCGTTTATTTTTGCTTCT,  ACACTACCTCTTGTAGCAAC,  GCATCAAAGCGCTCAAGGAA,  TCCTCTAGATTTGTAGCGAT,  TTAACTCAAGCTGGCGATCA,  CGCACCGCCTTTTATAAATA,  CACCGAAACGGAACAGGAGG,  TATACCACGTAAACCAAGCC,  CCATACACCACGAACGAACA,  AGTTTAGTACTACTGGCTCG,  AATCATTGGCATAAGTTCCC,  AGCTCATTTTTTTTGGCGAG,  CTTGGCCCATAGAAATAGGA,  CGTCTTCTAGGCAGGATAAT,  TCCTAAGTGACAGTTTTGCA,  ACTGTAAGCTCTACCATGTA,  AGCAGGTGGTTCATTAGTAT,  TTTTTAGTCCAGCACGTATT,  TGGAGATTGCGAATAGTCCA,  TGCATACCAGTCGGATGAAT,  TCCTGCTCCTTAAAACTGTA,  CGTTTGCCATGTCATCAATA |
| *kl-5*, Exons 1-6 | Quasar® 670 | CTTCTTTTCCTTTTCGTCAG,  AAAAACTCCGGACGGTTGTC,  AGACGCATTGTCTTGGTTAG,  CATTTTAGTCCACTTATCCA,  AACATCCCTAAACTCGTTGG,  ATACGTTTCTTGTTGGGATT,  CCACCGGAATTGATTGTGAA,  CTGGAAAGCTGTAGGATGGA,  AGCAACTTTATACCGTGGTC,  GAAGTGGTGTTAAGTACCGA,  ATTTGCTAATGGGTTCGGTA,  ATCCCACTTGATTTACTGTG,  TCTGTCTTCATATCGTTTGC,  TCCATTTCGCATTTCTTGAG,  AACGAGACCTTTCATCTGGG,  CATGACTTCGTCCATGCAAA,  GTTAACCACGGAAATATCCC,  ACTCGAGCGAATGCTTCATA,  TCAAGCCACTTTACAACCAT,  TGCCTCTGGTAGTGGAAAAT,  CAAGTTCTCAAGGTTTTCCA,  AGTCTTTATACGCCTATCTC,  ACTTAGTTATGTCTCTAGCC,  CTTGTCCATTTTTCTTTTCA,  TCGTCCATATCGTTTGTTTC,  AGTGGTCGTATATCTGATCG,  CACTAATCCAATTGTCAGCA,  TGAAAATACCGCGAGTGACC,  GTTCTGTTGACACAGTCGAT,  GGAATATGGAGTCTGGTTCT,  TTATAGGCTTCGTCGACATC,  TGTAATACTCTAGGTGCTGC,  CAGTCCTGCGAGTTAAATGT,  ATCGTCCGAATATTTCATCC,  TCTTTTAGCTCTTCCAATCG,  CCCCATAACAATTTTTTCCA,  AAGGGCGAAAGTTATCTGCC,  TGCTGTTGTACTCTTCAAGA,  AATATTTGTCCACTCACGGT,  ACAGAGAATTTTCTGCGTCC,  TGGCCTTGAAGCTTAAACGA,  TGAAACGATACCATGCGCTC,  ATGTGTGTCTTCAAGACCTT,  GCGAAGAAGTAAGGAGCCTG,  GGGTTCGACATGTTTTTTGA,  ATCATCCACAATGACATGCA,  TAACGCACATGATCTCTTCC,  CGAGCGCCGTAAAAACGTTT |
| *kl-5*, Exons 16-17 | Quasar® 570 | TTAGTCTTGACCCACTTTAT,  TAAGTACTACCAACCCAGTT,  ACTGCTCTCTCAACTTGATC,  CGTCCTAGGAGAGGATTTAG,  GTGGGTTTGCTAATTTCGTA,  CCGGGTTACGGTAAAGTTTA,  CCACTTTAACTACTTCAGCT,  TCTCCAGCAGAAGATAAGCG,  CCAGAGTGACATCTTCAAGG,  GACTTCTATTTCGTCAGCAG,  GCATGGCATTGTTAAACACC,  CTATCAGATTCTCAACGCGG,  CGTGAGGTGTACACAAAGCT,  GCTCTACTTCACCAAGATTT,  AGCCATGTGAAATTGGACGT,  TGAATACTGCCTGGTTGTTA,  TCCTTCAATGTCCTTTTCAA,  TTTTCAGGACTCTCTGAGTC,  ATGCATCGCATTATGCAGAG,  CAATAAAGGAGCGCATGGCG,  GAATTCCATAGAGCGAGCAT,  TCTGGACTAGATTCCTCGAA,  ATTTCAATGGCGTTTTCTGC,  CGCGACCAGGTGAATATTTT,  CGATAGCTTGTGTGGACATT,  GATATGAGCAGCAGGATCAC,  GATTCAAGTATTCCTTGGGG,  ATCTAAGGCCTTATGGATGT,  CGAGCACATTTCTAGAGTCT,  TAAACGGGTAGCTACGGTTC,  TGATATTGTCAAATCGCCCA,  TCCAGGTAATTGTACAGCAC,  CTGTAATGTGACCTCCATAC,  GGAAAACCCTGGCAATACTC,  TGTAATTGTGGTAGCCAGTA,  AAGGCCGTATAGAGATGGAC,  AGTCGTTCAGAAACCGTAGT,  TCTCGGCTGCAACTCGAAAA,  TGTCTTCCTGTGAAACTGTC,  AATGGTGTGGGAGTTTTGTC,  CGCTCACACTCTTGAAATGC,  AGCGCTTCAATTCAGTCATA,  TGAAGGCATGCGAAAGTCGG,  TGCATGATAGCTGTGAGGAG,  GACACATTCTATCGAGAGGC,  TTCCACTTTTTGGTGACATC,  CAATGGTTCCCATTTTCATG,  AAGGTCCAAACGAAGGTGGG |
| *ks-1 (ory)*, All exons | Quasar® 670 | TTTTTGGCTTTCTTTCTGTC,  AAAAGTTGAGGCTCCGAGTT,  AAAACTGGACTACCCGGTTA,  CGTTTAATTCGCGATGCTTC,  CTTCATCTACATACCGACGA,  TCCGATGTTGAAGTCAGTTC,  TGATGCATCTGATTCTTTCC,  TCTTGCACTAACTGTTCTCG,  AATTCAGCTGTGTCATCAGT,  TTAGCTCGAGAAGCCATTTT,  ATTCAACGTTTAGGCGTTCG,  CCATTGTCTTCATCAAAGCT,  TTTCCATGTGCTTCTTTTTG,  CTCTATTCGCAATATCCAGT,  AGTTTACGTGTCGTTTCTGA,  GCATTGCCTTATTTAATGCG,  AAGCCTGCTCGTTAATTGAG,  TTGTACATTCTTGTTGTCGC,  CATCCTTCTCCAGAGAATTA,  GACGAATATCATCCGTTCGG,  CACGTTGCAATGTCTCTTTG,  ATGTTTTAAGTCCGCCATAG,  TCTTTCTCTGCTTTGGATTT,  CGTCTTCAGAGAGATTTGGT,  TCGTGTAACTTTTCCGTGAG,  ACGGACTCCAACTGTTTTTT,  CGCTGAAGCATCATTTTCTC,  TTTGATGTCCTGATTTTGCT,  TTTTACTTCGTTTGCGCTTA,  GCCACTAAGTTTTCTTTGTT,  TTTCTCTCATATCCTTACGT,  CAATCCGACTAGGTTACGTT,  AACAATCTCGTCATTCCGTC,  GGGCATTTTGTGCAATTTGA,  AAGGCATTCACTTTCAGTGT,  CTCATGTCAGCAGTACTTTT,  TCTTCAGTTAGAGCTCGTAT,  CAACGATGTACTCCTGTAGG,  AATTTCTTAGGATCTTCGCC,  TATTGACTGCTTTAGGGAGC,  AGCCTCACACAGTTTATTTT,  GCATATGAGATAGCATCCTT,  TAATGAACGCTCTGCTGCTA,  GTCTTGATTTAAGTTCCACC,  AATTGACAGCTCTCCGATTT,  TATTTCGTTTTTCCCATCTG,  ATCCACATTCCATATACTCT,  ATATCCGTTAACTTCGCACA |
